# Supplementary material for: Amide-to-ester substitution as a stable alternative to N-methylation for increasing membrane permeability in cyclic peptides
Source: Nat Commun. 2023 Mar 17;14:1416. doi: 10.1038/s41467-023-36978-z (PMC10023679; doi:10.1038/s41467-023-36978-z)
Supplement: Supplementary file 1 — Supplementary Information [file 41467_2023_36978_MOESM1_ESM.pdf]

Supplementary Information

Amide-to-ester substitution as a stable alternative to *N*-methylation for increasing membrane permeability in cyclic peptides

Yuki Hosono[1], Satoshi Uchida[1], Moe Shinkai[1], Chad E. Townsend[2], Colin N. Kelly[2], Matthew R. Naylor[2], Hsiau-Wei Lee[2], Kayoko Kanamitsu[3], Mayumi Ishii[3], Ryosuke Ueki[1], Takumi Ueda[3], Koh Takeuchi[3], Masatake Sugita[4,5], Yutaka Akiyama[4,5,*], R. Scott Lokey[2,*], Jumpei Morimoto[1,*] & Shinsuke Sando[1,6,*]

[1]*Department of Chemistry and Biotechnology, Graduate School of Engineering, The University of Tokyo, 7-3-1 Hongo, Bunkyo-ku, Tokyo 113-8656, Japan*

[2]*Department of Chemistry and Biochemistry, University of California, Santa Cruz, California 95064, United States*

[3]*Graduate School of Pharmaceutical Sciences, The University of Tokyo, 7-3-1 Hongo, Bunkyo-ku, Tokyo, 113-0033, Japan.*

[4]*Department of Computer Science, School of Computing, Tokyo Institute of Technology, 2-12-1 Ookayama, Meguro-ku, Tokyo 152-8550, Japan*

[5]*Middle-Molecule IT-based Drug Discovery Laboratory (MIDL), Tokyo Institute of Technology, 2-12-1 Ookayama, Meguro-ku, Tokyo 152-8550, Japan*

[6]*Department of Bioengineering, Graduate School of Engineering, The University of Tokyo, 7-3-1 Hongo, Bunkyo-ku, Tokyo 113-8656, Japan*

Email: akiyama@c.titech.ac.jp, slokey@ucsc.edu, jmorimoto@chembio.t.u-tokyo.ac.jp, ssando@chembio.t.u-tokyo.ac.jp

Table of contents

Supplementary Notes *5*

Supplementary Methods *6*

Synthesis of (S)-3-[4-tert-butoxyphenyl]-2-hydroxy-propionic acid (HO-Tyr(tBu)-OH) 6

Synthesis of Dimer peptides (P1–3) 6

Dimer depsipeptides (D1–3) 7

Dimer N-methylated peptides (M1–3) 7

Cyclic peptides (CP1–6, CP1-Y2F-L2K, CP1-Y2F-L2S, D8.31-amide, D8.21-amide, and D9.19-amide) 8

Cyclic peptides with amide-to-ester substitutions (DP1-5, CP2-6YE, CP3-6YE, CP4-6YE, CP5-6YE, CP6-6YE, CP1-Y1F-L2S (ester), CP1-Y1F-L2K (ester), D8.31-ester, D8.21-ester, and D9.16-ester) 9

Cyclic N-methylated peptides (MP1-5, CP2-6YM, CP3-6YM, CP4-6YM, CP5-6YM, CP6-6YM, CP1-Y1F-L2S (N-methyl), DP1-Y1F-L2K (N-methyl), D8.31, D8.21, and D9.16) 10

Chloroalkane tagged peptides (CP1-L2Kct and DP1-L2Kct) 11

Cell culture and transfection 13

Purity check of synthesized compounds 13

Supplementary Figures and Tables *14*

Supplementary Figure 1. ^{1}H-NMR spectrum of DP2 for conformational analysis 14

Supplementary Figure 2. COSY spectrum of DP2 for conformational analysis 15

Supplementary Figure 3. TOCSY spectrum of DP2 for conformational analysis 16

Supplementary Figure 4. HSQC-NMR spectrum of DP2 17

Supplementary Figure 5. HMBC-NMR spectrum of DP2 18

Supplementary Figure 6. ROESY-NMR spectrum of DP2 19

Supplementary Figure 7. Comparison of the most stable conformations of DP2 and CP1. 20

Supplementary Figure 8. Caco-2 assay with high concentrations. 21

Supplementary Figure 9. Confocal microscope images of cells in CAPA. 22

Supplementary Figure 10. ^{1}H-NMR spectrum of CP1 for conformational determination 23

Supplementary Figure 11. COSY spectrum of CP1 for conformational determination 24

Supplementary Figure 12. TOCSY spectrum of CP1 for conformational determination 25

Supplementary Figure 13. HSQC spectrum of CP1 for conformational determination 26

Supplementary Figure 14. HMBC spectrum of CP1 for conformational determination 27

Supplementary Figure 15. ROESY spectrum of CP1 for conformational determination 28

Supplementary Figure 16. ^{1}H-NMR of DP1 for conformational determination 29

Supplementary Figure 17. COSY spectrum of DP1 for conformational determination 30

Supplementary Figure 18. TOCSY spectrum of DP1 for conformational determination 31

Supplementary Figure 19. HSQC spectrum of DP1 for conformational determination 32

Supplementary Figure 20. HMBC spectrum of DP1 for conformational determination 33

Supplementary Figure 21. ROESY spectrum of DP1 for conformational determination 34

Supplementary Figure 22. NMR solution structure of MP1. 35

Supplementary Figure 23. ^{1}H-NMR of MP1 for conformational determination 36

Supplementary Figure 24. COSY spectrum of MP1 for conformational determination 37

Supplementary Figure 25. TOCSY spectrum of MP1 for conformational determination 38

Supplementary Figure 26. HSQC spectrum of MP1 for conformational determination 39

Supplementary Figure 27. HMBC spectrum of MP1 for conformational determination 40

Supplementary Figure 28. ROESY spectrum of MP1 for conformational determination 41

Supplementary Figure 29. Comparison of the representative conformations in high dielectric solvents from NMR and enhanced sampling MD simulations. 42

Supplementary Figure 30. ^{1}H-NMR of CP1 in 50% DMSO/H_2O 43

Supplementary Figure 31. COSY-NMR of CP1 in 50% DMSO/D_2O 44

Supplementary Figure 32. TOCSY spectrum of CP1 in 50% DMSO/H_2O 45

Supplementary Figure 33. HSQC spectrum of CP1 in 50% DMSO/D_2O 46

Supplementary Figure 34. HMBC spectrum of CP1 in 50% DMSO/D_2O 47

Supplementary Figure 35. ROESY spectrum of CP1 in 50% DMSO/H_2O and 50% DMSO/D_2O 48

Supplementary Figure 36. ^{1}H-NMR of DP1 in 50% DMSO/H_2O 49

Supplementary Figure 37. COSY-NMR of DP1 in 50% DMSO/H_2O 50

Supplementary Figure 38. TOCSY spectrum of DP1 in 50% DMSO/H_2O 51

Supplementary Figure 39. HSQC spectrum of DP1 in 50% DMSO/D_2O 52

Supplementary Figure 40. HMBC spectrum of DP1 in 50% DMSO/H_2O 53

Supplementary Figure 41. ROESY spectrum of DP1 in 50% DMSO/H_2O and 50% DMSO/D_2O 54

Supplementary Figure 42. ^{1}H-NMR of MP1 in 50% DMSO/H_2O 55

Supplementary Figure 43. COSY-NMR of MP1 in 50% DMSO/D_2O 56

Supplementary Figure 44. TOCSY spectrum of MP1 in 50% DMSO/H_2O 57

Supplementary Figure 45. HSQC spectrum of MP1 in 50% DMSO/D_2O 58

Supplementary Figure 46. HMBC spectrum of MP1 in 50% DMSO/D_2O 59

Supplementary Figure 47. ROESY spectrum of MP1 in 50% DMSO/H_2O and 50% DMSO/D_2O 60

Supplementary Figure 48. The percentage of all conformations in each region in the two-dimensional PCA space from the MD simulations 61

Supplementary Figure 49. PAMPA of cyclic hexapeptides CP2–CP6 and their derivatives 62

Supplementary Figure 50. Degradation profiles of control peptides in SGF and SIF 63

Supplementary Figure 51. ^{1}H-NMR spectrum of HO-Tyr(tBu)-OH 64

Supplementary Figure 52. ^{13}C-NMR spectrum of HO-Tyr(tBu)-OH 65

Supplementary Figure 53. UPLC chromatograms of P1–3, D1–3, and M1–3 66
Supplementary Figure 54. UPLC chromatograms of CP1, DP1–5, and MP1–5 68
Supplementary Figure 55. UPLC chromatograms of CP2–6 and their N-methylated peptide and depsipeptide derivatives 70
Supplementary Figure 56. UPLC chromatograms of CP1-Y1F-L2S/K and their derivatives 73
Supplementary Figure 57. UPLC chromatograms of D8.31/D8.21/D9.16 and their derivatives 74
Supplementary Figure 58. UPLC chromatograms of CP1-L2Kct, DP1-L2Kct, and MP1-L2Kct 76
Supplementary Figure 59. UPLC chromatograms of DP1L-NH_2 77
Supplementary Figure 60. Histograms of flow cytometric analysis of CAPA 78
Supplementary Table 1. Permeability values (P_e), ALogP, UPLC retention time, and log $D_{dec/w}$ of P1–3, D1–3, and M1–3. 79
Supplementary Table 2. Evaluation of the effect of efflux transporters on permeability of DP2 on Caco-2 assay ... 80
Supplementary Table 3. Amide temperature coefficients of CP1, DP1, and MP1. 81
Supplementary Table 4. Water solubility and mouse plasma stability 82
Supplementary Table 5. NOE summary of CP1 in $CDCl_3$ 83
Supplementary Table 6. $^3J_{HNCH}$ coupling values of CP1 in $CDCl_3$ 84
Supplementary Table 7. NOE summary of CP1 in DMSO/H_2O = 1/1 85
Supplementary Table 8. $^3J_{HNCH}$ coupling values of CP1 in DMSO/H_2O = 1/1 86
Supplementary Table 9. NOE summary of DP1 in $CDCl_3$ 87
Supplementary Table 10. $^3J_{HNCH}$ coupling values of DP1 in $CDCl_3$ 88
Supplementary Table 11. NOE summary of DP1 in DMSO/H_2O = 1/1 89
Supplementary Table 12. $^3J_{HNCH}$ coupling values of DP1 in DMSO/H_2O = 1/1 90
Supplementary Table 13. NOE summary of MP1 in $CDCl_3$ 91
Supplementary Table 14. $^3J_{HNCH}$ coupling values of MP1 in $CDCl_3$ 92
Supplementary Table 15. NOE summary of MP1 in DMSO/H_2O = 1/1 93
Supplementary Table 16. $^3J_{HNCH}$ coupling values of MP1 in DMSO/H_2O = 1/1 94
Supplementary Table 17. NOE summary of DP2 in $CDCl_3$ 95
Supplementary Table 18. $^3J_{HNCH}$ coupling values of DP2 in $CDCl_3$ 96
Supplementary Reference *97*

Supplementary Notes

Chemicals used in this study were purchased from commercial suppliers as received. Preparative HPLC was performed on a Prominence HPLC system (Shimadzu) with a $5C_{18}$-MS-II column (Nacalai tesque, 10 mm I.D.×150 mm, 34355-91). UPLC-MS analysis was performed on an ACQUITYUPLC H-Class/SQD2 (Waters) using InertSustain AQ-C18 (GL Science, 2.1 I.D.×50 mm). NMR spectra for conformational determination of **CP1**, **DP1**, and **MP1** were recorded on a Bruker AVANCE-III HD 800 spectrometer (Bruker, Millerica, MA, USA), equipped with a cryogenic probe at 298 K. NMR spectra for HO-Tyr(tBu)-OH were recorded on an ECS-400 (JEOL) at 298 K. ESI-MS data were obtained using a micrOTOF II (Bruker Daltonics) or SQD2 (Waters). HeLa cells (RCB0007) and Caco-2 cells (HTB-37) were obtained from RIKEN cell bank and American Type Culture Collection (ATCC), respectively.

Supplementary Methods

Synthesis of (S)-3-[4-tert-butoxyphenyl]-2-hydroxy-propionic acid (HO-Tyr(tBu)-OH)

The compound was synthesized according to the previous report[1] with minor modifications. O-*tert*-butyl-L-tyrosine, or H_2N-Tyr(tBu)-OH (6.0 mmol, 1.4 g) was dissolved in 8:2 water:acetic acid (v/v, 60 mL) and cooled on ice. 6.0 mL (12 mmol) of 2 M sodium nitrite aqueous solution was added to the H_2N-Tyr(tBu)-OH solution dropwise on ice. After 15 minutes, the reaction solution was allowed to warm to room temperature and stirred overnight. The reaction was monitored using thin-layer chromatography (TLC). After finishing the reaction, the reaction was quenched with 0.42 mL (5.0 mmol) of 40% methylamine aqueous solution. The quenched solution was acidified up to pH 3 with 1 M aqueous hydrogen chloride and extracted with ethyl acetate (3 × 50 mL). The organic phase was dried using sodium sulfate, filtered, and evaporated under vacuum. The product was further purified by column chromatography on silica gel using 90:9:1 chloroform:methanol:acetic acid to give the objective compound as white solid. Yield 0.52 g (2.2 mmol, 40%). ^{1}H-NMR ($CDCl_3$ with 0.05% v/v TMS, 400 MHz): δ7.11–7.13 (d, 2H, J = 8.5 Hz), 6.90–6.93 (d, 2H, J = 8.5), 4.45–4.48 (dd, 1H, J = 4.1, 7.1 Hz), 3.11–3.15 (dd, 1H, J = 4.1, 14.2 Hz), 2.90–2.96 (dd, 1H, J = 7.1, 14.2 Hz), 1.32 (s, 9H). ^{13}C NMR ($CDCl_3$ with 0.05% v/v TMS, 400 MHz): δ176.2, 152.7, 129.2, 128.4, 122.7, 75.4, 69.5, 38.0, 27.2. HRMS (ESI-TOF MS) *m/z*: $[M + Na]^+$ Calcd. for $C_{13}H_{18}NaO_4^+$ 261.1103; Found 261.1116.

Synthesis of Dimer peptides (P1–3)

The peptide was manually synthesized on Sieber amide resin (0.55 mmol/g). Resin (20 mg, 11 μmol) was swelled with *N,N*-dimethylformamide (DMF) in a 6 mL fritted syringe with continuous shaking. Fmoc deprotection was performed by incubating the resin with 20% piperidine/DMF for 2 min with continuous shaking. After washing the resin with DMF, the residual Fmoc group was removed by incubating the resin with 20% piperidine/DMF for 8 min with continuous shaking, and the resin was washed with DMF three times. Fmoc-protected amino acids (Fmoc-Leu-OH or Fmoc-Phe-OH) (4 equivalent), HATU (4 equivalent), and *N,N*-diisopropylethylamine (DIPEA) (8 equivalent) were dissolved in 440 μL of DMF, and the solution was added to the resin. The resin was incubated for 1 h at room temperature with continuous shaking. After the reaction, the resin was washed with DMF three times. Fmoc group was removed in the same way as described above, and the N-terminal residue was coupled for 4 h using Fmoc-protected amino acids (Fmoc-Leu-OH or Fmoc-Phe-OH) (4 equivalent), HATU (4 equivalent), and DIPEA (8 equivalent) in 440 μL of DMF. After the reaction, the resin was washed with DMF three times. After removal of the Fmoc group, 0.5 M acetic anhydride and 1 M DIPEA in 1 mL of DMF were added to the resin. The resin was incubated for 1 h at room temperature with continuous shaking. After removal of the reaction solution, the resin was washed with DMF and dichloromethane (DCM) three times each. Peptides were cleaved from the resin by shaking the resin with 95% trifluoroacetic acid (TFA)/2.5% triisopropylsilane (TIPS)/2.5% water for 2 h twice. The solution was evaporated. 10% acetonitrile/water was added to the peptides, and the peptides were purified by HPLC. Purified peptides were dissolved in *N,N*-dimethylsulfoxide (DMSO), yielding 10 mM peptides solution as DMSO stock. The concentration was determined based on the weight of the peptide. Purified peptides were analyzed by ESI-MS and UPLC.

P1: LC purity > 99%. HRMS (ESI-TOF MS) *m/z*: $[M + Na]^+$ Calcd. for $C_{20}H_{23}N_3NaO_3^+$ 376.1637; Found 376.1642., **P2**: LC purity 94%. HRMS (ESI-TOF MS) *m/z*: $[M + Na]^+$ Calcd. for $C_{14}H_{27}N_3NaO_3^+$ 308.1950; Found 308.1953., **P3**: LC purity > 99%. HRMS (ESI-TOF MS) *m/z*: $[M + Na]^+$ Calcd. for $C_{17}H_{25}N_3NaO_3^+$ 342.1794; Found 342.1786. The chromatograms are shown in Supplementary Figure 53.

Dimer depsipeptides (D1–3)

The peptide was manually synthesized on Sieber amide resin (0.46 mmol/g). Resin (40 mg, 18.4 µmol) was swelled with DMF in a 6 mL fritted syringe with continuous shaking. Fmoc deprotection was performed by shaking the resin with 20% piperidine/DMF for 3 min. After washing the resin with DMF, the residual Fmoc group was removed by shaking the resin with 20% piperidine/DMF for 12 min, and the resin was washed with DMF three times. α-Hydroxy acids (L-Leucic acid or L-3-Phenyllactic acid) (8 equivalent), HATU (8 equivalent), and DIPEA (16 equivalent) were dissolved in 736 µL of DMF, and the solution was added to the resin. The resin was shaken for 3 h at room temperature. After the reaction, the resin was washed with DMF and tetrahydrofuran (THF) three times each. The coupling of Fmoc-protected amino acids to α-hydroxy acid immobilized on the resin was performed according to a previous report.[2] Fmoc-amino acids (Fmoc-Leu-OH or Fmoc-Phe-OH) (8 equivalent), diisopropylcarbodiimide (DIC) (8 equivalent), and *N,N*-dimethylaminopyridine (DMAP) (0.2 equivalent) were dissolved in 736 µL of THF, and the solution was added to the resin. The resin was shaken for 2.5 h at room temperature. After the reaction, the resin was washed with THF and DMF three times each. Fmoc deprotection was performed by shaking the resin with 20% piperidine/DMF for 2 min. After washing the resin with DMF, the residual Fmoc group was removed by shaking the resin with 20% piperidine/DMF for 8 min, and the resin was washed with DMF three times. Acetic acid (4 equivalent), Oxyma (4 equivalent), and DIC (4 equivalent) were dissolved in 368 µL of DMF and preincubated for 10 min, and the solution was added to the resin. The resin was incubated for 1.5 h at room temperature with continuous shaking. Subsequent synthesis was conducted in the same manner as the synthesis of **P1–3**. Purified peptides were dissolved in DMSO, yielding 10 mM peptides solution as DMSO stock. The concentration was determined based on the weight of the peptide. Purified peptides were analyzed by ESI-MS and UPLC.

D1: LC purity 98%. HRMS (ESI-TOF MS) *m/z*: $[M + Na]^+$ Calcd. for $C_{20}H_{22}N_2NaO_4^+$ 377.1478; Found 377.1487., **D2**: LC purity > 99%. HRMS (ESI-TOF MS) *m/z*: $[M + Na]^+$ Calcd. for $C_{14}H_{26}N_2NaO_4^+$ 309.1791; Found 309.1798., **D3**: LC purity 98%. HRMS (ESI-TOF MS) *m/z*: $[M + Na]^+$ Calcd. for $C_{17}H_{24}N_2NaO_4^+$ 343.1634; Found 343.1625. The chromatograms are shown in Supplementary Figure 53.

Dimer N-methylated peptides (M1–3)

The peptide was manually synthesized on Sieber amide resin (0.46 mmol/g). Resin (40 mg, 18.4 µmol) was swelled with DMF in a 2 mL fritted syringe with continuous shaking. Fmoc deprotection was performed by shaking the resin with 20% piperidine/DMF for 3 min. After washing the resin with DMF, the residual Fmoc group was removed by shaking the resin with 20% piperidine/DMF for 12 min, and the resin was washed with DMF three times. Fmoc protected *N*-methylamino

acids (Fmoc-NMeLeu-OH or Fmoc-NMePhe-OH) (8 equivalent), HATU (8 equivalent), and DIPEA (16 equivalent) were dissolved in 736 μL of DMF, and the solution was added to the resin. The resin was incubated for 3 h at room temperature with continuous shaking. After the reaction, the resin was washed with DMF three times. After removal of the Fmoc group, N-terminal residue was coupled for 3 h using Fmoc-protected amino acids (Fmoc-Leu-OH or Fmoc-Phe-OH) (8 equivalent), HATU (8 equivalent), and DIPEA (16 equivalent) in 736 μL of DMF. After the reaction, the resin was washed with DMF three times. Subsequent synthesis was the same as the synthesis of **D1–3**. Purified peptides were dissolved in DMSO, yielding 10 mM peptides solution as DMSO stock. The concentration was determined based on the weight of the peptide. Purified peptides were analyzed by ESI-MS and UPLC.

M1: LC purity 99%. HRMS (ESI-TOF MS) *m/z*: $[M + Na]^+$ Calcd. for $C_{21}H_{25}N_3NaO_3^+$ 390.1794; Found 390.1792., **M2**: LC purity 97%. HRMS (ESI-TOF MS) *m/z*: $[M + Na]^+$ Calcd. for $C_{15}H_{29}N_3NaO_3^+$ 322.2107; Found 322.2106., **M3**: LC purity 96%. HRMS (ESI-TOF MS) *m/z*: $[M + Na]^+$ Calcd. for $C_{18}H_{27}N_3NaO_3^+$ 356.1950; Found 356.1952. The chromatograms are shown in Supplementary Figure 53.

Cyclic peptides (CP1–6, CP1-Y1F-L2S, CP1-Y1F-L2K, D8.31-amide, D8.21-amide, and D9.16-amide)

The hexapeptides were manually synthesized on 2-chlorotriryl chloride resin (1.6 mmol/g). For example, **CP1** (cyclo[Tyr-Leu-D-Leu-Leu-Leu-D-Pro]) was synthesized as follows. Resin (20 mg, 32 μmol) was swelled with DCM in a 6 mL fritted syringe with continuous shaking. Fmoc-Leu-OH (2 equivalent) and DIPEA (4 equivalent) were dissolved in 640 μL DCM, and the solution was added to the resin. The resin was shaken overnight at room temperature. After the reaction, the resin was washed with DCM and 17:2:1 DCM:methanol:DIPEA three times each. The loading was quantified according to a previous report.[3] The resin was applied to further peptide synthesis. Fmoc deprotection was performed by shaking the resin with 20% piperidine/DMF for 3 min. After washing the resin with DMF, the residual Fmoc group was removed by shaking the resin with 20% piperidine/DMF for 12 min, and the resin was washed with DMF three times. The coupling reaction was performed by shaking the resin with Fmoc-protected amino acid (4 equivalent), HATU (4 equivalent), and DIPEA (8 equivalent) in DMF (0.1 M with respect to Fmoc-protected amino acid) for 1–1.5 h. After the reaction, the resin was washed with DMF three times. Above deprotection and coupling reactions were repeated until removal of the Fmoc group of N-terminal Leu-5. The resin was washed with DMF and DCM three times each. The linear precursor peptide was cleaved from the resin by shaking the resin with 20% 2,2,2-trifluoroethanol/DCM for 30 min twice. The filtrate was collected and evaporated. The peptide was dissolved in 30% acetonitrile/water and purified by HPLC. The purified peptide was lyophilized. For **CP2–6**, this purification step was omitted. The lyophilized peptide, PyAOP (1.5 equivalent), HOAt (1.5 equivalent), and DIPEA (4.5 equivalent) were dissolved to DMF (2 mM with respect to the peptide) and stirred overnight at room temperature to cyclize the peptide. After the reaction, the solvent was evaporated, and 50% TFA/45% DCM/2.5% TIPS/2.5% water was added and stirred 2 h at room temperature to perform the side chain deprotection. After deprotection, the solvent was evaporated. The peptide was dissolved to 30% acetonitrile/water, purified by HPLC, and lyophilized. After lyophilization, the purified peptide was dissolved in DMSO, yielding 10 mM **CP1** solution based on the UV absorbance

at 280 nm as DMSO sock. Purified **CP1** was analyzed by ESI-MS and UPLC.

The 8-mer and 9-mer peptides were synthesized in 100 μmol scale using an automated peptide synthesizer (Syro I, Biotage). The C-terminal amino acid was first loaded on 2-chlorotriryl chloride resin with the same procedure with that used for **CP1**. The following peptides synthesis was conducted on Syro I using COMU as a coupling reagent. The synthesized linear peptides were cleaved from resin using 20% 1,1,1,3,3,3-hexafluoro-2-propanol in DCM. The peptides were cyclized using the same procedure for **CP1** without a prior purification. The cyclized peptides were purified by HPLC and lyophilized. The purified peptides were analyzed by UPLC-MS.

CP1: LC purity 96%. HRMS (ESI-TOF MS) *m/z*: $[M + Na]^+$ Calcd. For $C_{38}H_{60}N_6NaO_7^+$ 735.4421; Found 735.4419. **CP2**: LC purity > 99%. HRMS (ESI-TOF MS) *m/z*: $[M + Na]^+$ Calcd. For $C_{38}H_{60}N_6NaO_7^+$ 735.4421; Found 735.4419. **CP3**: LC purity > 99%. HRMS (ESI-TOF MS) *m/z*: $[M + Na]^+$ Calcd. For $C_{38}H_{60}N_6NaO_7^+$ 735.4421; Found 735.4423. **CP4**: LC purity 94%. HRMS (ESI-TOF MS) *m/z*: $[M + Na]^+$ Calcd. For $C_{38}H_{60}N_6NaO_7^+$ 735.4421; Found 735.4440. **CP5**: LC purity > 99%. HRMS (ESI-TOF MS) *m/z*: $[M + Na]^+$ Calcd. For $C_{38}H_{60}N_6NaO_7^+$ 735.4421; Found 735.4440. **CP6**: LC purity > 99%. HRMS (ESI-TOF MS) *m/z*: $[M + Na]^+$ Calcd. For $C_{38}H_{60}N_6NaO_7^+$ 735.4421; Found 735.4433. **CP1-Y1F-L2S**: LC purity > 99%. HRMS (ESI-TOF MS) *m/z*: $[M + Na]^+$ Calcd. for $C_{35}H_{54}N_6NaO_7^+$ 693.3946; Found 693.3926. **CP1-Y1F-L2K**: LC purity 94%. HRMS (ESI-TOF MS) *m/z*: $[M + Na]^+$ Calcd. for $C_{38}H_{61}N_7NaO_6^+$ 734.4576; Found 734.4562. **D8.31-amide**: LC purity 92%. HRMS (ESI-TOF MS) *m/z*: $[M + Na]^+$ Calcd. for $C_{39}H_{66}N_8NaO_8^+$ 797.4896; Found 797.4915. **D8.21-amide**: LC purity 95%. HRMS (ESI-TOF MS) *m/z*: $[M + Na]^+$ Calcd. for $C_{45}H_{78}N_8NaO_8^+$ 881.5835; Found 881.5848. **D9.16-amide**: LC purity > 99%. HRMS (ESI-TOF MS) *m/z*: $[M + Na]^+$ Calcd. for $C_{46}H_{79}N_9NaO_9^+$ 924.5893; Found 924.5868. The chromatograms are shown in Supplementary Figure 54–57.

Cyclic peptides with amide-to-ester substitutions (DP1-5, CP2-6YE, CP3-6YE, CP4-6YE, CP5-6YE, CP6-6YE, CP1-Y1F-L2S (ester), CP1-Y1F-L2K (ester), D8.31-ester, D8.21-ester, and D9.16-ester)

The 6-mer depsipeptides were manually synthesized on 2-chlorotriryl chloride resin (1.6 mmol/g). The ester bond was located at N-terminus in the linear precursor peptide because the repeated piperidine treatment may cause racemization and/or hydrolysis. For example, when it comes to cyclo[^{O}Tyr-Leu-D-Leu-Leu-Leu-D-Pro] (**DP1)**, H-D-Pro-^{O}Tyr-Leu-D-Leu-Leu-Leu-OH was synthesized as the linear precursor peptide. The coupling of Fmoc-protected amino acids to α-hydroxy acid was performed according to a previous report.[2] The peptide synthesis method was the same as that for the synthesis of **CP1**. Coupling of α-hydroxy acids was performed by shaking the resin with α-hydroxy acid (4 equivalent), HATU (4 equivalent), and DIPEA (8 equivalent) in DMF (0.2 M with respect to hydroxy acid) for 2 h at room temperature. After the reaction, the resin was washed with DMF and THF three times each. The resin was shaken with Fmoc-protected amino acid (4 equivalent), DIC (4 equivalent), and DMAP (0.1 equivalent) in THF (0.1 M with respect to Fmoc-protected amino acid) for 2 h at room temperature. After the reaction, the resin was washed with THF and DMF three times each. Subsequent synthetic procedures were the same as those for the synthesis of **CP1**. Purified peptides were analyzed by ESI-MS and UPLC.

The 8-mer and 9-mer depsipeptides were synthesized in 100 mmol scale using an automated peptide synthesizer (Syro I, Biotage). The C-terminal amino acid was first loaded on 2-chlorotriryl chloride resin with the same procedure with that for **CP1**. The following peptides synthesis was conducted on Syro I using COMU as a coupling reagent. The coupling reactions of a hydroxy acid and the following N-terminal amino acid were conducted manually with the same procedure used for **DP1**. The synthesized linear peptides were cleaved from resin using 20% 1,1,1,3,3,3-hexafluoro-2-propanol in DCM. The cleaved peptides were dissolved purified by HPLC. The purified peptides were lyophilized. The peptides were cyclized using the same procedure for **CP1**. The cyclized peptides were purified by HPLC and lyophilized. The purified peptides were analyzed by UPLC-MS.

DP1: LC purity 87%. HRMS (ESI-TOF MS) *m/z*: $[M + Na]^+$ Calcd. for $C_{38}H_{59}N_5NaO_8^+$ 736.4261; Found 736.4285. **DP2**: LC purity > 99%. HRMS (ESI-TOF MS) *m/z*: $[M + Na]^+$ Calcd. for $C_{38}H_{59}N_5NaO_8^+$ 736.4261; Found 736.4291. **DP3**: LC purity > 99%. HRMS (ESI-TOF MS) *m/z*: $[M + Na]^+$ Calcd. for $C_{38}H_{59}N_5NaO_8^+$ 736.4261; Found 736.4262. **DP4**: LC purity > 99%. HRMS (ESI-TOF MS) *m/z*: $[M + Na]^+$ Calcd. for $C_{38}H_{59}N_5NaO_8^+$ 736.4261; Found 736.4268. **DP5**: LC purity > 99%. HRMS (ESI-TOF MS) *m/z*: $[M + Na]^+$ Calcd. for $C_{38}H_{59}N_5NaO_8^+$ 736.4261; Found 736.4282. **CP2-6YE**: LC purity 98%. HRMS (ESI-TOF MS) *m/z*: $[M + Na]^+$ Calcd. for $C_{38}H_{59}N_5NaO_8^+$ 736.4261; Found 736.4282. **CP3-6YE**: LC purity > 99%. HRMS (ESI-TOF MS) *m/z*: $[M + Na]^+$ Calcd. for $C_{38}H_{59}N_5NaO_8^+$ 736.4261; Found 736.4277. **CP4-6YE**: LC purity > 99%. HRMS (ESI-TOF MS) *m/z*: $[M + Na]^+$ Calcd. for $C_{38}H_{59}N_5NaO_8^+$ 736.4261; Found 749.4266. **CP5-6YE**: LC purity > 99%. HRMS (ESI-TOF MS) *m/z*: $[M + Na]^+$ Calcd. for $C_{38}H_{59}N_5NaO_8^+$ 736.4261; Found 736.4266. **CP6-6YE**: LC purity > 99%. HRMS (ESI-TOF MS) *m/z*: $[M + Na]^+$ Calcd. for $C_{38}H_{59}N_5NaO_8^+$ 736.4261; Found 736.4265. **CP1-Y1F-L2S (ester)**: LC purity 98%. HRMS (ESI-TOF MS) *m/z*: $[M + Na]^+$ Calcd. for $C_{35}H_{53}N_5NaO_8^+$ 694.3786; Found 694.3773. **CP1-Y1F-L2K (ester)**: LC purity 99%. HRMS (ESI-TOF MS) *m/z*: $[M + Na]^+$ Calcd. for $C_{38}H_{60}N_6NaO_7^+$ 735.4416; Found 735.4423. **D8.31-ester**: LC purity 96%. HRMS (ESI-TOF MS) *m/z*: $[M + Na]^+$ Calcd. for $C_{39}H_{65}N_7NaO_9^+$ 798.4736; Found 798.4737. **D8.21-ester**: LC purity > 99%. HRMS (ESI-TOF MS) *m/z*: $[M + Na]^+$ Calcd. for $C_{45}H_{77}N_7NaO_9^+$ 882.5675; Found 882.5665. **D9.16-ester**: LC purity > 99%. HRMS (ESI-TOF MS) *m/z*: $[M + Na]^+$ Calcd. for $C_{46}H_{78}N_8NaO_{10}^+$ 925.5733; Found 925.5739. The chromatograms are shown in Supplementary Figure 54–57.

Cyclic N-methylated peptides (MP1-5, CP2-6YM, CP3-6YM, CP4-6YM, CP5-6YM, CP6-6YM, CP1-Y1F-L2S (N-methyl), DP1-Y1F-L2K (N-methyl), D8.31, D8.21, and D9.16)

The 6-mer *N*-methylated peptides were manually synthesized on 2-chlorotriryl chloride resin (1.6 mmol/g). The *N*-methylamide bond was located at N-terminus in precursor linear peptide to prevent diketopiperazine formation followed by the cleavage of peptides. For example, when it comes to cyclo[^{NMe}Tyr-Leu-D-Leu-Leu-Leu-D-Pro] (**MP1)**, H-D-Pro-^{NMe}Tyr-Leu-D-Leu-Leu-Leu-OH was synthesized as the linear precursor peptide. On-resin *N*-methylation of amides was performed according to a previous report[4] with minor modifications. The peptide synthesis method was the same as that for the synthesis of **CP1**. After deprotection of the Fmoc group at the corresponding amino group, the resin was shaken with *o*-nirobenzenesulfonyl chloride (*o*-NBS-Cl) (4 equivalent) and 2,4,6-collidine (4 equivalent) in 1-methyl-2-pyrrolidinone (NMP) (0.1 M respect to *o*-NBS-Cl) for 15 min. After the reaction, the resin was washed with NMP and

THF three times each. *N*-methylation was performed by shaking the resin with triphenylphosphine (5 equivalent) and methanol (10 equivalent) in THF (0.2 M with respect to triphenylphosphine) for 1 min, then diisopropyl azodicarboxylate (5 equivalent) was added. The reaction was performed for 10 min and was performed again. After the reaction, the resin was washed with NMP and THF three times each. The deprotection of the *o*-nitrobenzenesulfonyl group was performed by shaking with mercaptoethanol (10 equivalent), and 1,8-diazabicyclo [5,4,0]undec-7-ene (DBU) (5 equivalent) in THF (0.4 M with respect to mercaptoethanol) for 10 min and the resin was washed with NMP three times. The deprotection was repeated twice. Subsequent synthesis was conducted in the same manner as the synthesis of **CP1**. Purified peptides were analyzed by ESI-MS and UPLC.

The 8-mer and 9-mer *N*-methylated peptides were synthesized in 100 μmol scale using an automated peptide synthesizer (Syro I, Biotage). The C-terminal amino acid was first loaded on 2-chlorotriryl chloride resin with the same procedure with that used for **CP1**. The following peptides synthesis was conducted on Syro I using COMU as a coupling reagent. The Fmoc-protected *N*-methylated amino acids were used for incorporation of *N*-methylated amino acid residues. The synthesized linear peptides were cleaved from resin using 20% 1,1,1,3,3,3-hexafluoro-2-propanol in DCM. The peptides were cyclized using the same procedure for **CP1** without a prior purification. The cyclized peptides were purified by HPLC and lyophilized. The purified peptides were analyzed by UPLC-MS.

MP1: LC purity > 99%. HRMS (ESI-TOF MS) *m/z*: $[M + Na]^+$ Calcd. for $C_{39}H_{62}N_6NaO_7^+$ 749.4578; Found 749.4583. **MP2**: LC purity > 99%. HRMS (ESI-TOF MS) *m/z*: $[M + Na]^+$ Calcd. for $C_{39}H_{62}N_6NaO_7^+$ 749.4578; Found 749.4596. **MP3**: LC purity > 99%. HRMS (ESI-TOF MS) *m/z*: $[M + Na]^+$ Calcd. for $C_{39}H_{62}N_6NaO_7^+$ 749.4578; Found 749.4598. **MP4**: LC purity > 99%. HRMS (ESI-TOF MS) *m/z*: $[M + Na]^+$ Calcd. for $C_{39}H_{62}N_6NaO_7^+$ 749.4578; Found 749.4564. **MP5**: LC purity 98%. HRMS (ESI-TOF MS) *m/z*: $[M + Na]^+$ Calcd. for $C_{39}H_{62}N_6NaO_7^+$ 749.4578; Found 749.4578. **CP2-6YM**: LC purity 79%. HRMS (ESI-TOF MS) *m/z*: $[M + Na]^+$ Calcd. for $C_{39}H_{62}N_6NaO_7^+$ 749.4578; Found 749.4550. **CP3-6YM**: LC purity 91%. HRMS (ESI-TOF MS) *m/z*: $[M + Na]^+$ Calcd. for $C_{39}H_{62}N_6NaO_7^+$ 749.4578; Found 749.4563. **CP4-6YM**: LC purity 93%. HRMS (ESI-TOF MS) *m/z*: $[M + Na]^+$ Calcd. for $C_{39}H_{62}N_6NaO_7^+$ 749.4578; Found 749.4579. **CP5-6YM**: LC purity 91%. HRMS (ESI-TOF MS) *m/z*: $[M + Na]^+$ Calcd. for $C_{39}H_{62}N_6NaO_7^+$ 749.4578; Found 749.4582. **CP6-6YM**: LC purity 85%. HRMS (ESI-TOF MS) *m/z*: $[M + Na]^+$ Calcd. for $C_{39}H_{62}N_6NaO_7^+$ 749.4578; Found 749.4577. **CP1-Y1F-L2S (*N*-methyl)**: LC purity > 99%. HRMS (ESI-TOF MS) *m/z*: $[M + Na]^+$ Calcd. for $C_{36}H_{56}N_6NaO_7^+$ 707.4103; Found 707.4086. **CP1-Y1F-L2K (*N*-methyl)**: LC purity 99%. HRMS (ESI-TOF MS) *m/z*: $[M + Na]^+$ Calcd. for $C_{39}H_{63}N_7NaO_6^+$ 748.4732; Found 748.4710. **D8.31**: LC purity > 99%. HRMS (ESI-TOF MS) *m/z*: $[M + Na]^+$ Calcd. for $C_{40}H_{68}N_8NaO_8^+$ 811.5052; Found 811.5047. **D8.21**: LC purity 97%. HRMS (ESI-TOF MS) *m/z*: $[M + Na]^+$ Calcd. for $C_{46}H_{80}N_8NaO_8^+$ 895.5991; Found 895.5979. **D9.16**: LC purity 98%. HRMS (ESI-TOF MS) *m/z*: $[M + Na]^+$ Calcd. for $C_{47}H_{81}N_9NaO_9^+$ 938.6049; Found 938.6059. The chromatograms are shown in Supplementary Figure 54–57.

Chloroalkane tagged peptides (CP1-L2Kct and DP1-L2Kct)

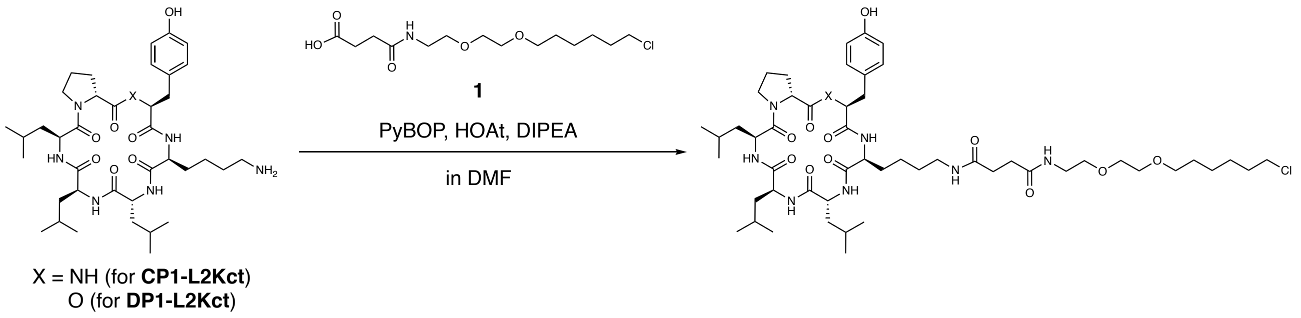

Scheme S1. Synthetic scheme of chloroalkane tagged cyclic peptides.

Compound **1**, the chloroalkane carboxylic acid, was synthesized according to the previous report.[5] Chloroalkane-tagged cyclic peptides were synthesized as shown in Scheme S1. Precursor cyclic peptides that have a Lys residue were synthesized in the same manner with the sections 2-5 and 2-6. The precursor cyclic peptides were then coupled with the chloroalkane carboxylic acid. For example, for the synthesis of **CP1-L2Kct**, the precursor peptide (1.4 mg), cyclo[Tyr-Lys-D-Leu-Leu-Leu-D-Pro] was dissolved in 40 μL of DMF and mixed with compound **1** (1 equivalent), PyBOP (1 equivalent), HOAt (1 equivalent), and DIPEA (3 equivalent). The reaction mixture was continuously shaken for 2 h at room temperature. After the reaction, the solution was diluted 100 times with 40% acetonitrile/water, and the objective compound was purified by HPLC. Purified peptides were analyzed by ESI-MS and UPLC.

CP1-L2Kct: LC purity > 99%. HRMS (ESI-TOF MS) *m/z*: $[M + Na]^+$ Calcd. for $C_{52}H_{85}ClN_8NaO_{11}^+$ 1055.5924; Found 1055.5940. **DP1-L2Kct**: LC purity 98%. HRMS (ESI-TOF MS) *m/z*: $[M + Na]^+$ Calcd. for $C_{52}H_{84}ClN_7NaO_{12}^+$ 1056.5764; Found 1056.5776. The chromatograms are shown in Supplementary Figure 58.

Cell culture and transfection

HeLa cells were cultured in growth medium (Roswell Park Memorial Institute medium (RPMI 1640) (Nalarai tesque) with 10% fetal bovine serum (FBS) (Cosmobio) and 1% Antibiotic-Antimycotic (Nakarai tesque)) at 37 °C with 5% CO_2 in a humidified atmosphere. pEBMulti-puro vector inserted with a gene that expresses Haloenzyme-GFP with a mitochondria-targeting sequence (HaloGFP-ActA) was constructed as follows. The mitochondrial targeting domain of the *Listeria monocytogenes* ActA gene was fused to the gene coding Haloenzyme-GFP. The constructed gene was inserted at the downstream of a CAG promoter of pEBMulti-puro vector (FUJIFILM Wako Chemicals), which contains a CAG promoter, a puromycin resistant gene, an EBNA1 coding gene, and an oriP coding gene for stable expression. 1×10^5 HeLa cells were transferred to a 6-well plate and cultured for 19 h. The cells were transfected with 4 μg of pEB-HaloGFP-ActA and 16 μg of polyethyleneimine in RPMI. One day after the transfection, the culture medium was changed to the one with 1 μg/mL puromycin for the selection of the stable cell lines.

Purity check of synthesized compounds

The purities of the products were analyzed on UPLC monitored at 220 nm. UPLC analysis was performed using a linear gradient of solvent A (water containing 0.1% TFA) and solvent B (acetonitrile containing 0.1% TFA). The blue line denotes the percentage of solvent B.

Supplementary Figures and Tables

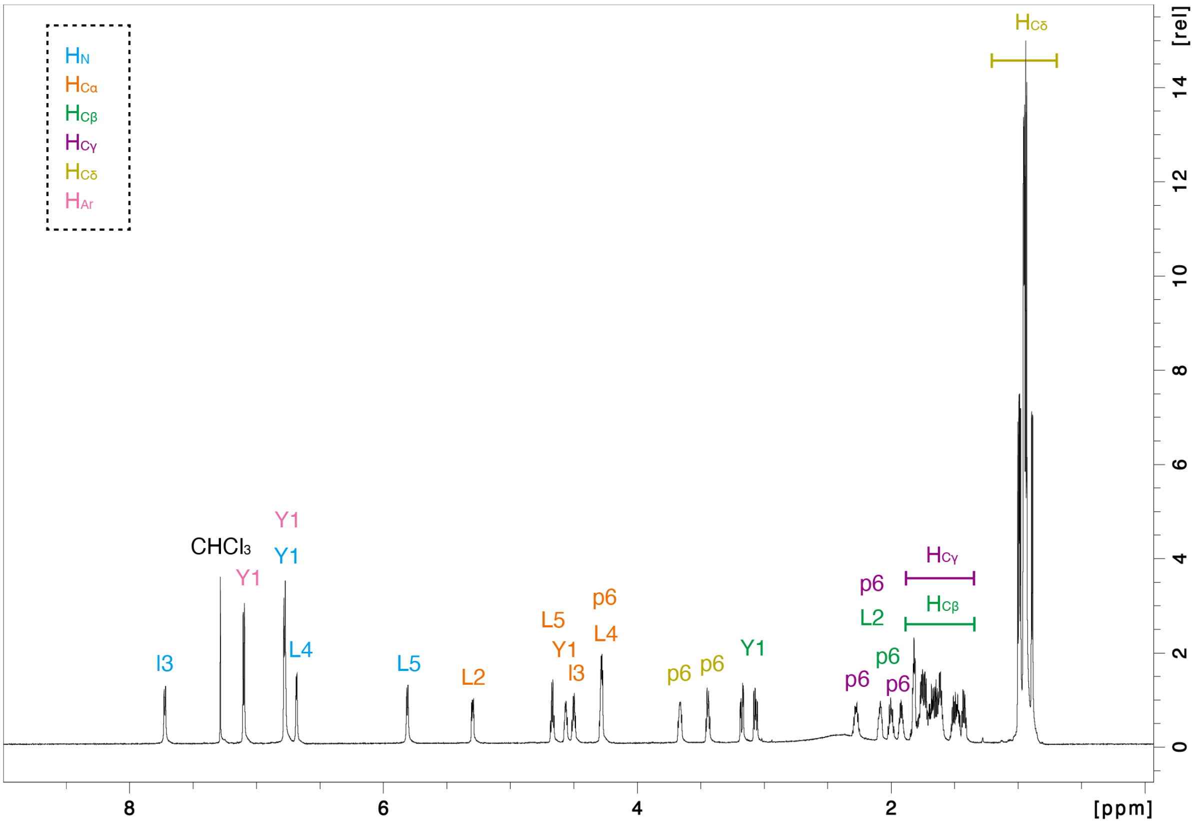

Supplementary Figure 1. ^{1}H-NMR spectrum of DP2 for conformational analysis

The ^{1}H-NMR spectrum of **DP2** was recorded in $CDCl_3$. Light blue, orange, and green letters denote peaks derived from H_N, $H_{C\alpha}$, $H_{C\beta}$, respectively. The one-letter residue code and the number above a peak indicate the residue number to which the proton belongs.

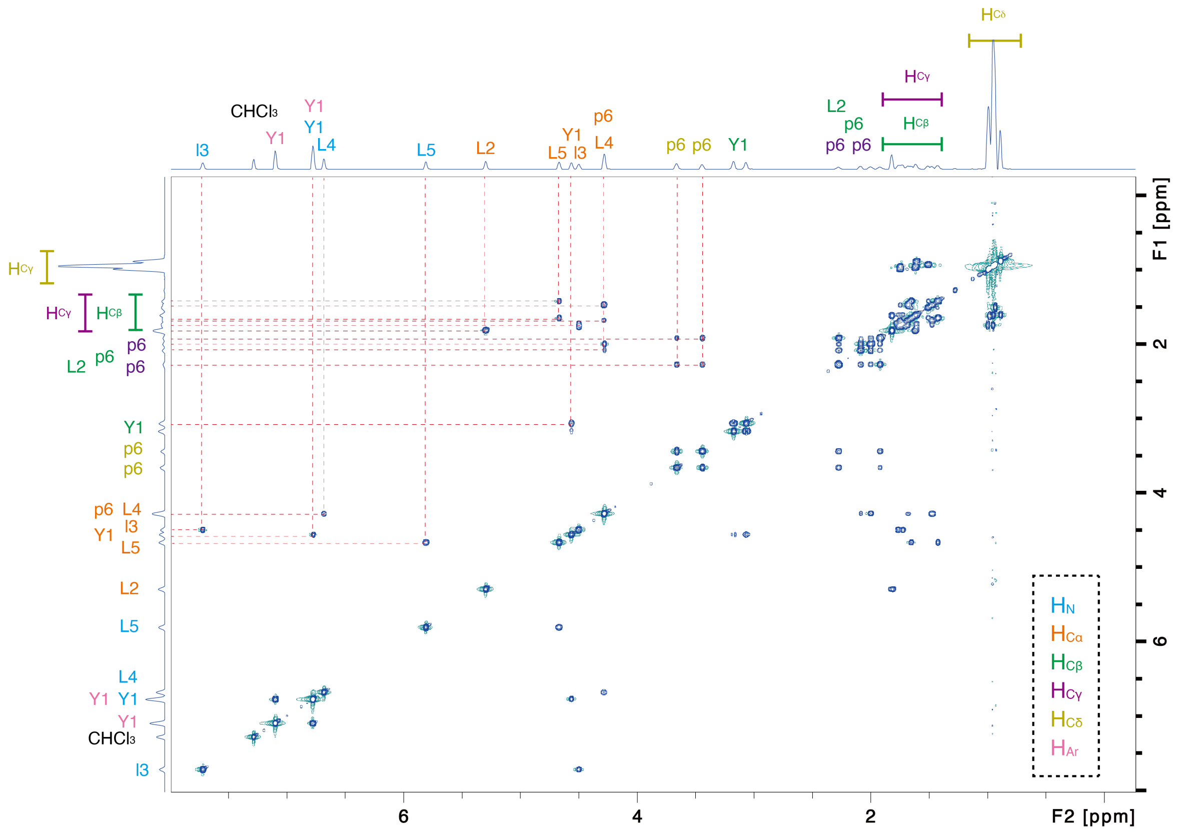

Supplementary Figure 2. COSY spectrum of DP2 for conformational analysis

The COSY-NMR spectrum of **DP2** was recorded in $CDCl_3$. Correlation peaks that support the assignment of ^{1}H-NMR are shown with red dashed lines.

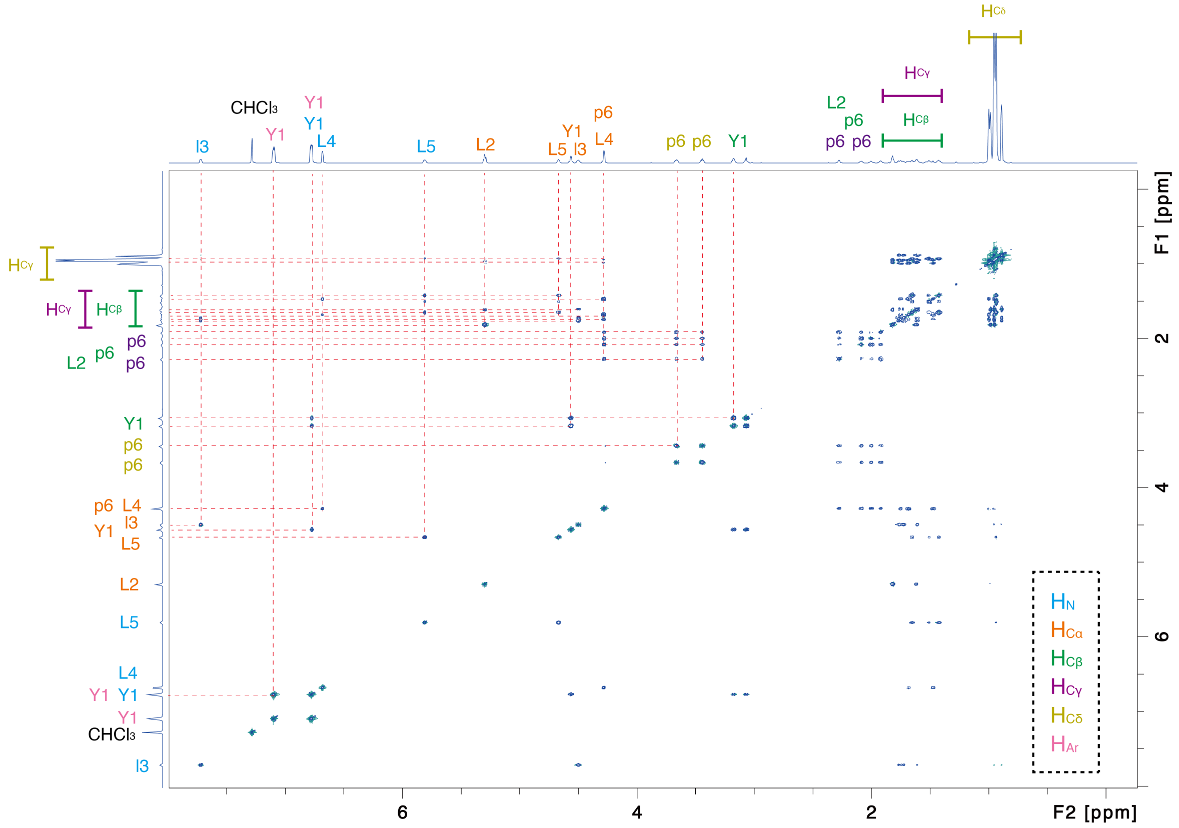

Supplementary Figure 3. TOCSY spectrum of DP2 for conformational analysis

The TOCSY-NMR spectrum of **DP2** was recorded in $CDCl_3$. Correlation peaks that support the assignment of ^{1}H-NMR are shown with red dashed lines.

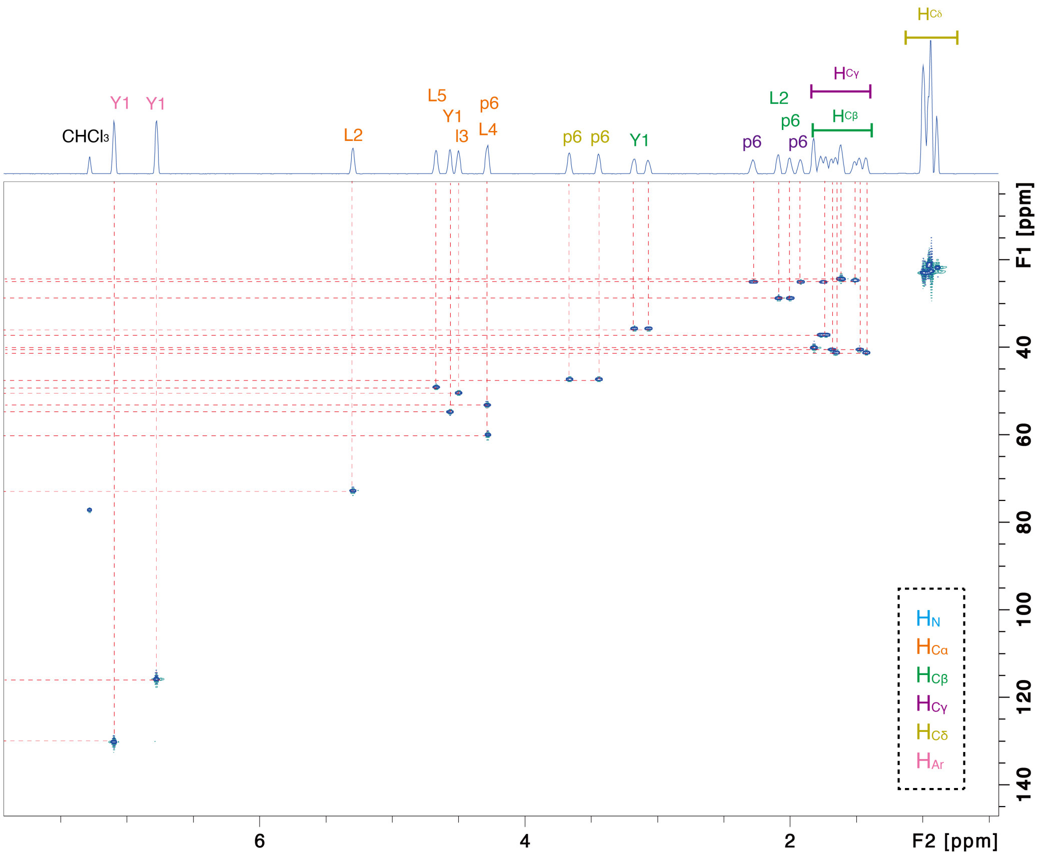

Supplementary Figure 4. HSQC-NMR spectrum of DP2

The HSQC-NMR spectrum of **DP2** was recorded in $CDCl_3$. The one-letter residue code and the number above a peak indicate the residue number to which the proton belongs.

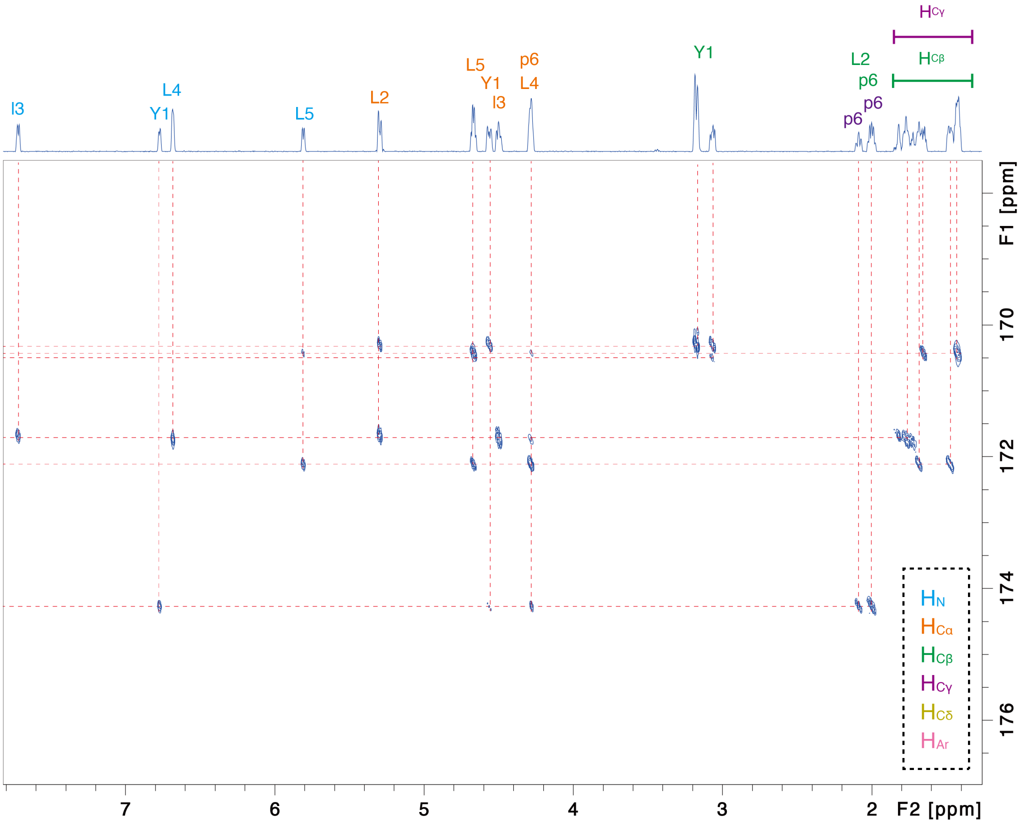

Supplementary Figure 5. HMBC-NMR spectrum of DP2

The HMBC-NMR spectrum of **DP2** was recorded in $CDCl_3$. The one-letter residue code and the number above a peak indicate the residue number to which the proton belongs.

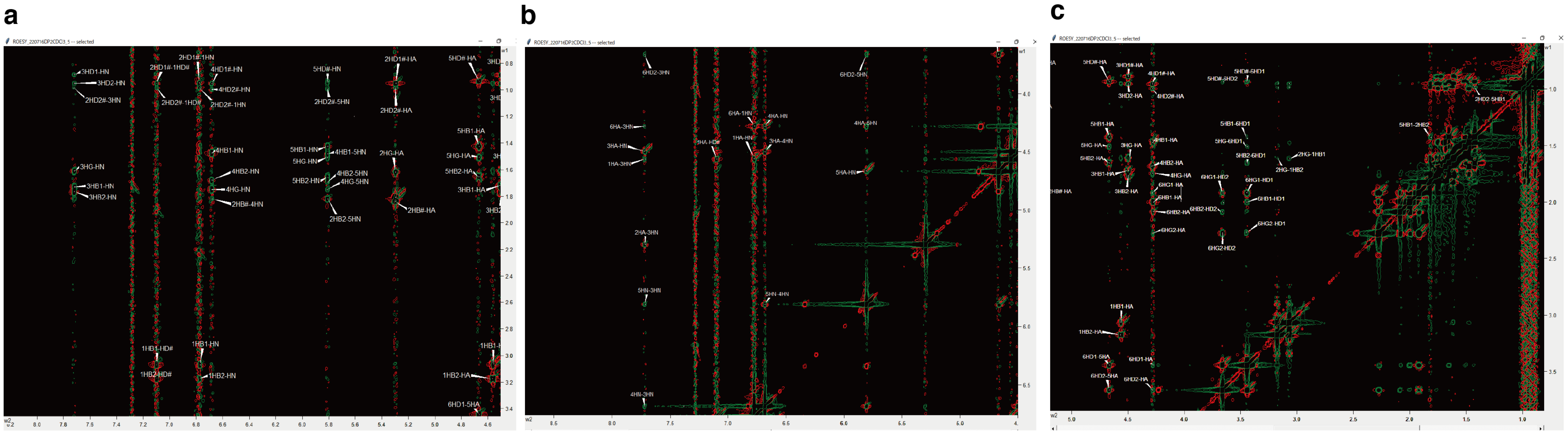

Supplementary Figure 6. ROESY-NMR spectrum of DP2

The ROESY-NMR spectrum of **DP2** was recorded in $CDCl_3$. (a–c) Magnified views of various areas. Correlation peaks indicating the proximities of protons are indicated with the names of the proximal two protons. The correlation peaks are summarized in a Supplementary Table.

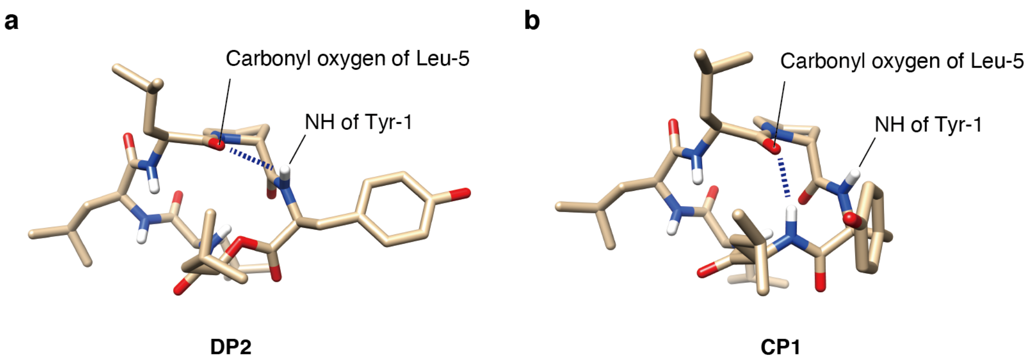

Supplementary Figure 7. Comparison of the most stable conformations of DP2 and CP1.

The most stable conformations of (a) **DP2** and (b) **CP1** from NMR analysis are shown. The intramolecular hydrogen bond that is discussed in the main text is highlighted by dotted blue lines. Shown in (b) is the most stable conformation from our NMR analysis (Figure 3a and Supplementary Figure 10–15), which is similar to the reported conformation in the previous report.[6]

a

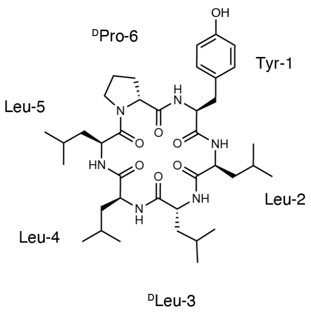

b

Name	substitution and position						Solubility (μM)	Conc. (μM)
	Tyr-1	Leu-2	DLeu-3	Leu-4	Leu-5	DPro-6		
**CP1**	NH	NH	NH	NH	NH	N	>100	100
**DP1**	O	NH	NH	NH	NH	N	25	25
**DP2**	NH	O	NH	NH	NH	N	56	55
**DP3**	NH	NH	O	NH	NH	N	56	55
**DP4**	NH	NH	NH	O	NH	N	5.5	5
**DP5**	NH	NH	NH	NH	O	N	5.2	5
**MP1**	NMe	NH	NH	NH	NH	N	67	65
**MP2**	NH	NMe	NH	NH	NH	N	91	90
**MP3**	NH	NH	NMe	NH	NH	N	82	80
**MP4**	NH	NH	NH	NMe	NH	N	42	40
**MP5**	NH	NH	NH	NH	NMe	N	4.1	4

c

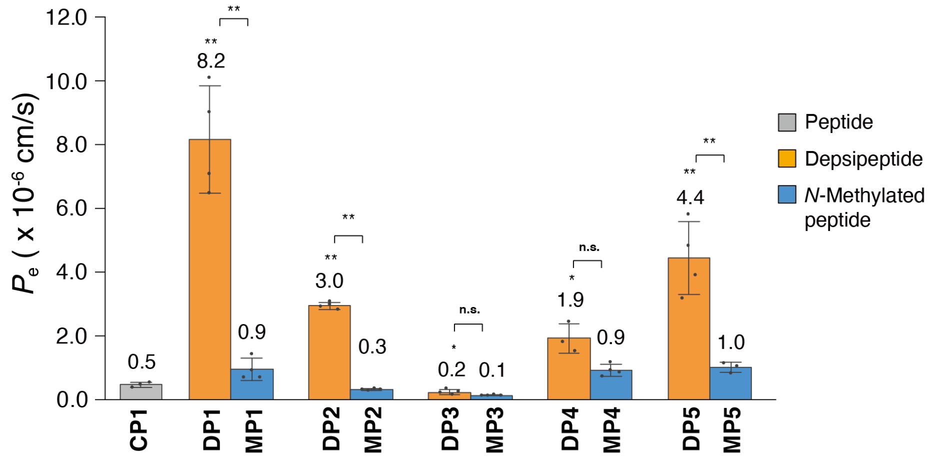

Supplementary Figure 8. Caco-2 assay with high concentrations.

(a) Chemical structure of **CP1**. (b) Sequences of synthesized and assayed compounds, solubility, and the concentration of compounds used in Caco-2 assay. The position of an amide-to-ester substitution and amide *N*-methylation is shown by O highlighted in orange and NMe highlighted in blue, respectively. In solubility assay, 2 μL of 10 mM DMSO stock was added to 165 μL of 0.1 M phosphate buffer (pH 7.4) and shaken at 37 °C for 4 h. After filtration, the concentration of compounds in the filtrate was measured by LC-UV (274 nm) to calculate solubility. (c) The permeability across cell monolayer of **CP1**, **DP1–5**, and **MP1–5**. The cell monolayer permeability assay was conducted using the same procedure with **Figure 2d** other than compound concentrations. Each bar represents the mean value, and the error bars the standard deviation from experiments carried out in triplicate (**CP1**, **DP4**, and **MP5**) or quadruplicate (other than **CP1**, **DP4**, and **MP5**). P values were determined by a two-sided Welch's *t*-test. ** $p < 0.01$, * $p < 0.05$. n.s. denotes not significant. p (**CP1** vs. **DP1**) = 0.0027, p (**DP1** vs. **MP1**) = 0.0026, p (**CP1** vs. **DP2**) < 0.0001, p (**DP2** vs. **MP2**) < 0.0001, p (**CP1** vs. **DP3**) = 0.0114, p (**DP3** vs. **MP3**) = 0.0729, p (**CP1** vs. **DP4**) = 0.0292, p (**DP4** vs. **MP4**) = 0.0514, p (**CP1** vs. **DP5**) = 0.0060 and p (**DP5** vs. **MP5**) = 0.0083.

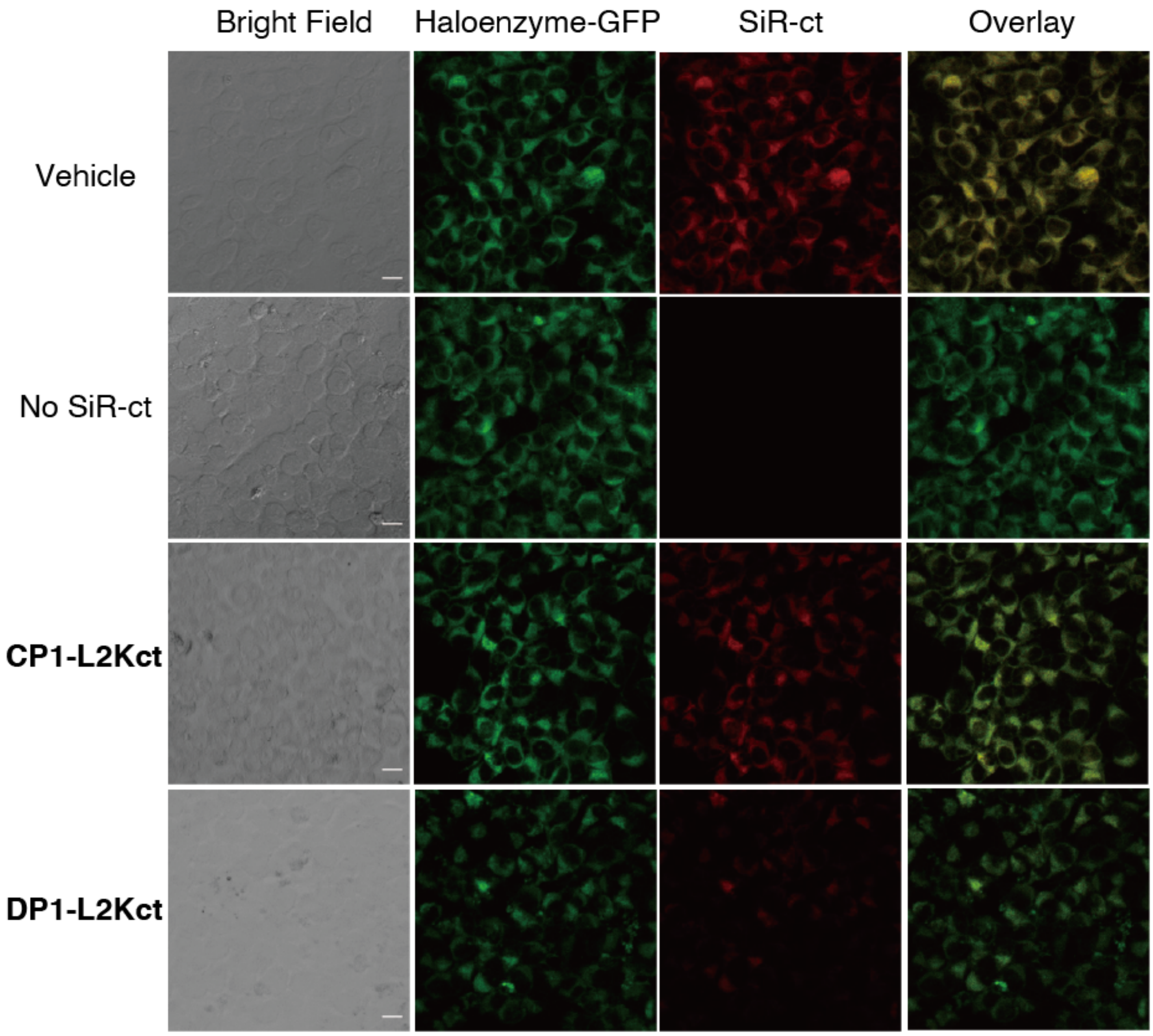

Supplementary Figure 9. Confocal microscope images of cells in CAPA.

The cells were treated with 5 μM peptide solution. Green fluorescence represents a fusion protein of GFP and HaloTag, and red fluorescence represents SiR-ct dye. The number of cells spread in one chamber was increased from 5×10^4 cells (Figure 2g) to 2×10^5 cells. Other experimental conditions are the same with those of Figure 2g. A scale bar (20 μm) is included in the bright field image of each dataset.

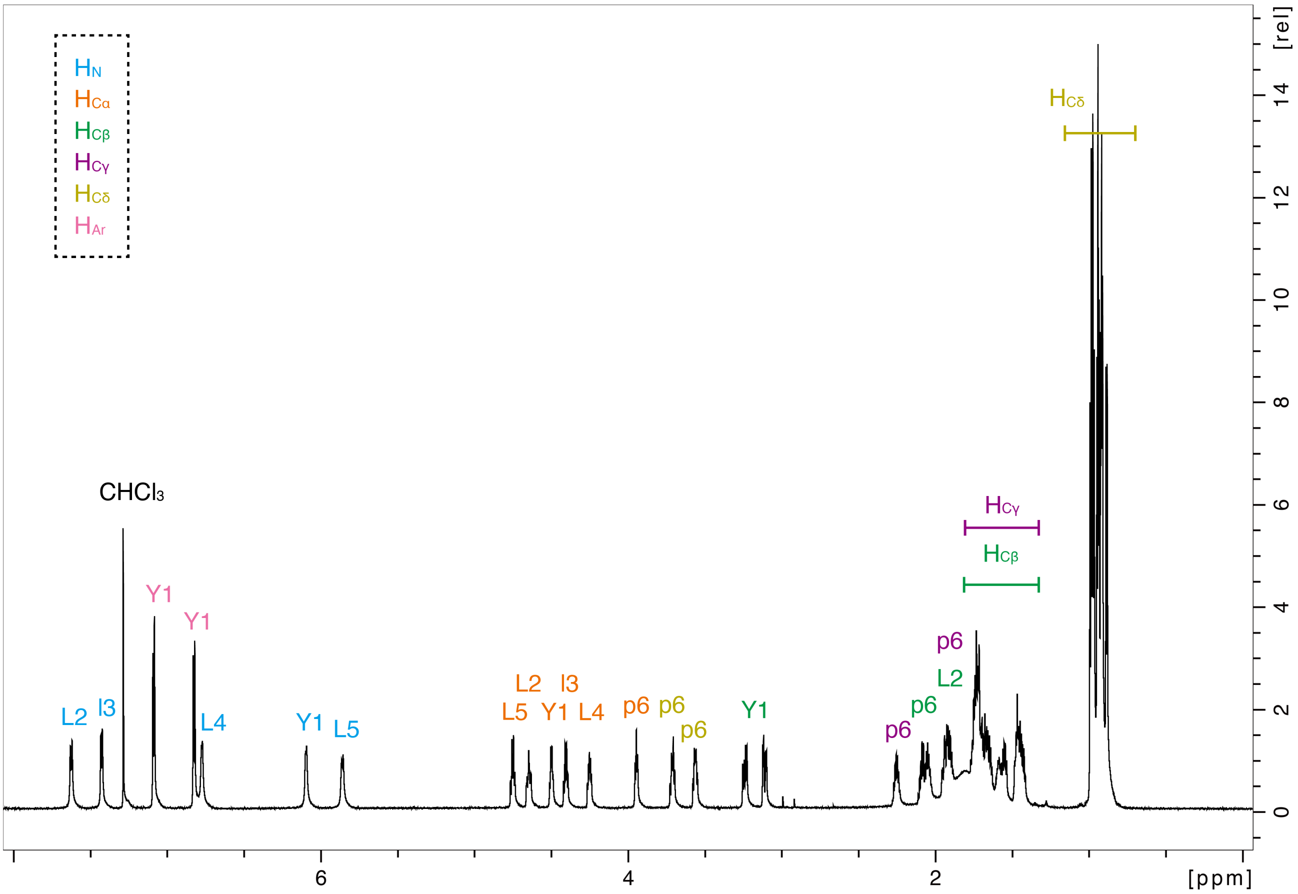

Supplementary Figure 10. ^{1}H-NMR spectrum of CP1 for conformational determination

The ^{1}H-NMR spectrum of **CP1** was recorded in $CDCl_3$. For the assignment of each peak, COSY, TOCSY, HSQC, HMBC, and ROESY spectra were recorded. Light blue, orange, green, purple, dark yellow, and pink letters denote peaks derived from H_N, $H_{C\alpha}$, $H_{C\beta}$, $H_{C\delta}$, $H_{C\gamma}$, and H_{aryl}, respectively. The one-letter residue code and the number above a peak indicate the residue number to which the proton belongs.

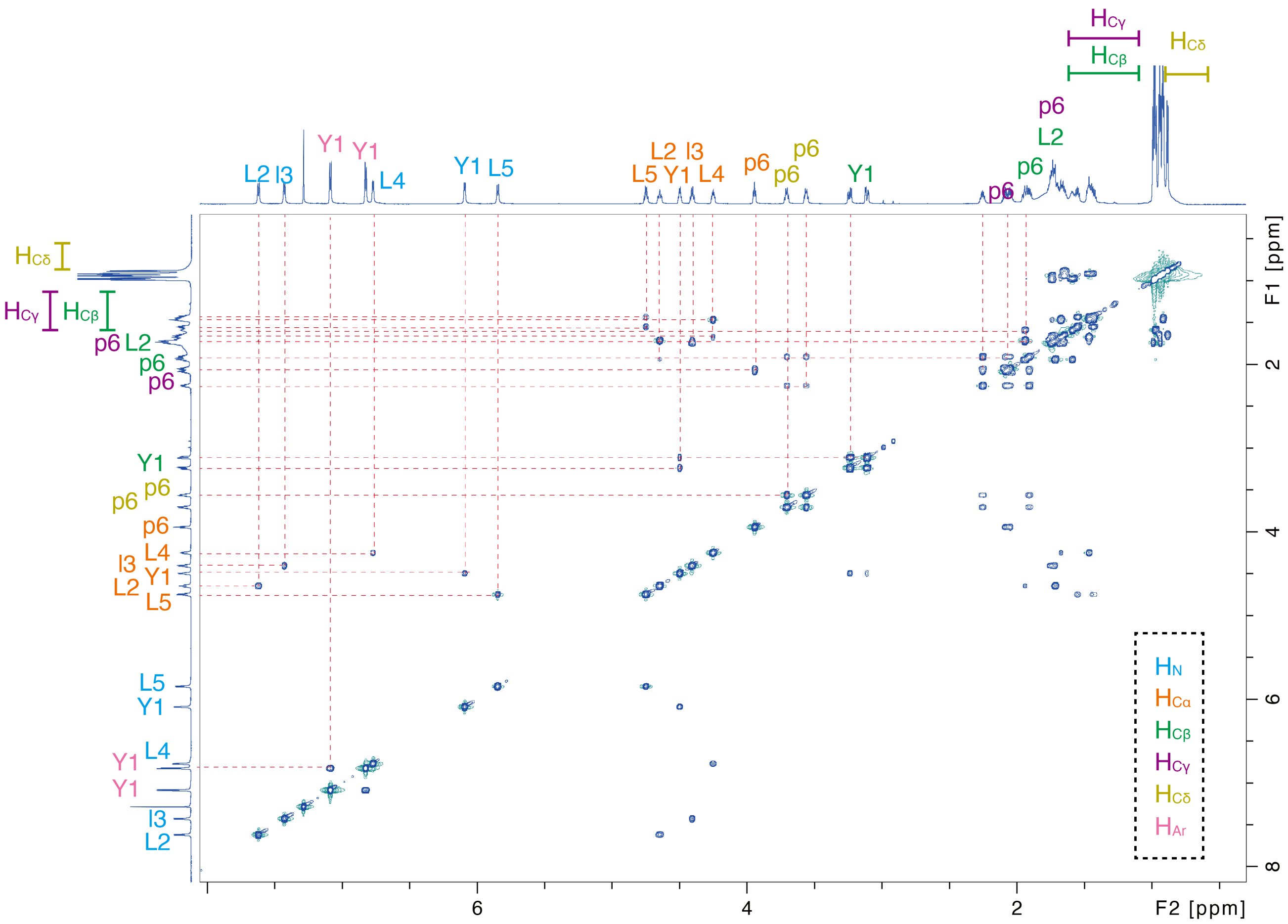

Supplementary Figure 11. COSY spectrum of CP1 for conformational determination

The COSY-NMR spectrum of **CP1** was recorded in $CDCl_3$. Correlation peaks that support the assignment of ^{1}H-NMR are shown with pink dashed lines.

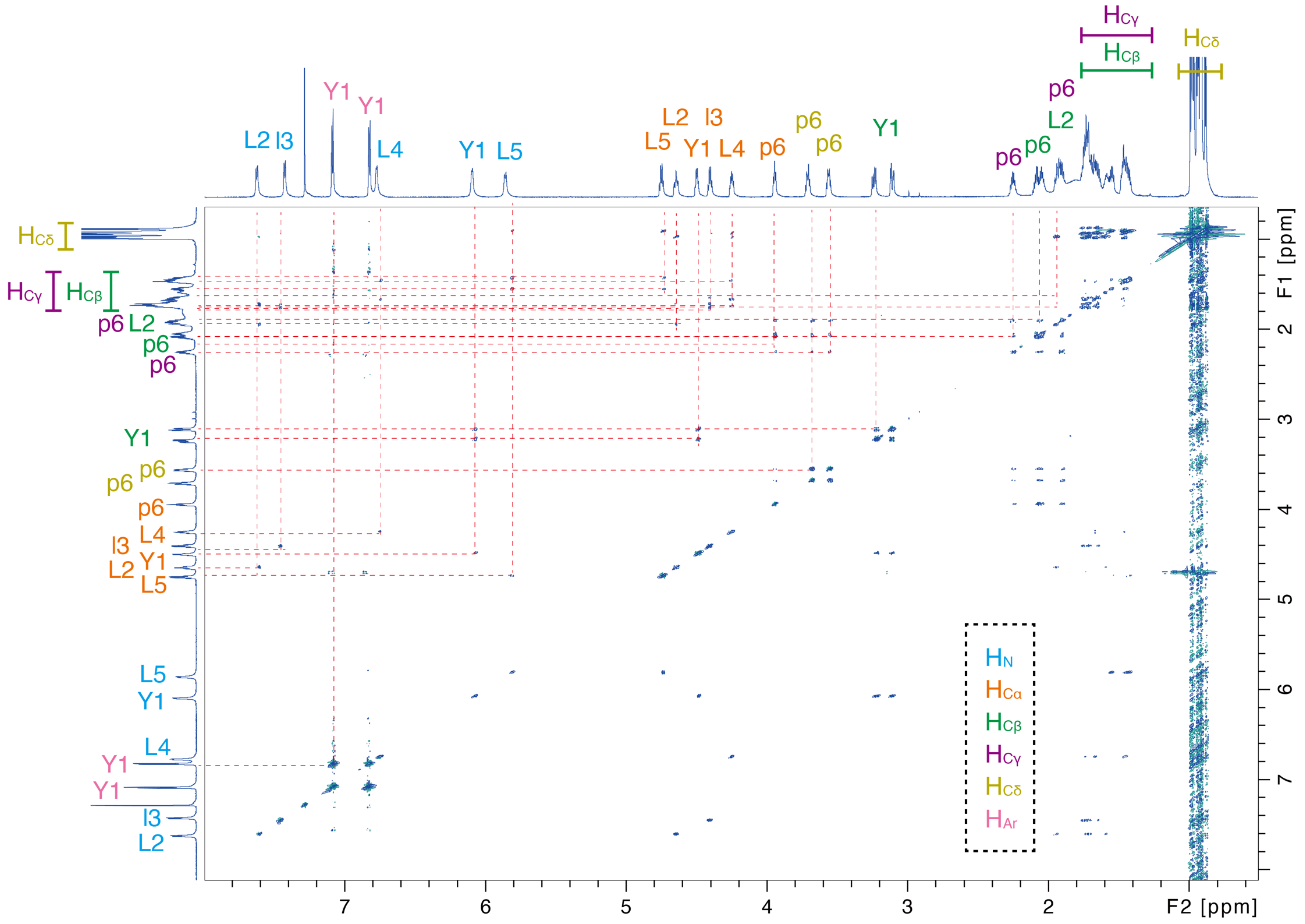

Supplementary Figure 12. TOCSY spectrum of CP1 for conformational determination

The TOCSY-NMR spectrum of **CP1** was recorded in $CDCl_3$. Correlation peaks that support the assignment of ^{1}H-NMR are shown with pink dashed lines.

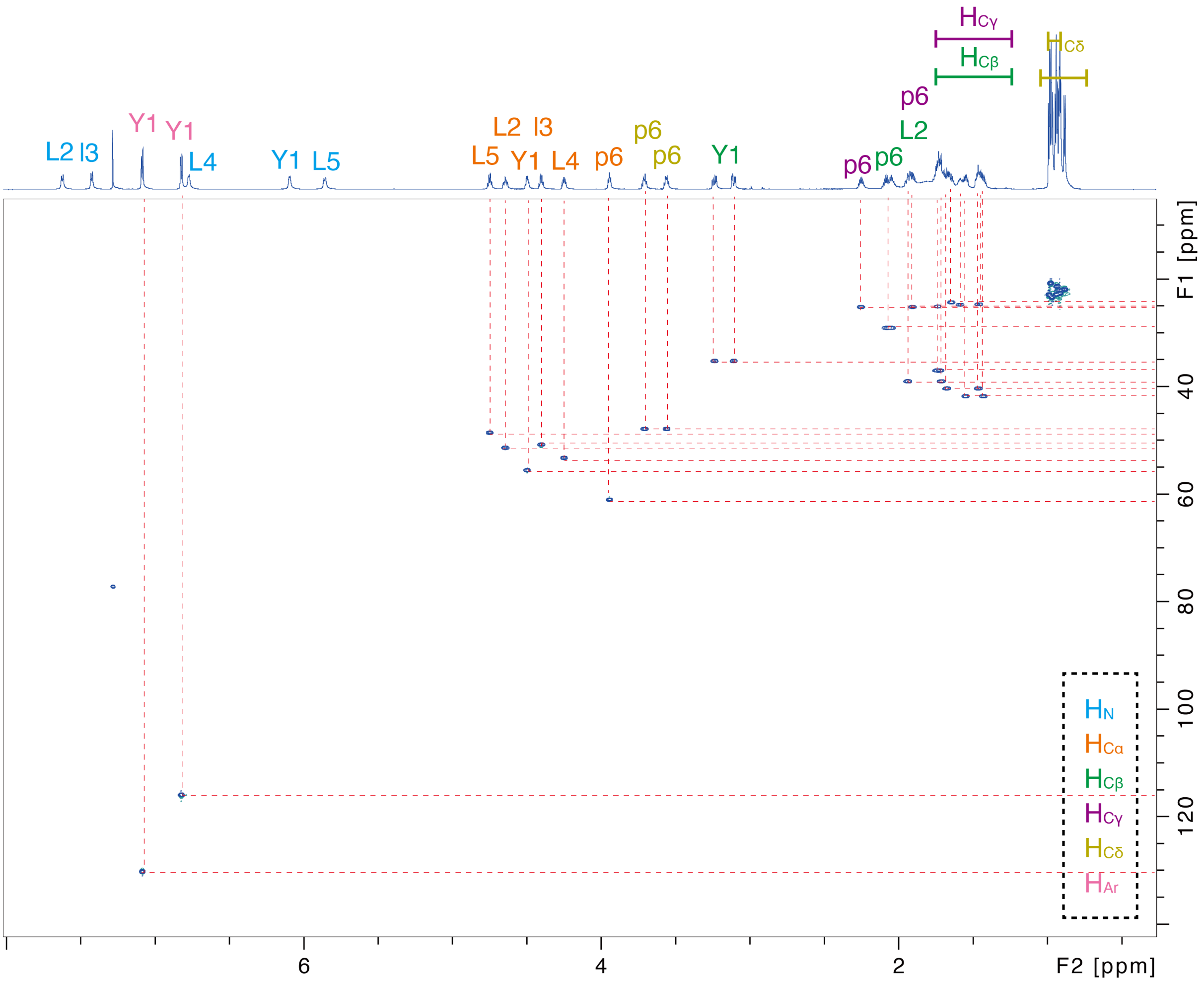

Supplementary Figure 13. HSQC spectrum of CP1 for conformational determination

The HSQC-NMR spectrum of **CP1** was recorded in $CDCl_3$. Correlation peaks that support the assignment of ^{1}H-NMR are shown with pink dashed lines.

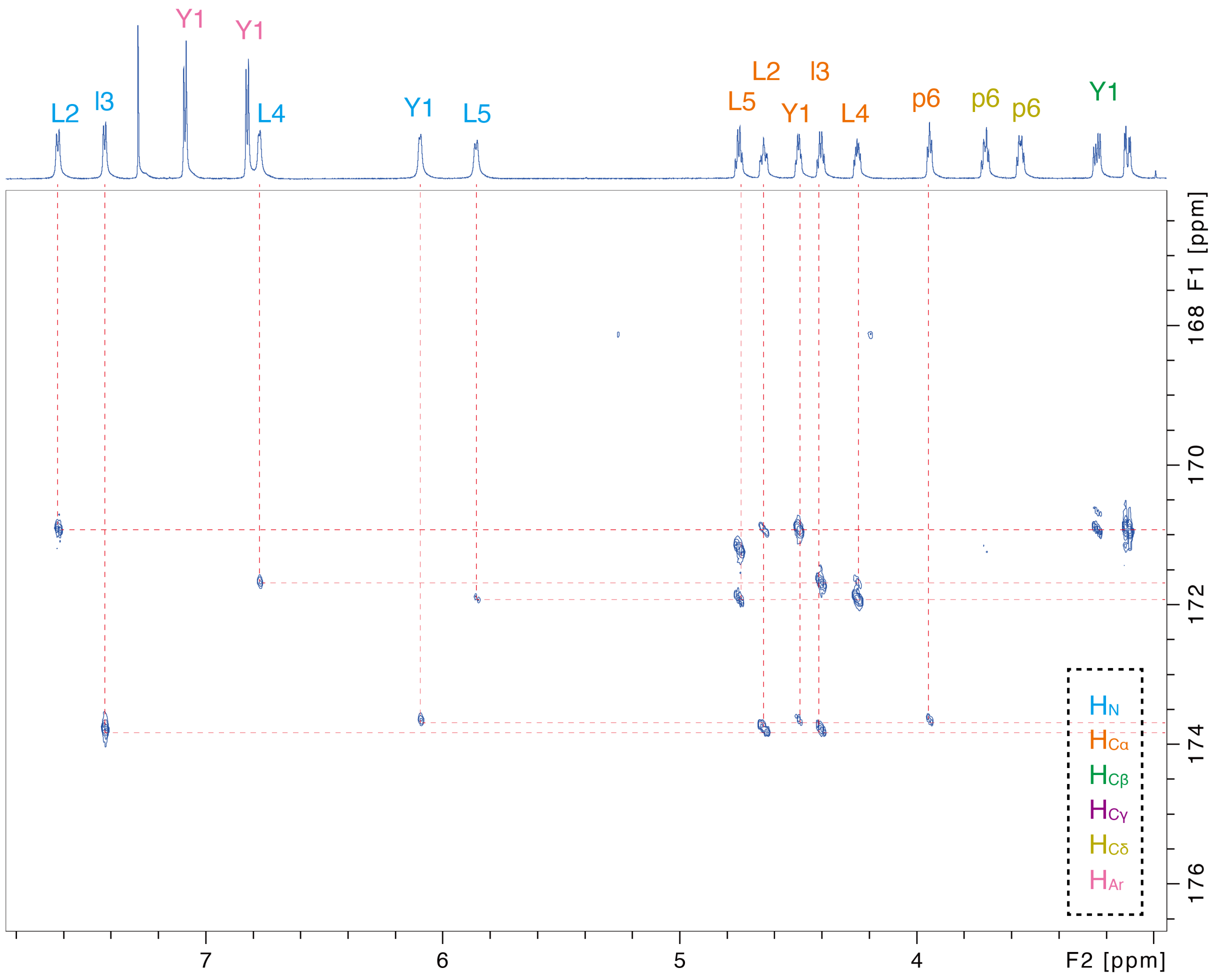

Supplementary Figure 14. HMBC spectrum of CP1 for conformational determination

The HMBC-NMR spectrum of **CP1** was recorded in $CDCl_3$. Correlation peaks that support the assignment of ^{1}H-NMR are shown with pink dashed lines.

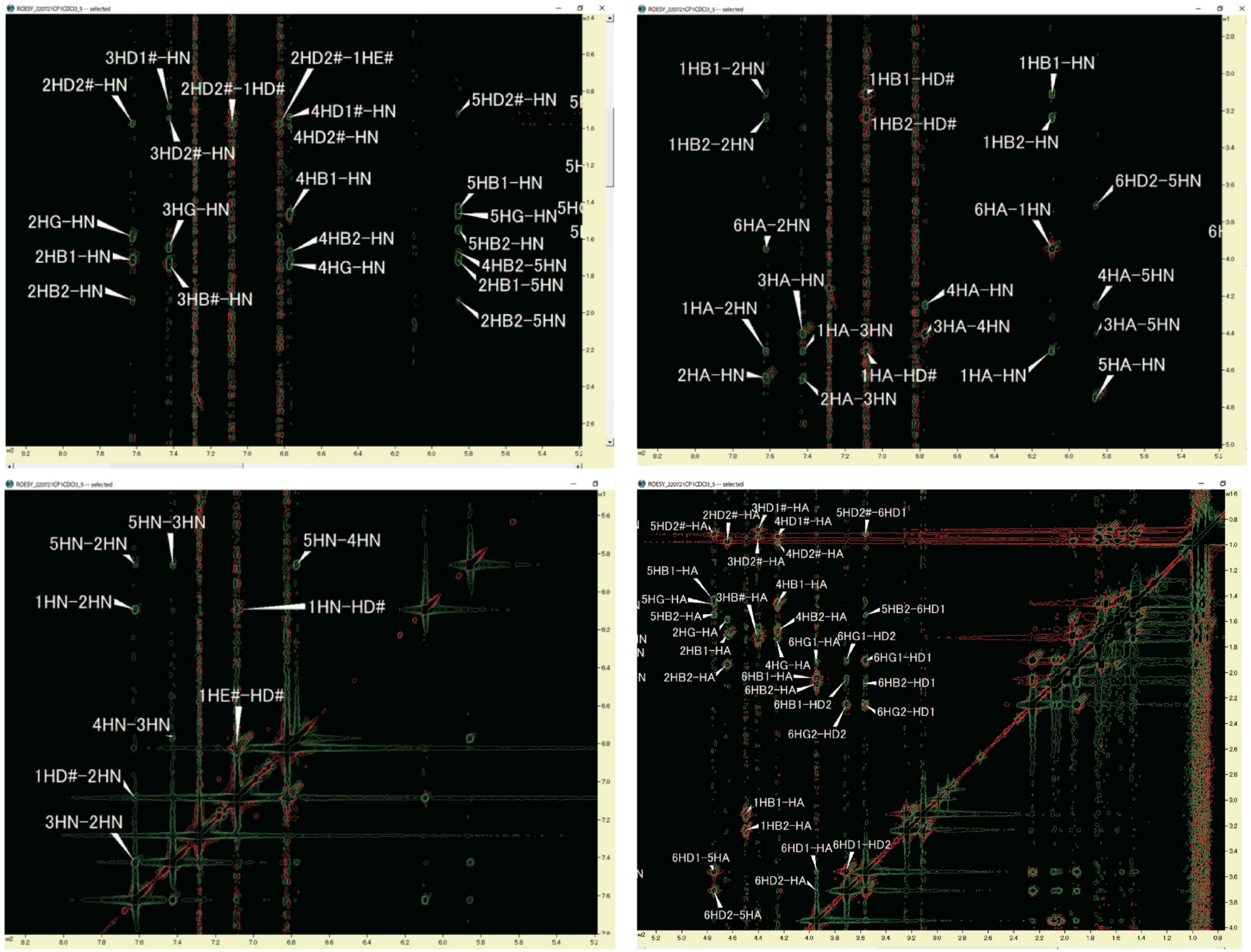

Supplementary Figure 15. ROESY spectrum of CP1 for conformational determination

The ROESY-NMR spectrum of **CP1** was recorded in $CDCl_3$. Correlation peaks indicating the proximities of protons are indicated with the names of the proximal two protons. The correlation peaks are summarized in a Supplementary Table.

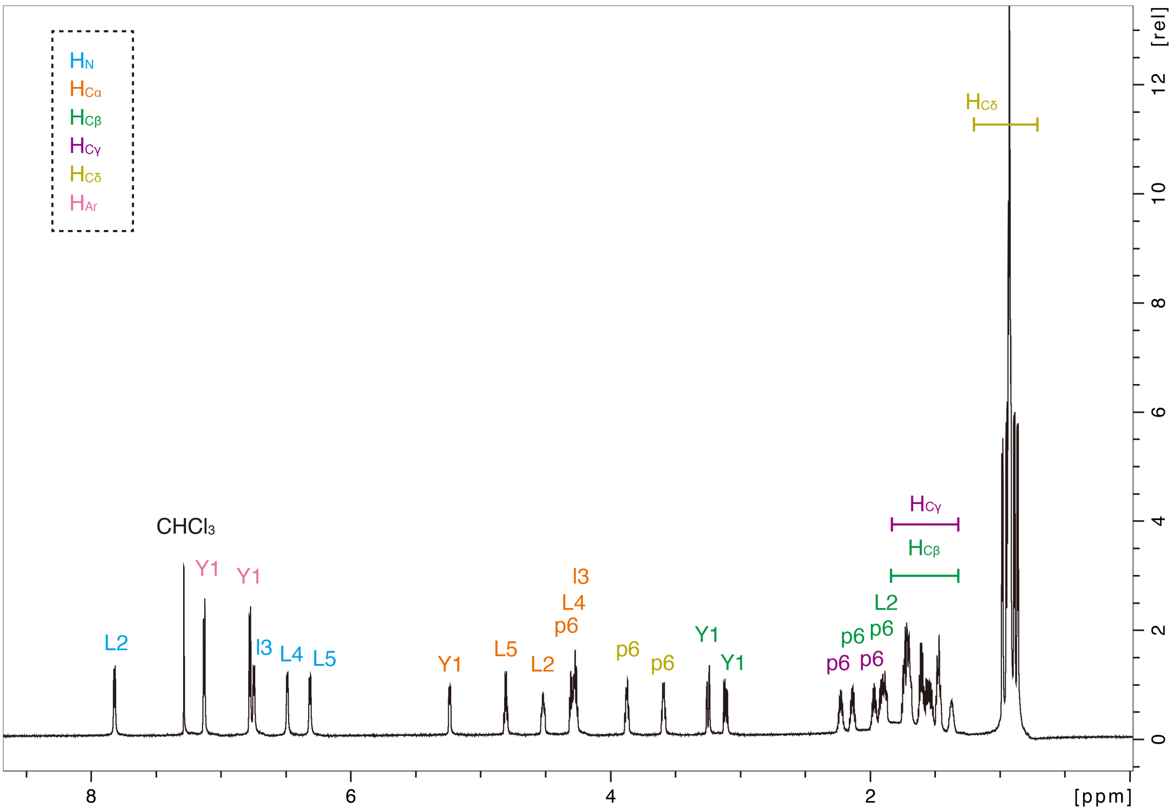

Supplementary Figure 16. ¹H-NMR of DP1 for conformational determination

The ^{1}H-NMR spectrum of **DP1** was recorded in $CDCl_3$. For the assignment of each peak, COSY, TOCSY, HSQC, HMBC, and ROESY spectra were recorded. Light blue, orange, green, purple, dark yellow, and pink letters denote peaks derived from H_N, $H_{C\alpha}$, $H_{C\beta}$, $H_{C\gamma}$, $H_{C\delta}$, and H_{aryl}, respectively. The one-letter residue code and the number above a peak indicate the residue number to which the proton belongs. $H_{C\beta}$, $H_{C\delta}$, and $H_{C\gamma}$ were also assigned, but not all the assignment was shown because of their severe overlaps.

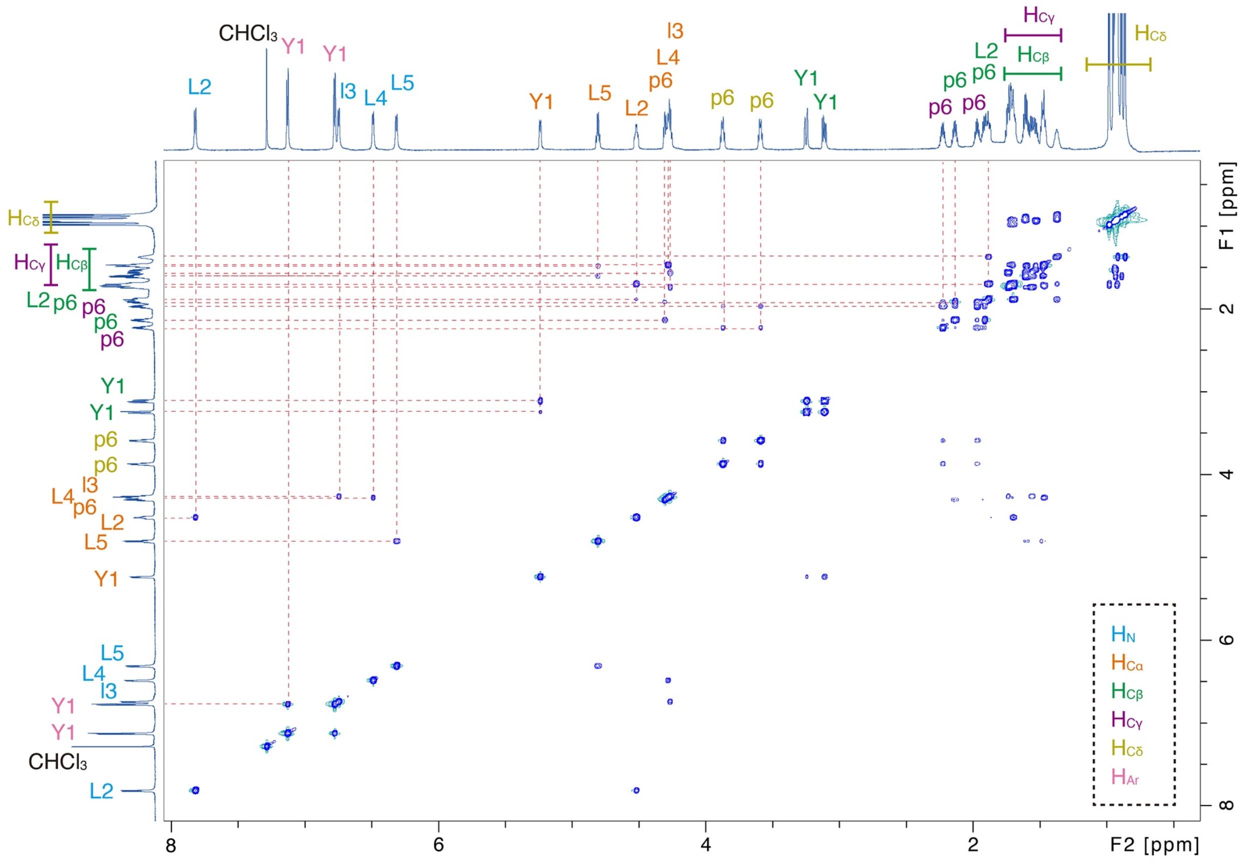

Supplementary Figure 17. COSY spectrum of DP1 for conformational determination

The COSY-NMR spectrum of **DP1** was recorded in $CDCl_3$. Correlation peaks that support the assignment of ^{1}H-NMR are shown with pink dashed lines.

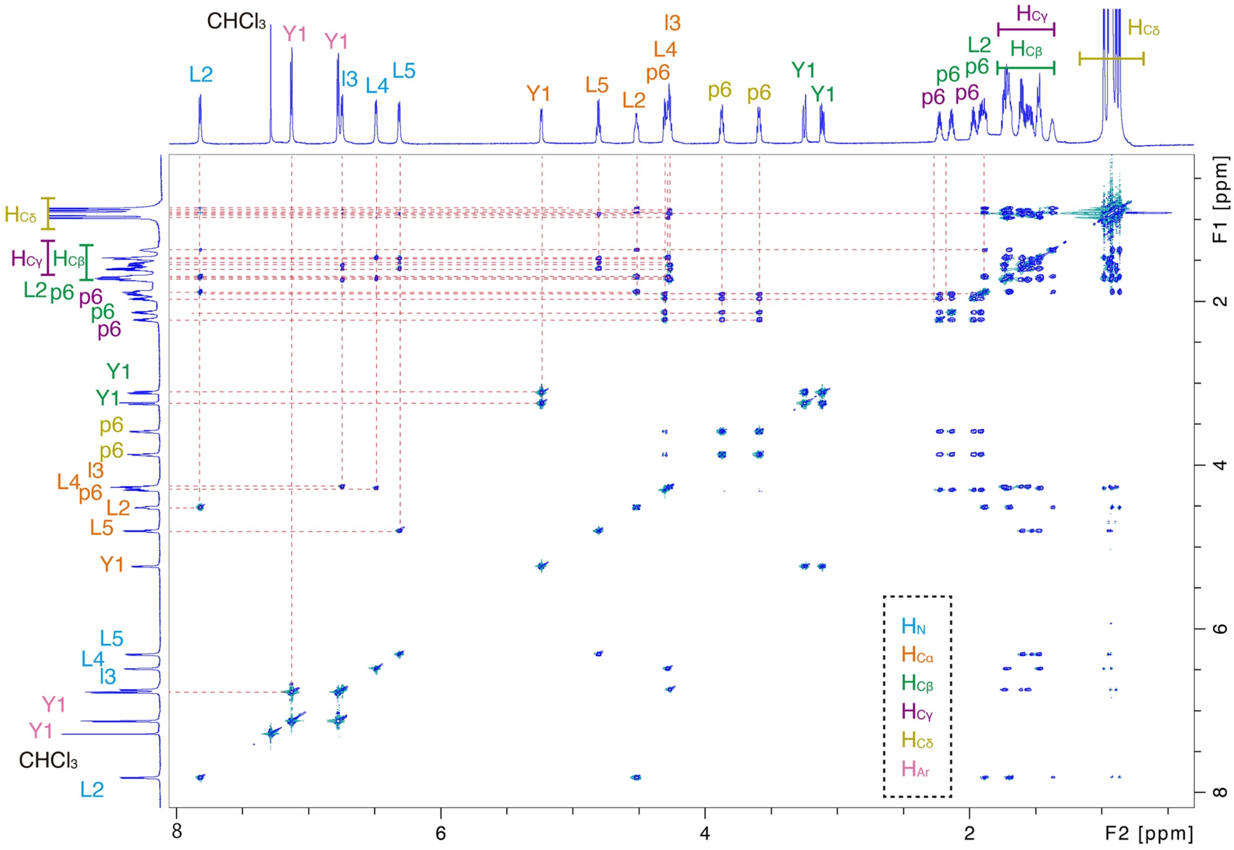

Supplementary Figure 18. TOCSY spectrum of DP1 for conformational determination

The TOCSY-NMR spectrum of **DP1** was recorded in $CDCl_3$. Correlation peaks that support the assignment of ^{1}H-NMR are shown with pink dashed lines.

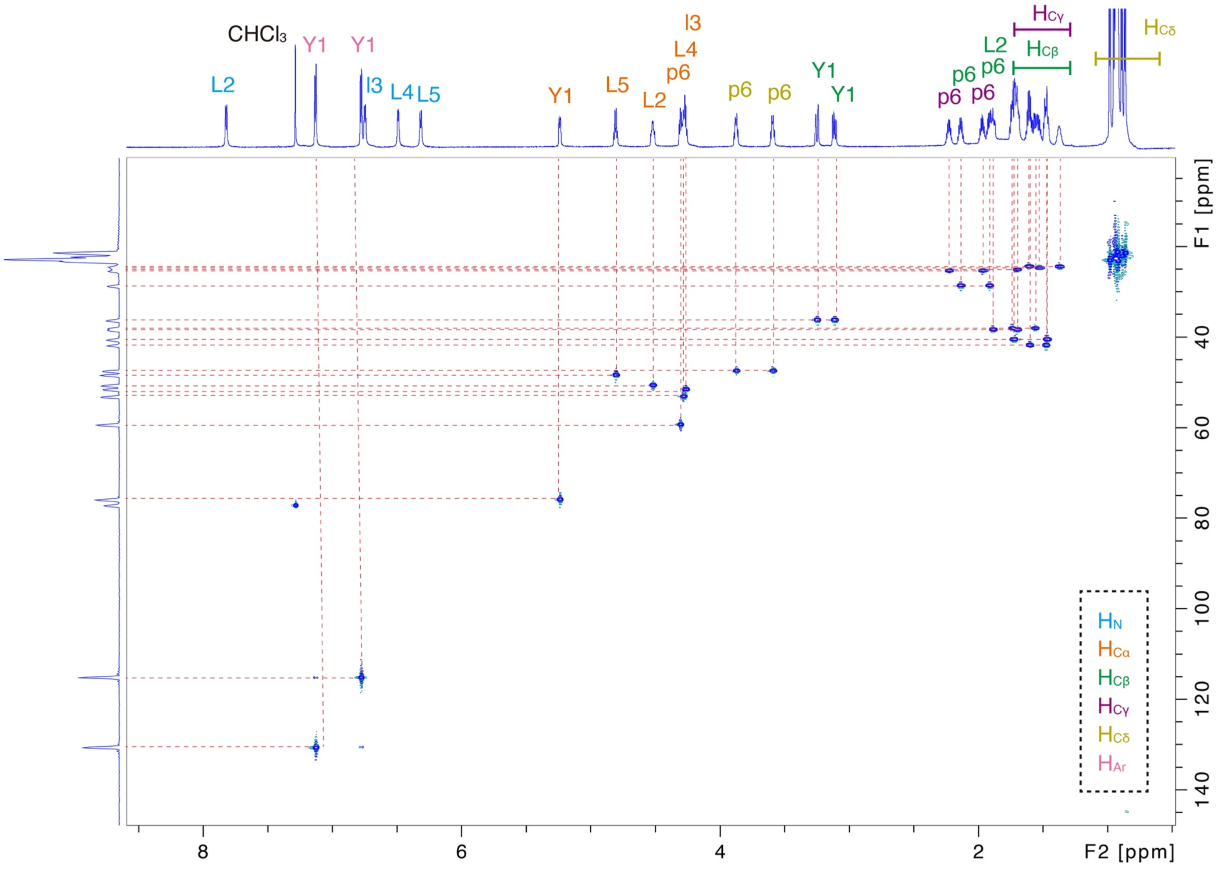

Supplementary Figure 19. HSQC spectrum of DP1 for conformational determination

The HSQC-NMR spectrum of **DP1** was recorded in $CDCl_3$. Correlation peaks that support the assignment of ^{1}H-NMR are shown with pink dashed lines.

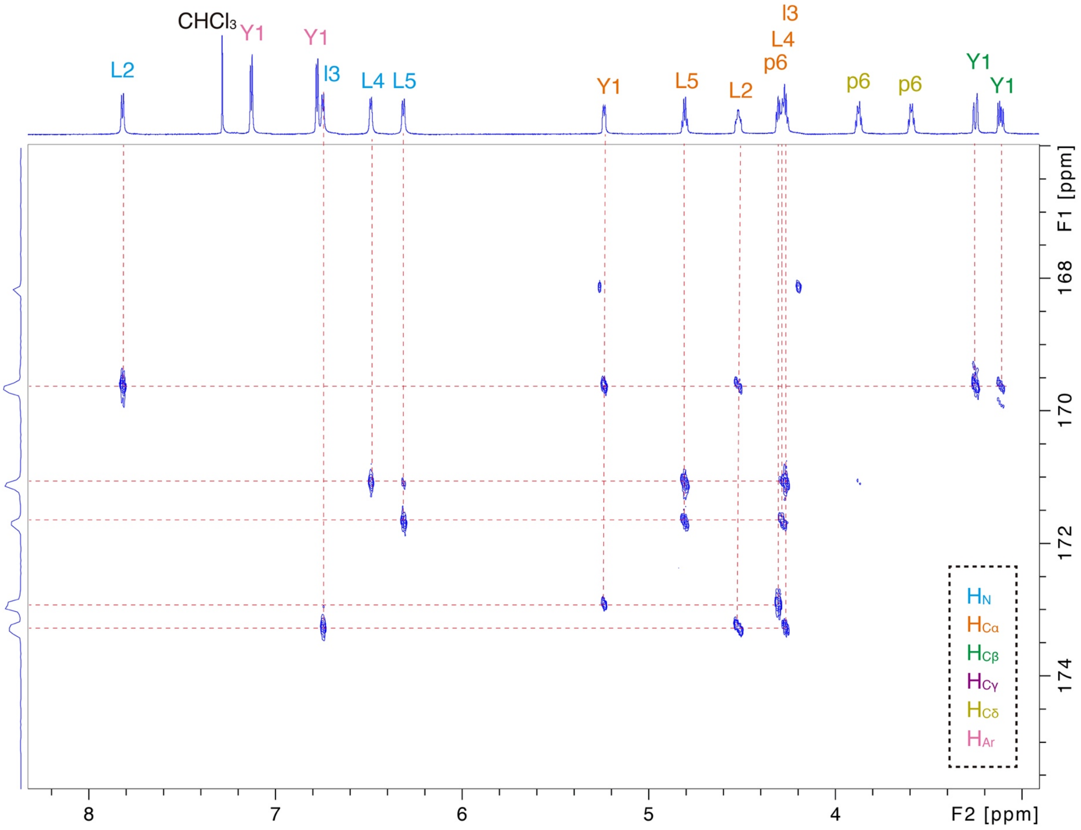

Supplementary Figure 20. HMBC spectrum of DP1 for conformational determination

The HMBC-NMR spectrum of **DP1** was recorded in $CDCl_3$. The magnified spectrum with the correlation area of carbonyl carbon, H_N, and $H_{C\alpha}$ was shown. Correlation peaks that support the assignment of ^{1}H-NMR are shown with pink dashed lines.

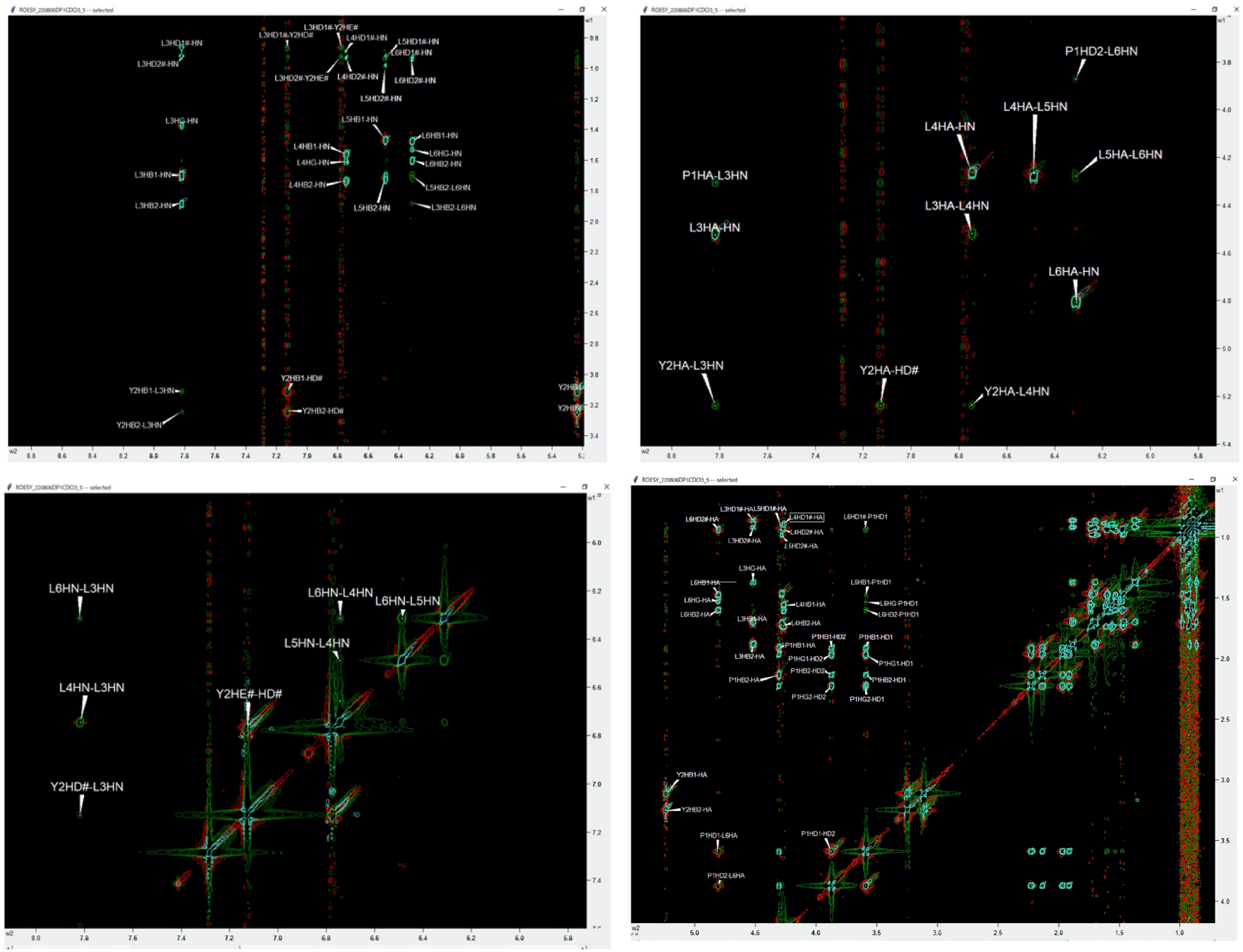

Supplementary Figure 21. ROESY spectrum of DP1 for conformational determination

The ROESY-NMR spectrum of **DP1** was recorded in $CDCl_3$ Correlation peaks indicating the proximities of protons are indicated with the names of the proximal two protons. The correlation peaks are summarized in a Supplementary Table.

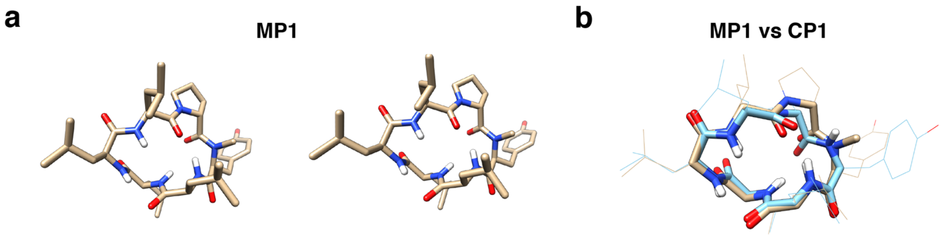

Supplementary Figure 22. NMR solution structure of MP1.

(a) Stereoviews of the NMR solution structure of **MP1** in $CDCl_3$. (b) The superposition of **MP1** with **CP1**. **MP1** is shown in brown and **CP1** is shown in blue.

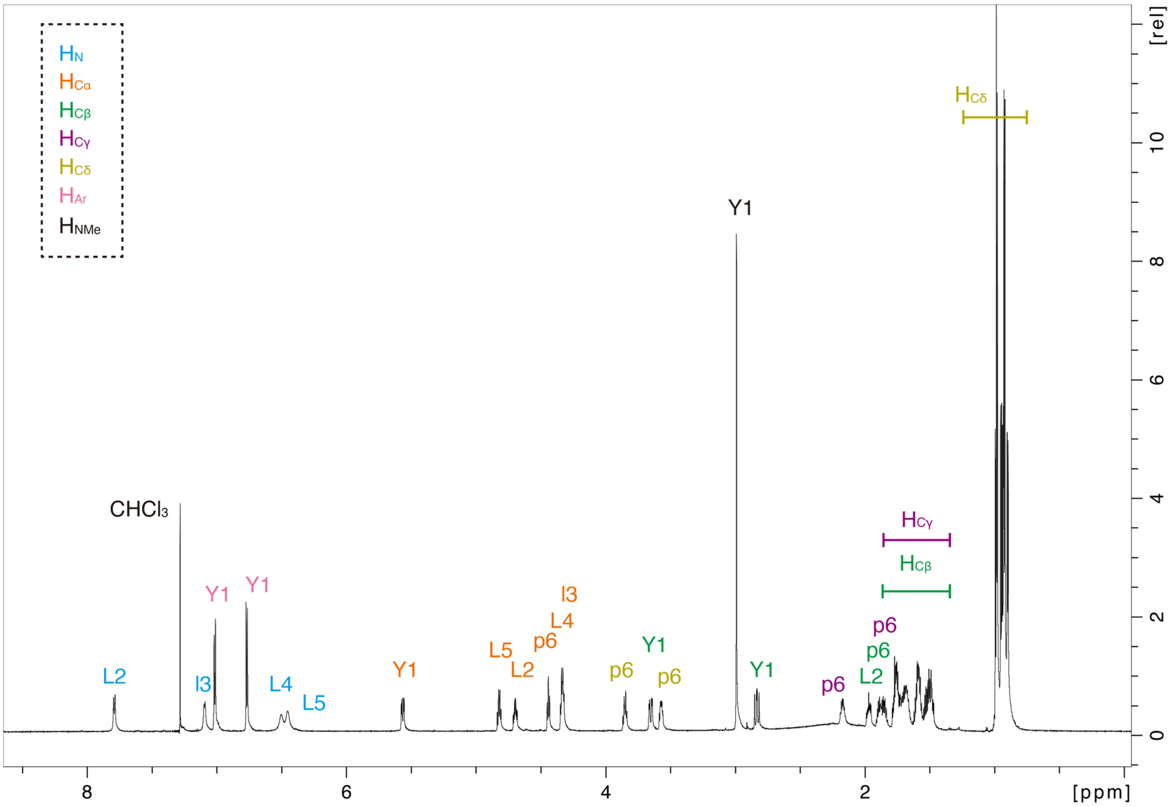

Supplementary Figure 23. ^{1}H-NMR of MP1 for conformational determination

The ^{1}H-NMR spectrum of **MP1** was recorded in $CDCl_3$. For the assignment of each peak, COSY, TOCSY, HSQC, HMBC, and ROESY spectra were recorded. Light blue, orange, green, purple, dark yellow, pink, and black letters denote peaks derived from H_N, $H_{C\alpha}$, $H_{C\beta}$, $H_{C\gamma}$, $H_{C\delta}$, H_{aryl}, and *N*-methyl protons, respectively. The one-letter residue code and the number above a peak indicate the residue number to which the proton belongs. $H_{C\beta}$, $H_{C\delta}$, and $H_{C\gamma}$, were also assigned, but not all the assignment was shown because of their severe overlaps.

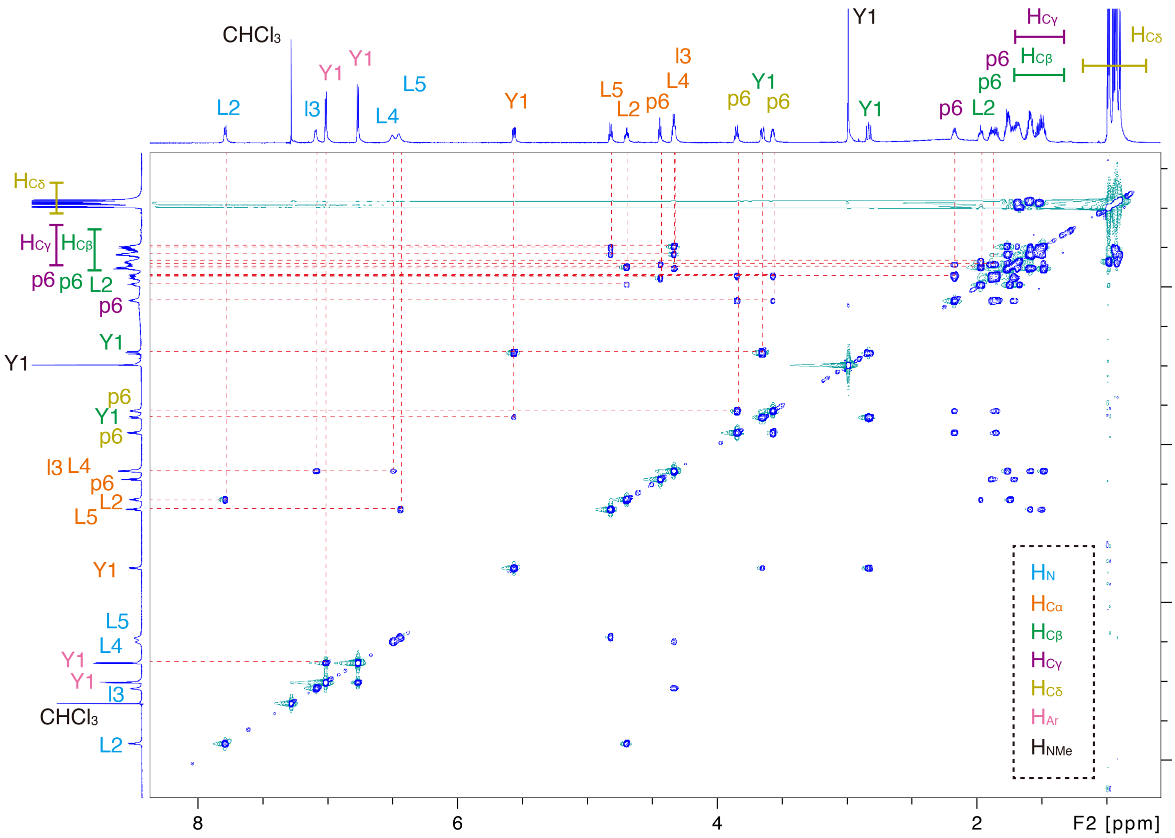

Supplementary Figure 24. COSY spectrum of MP1 for conformational determination

The COSY-NMR spectrum of **MP1** was recorded in $CDCl_3$. Correlation peaks that support the assignment of ^{1}H-NMR are shown with pink dashed lines.

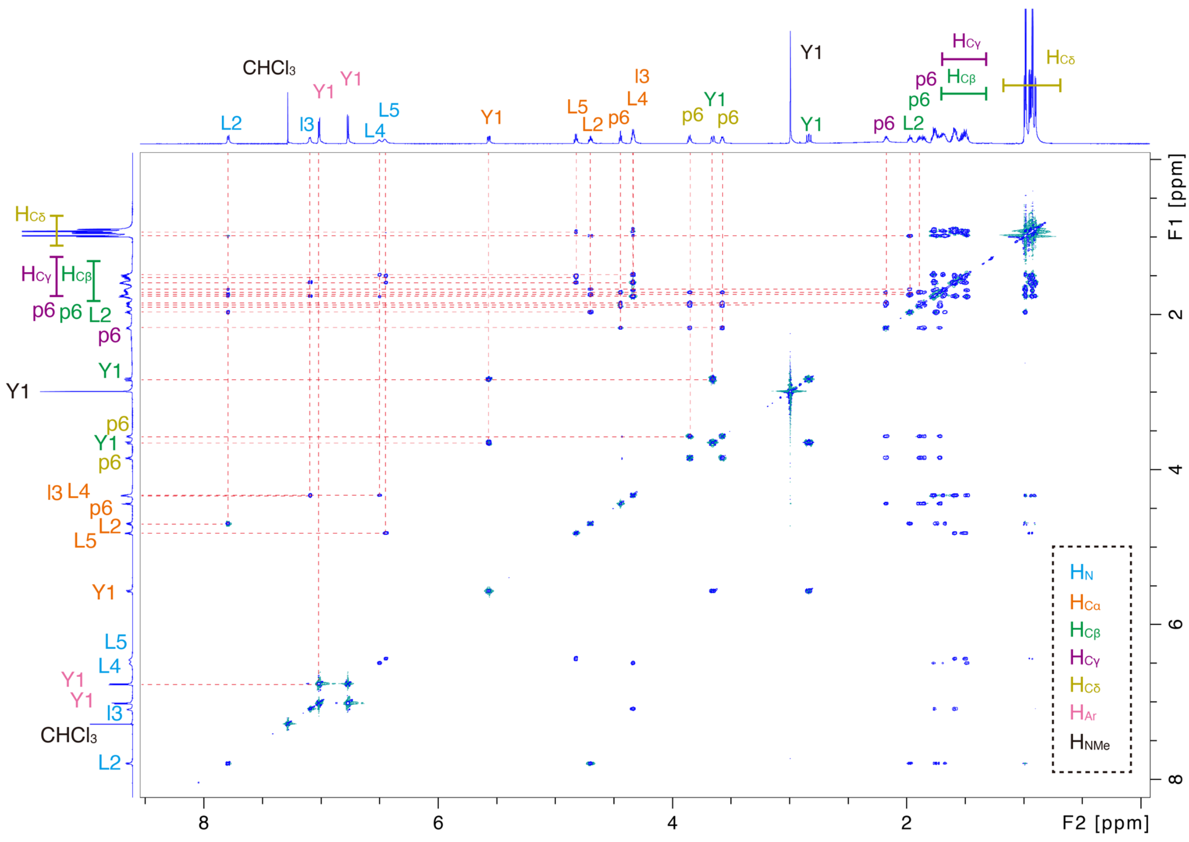

Supplementary Figure 25. TOCSY spectrum of MP1 for conformational determination

The TOCSY-NMR spectrum of **MP1** was recorded in $CDCl_3$. Correlation peaks that support the assignment of ^{1}H-NMR are shown with pink dashed lines.

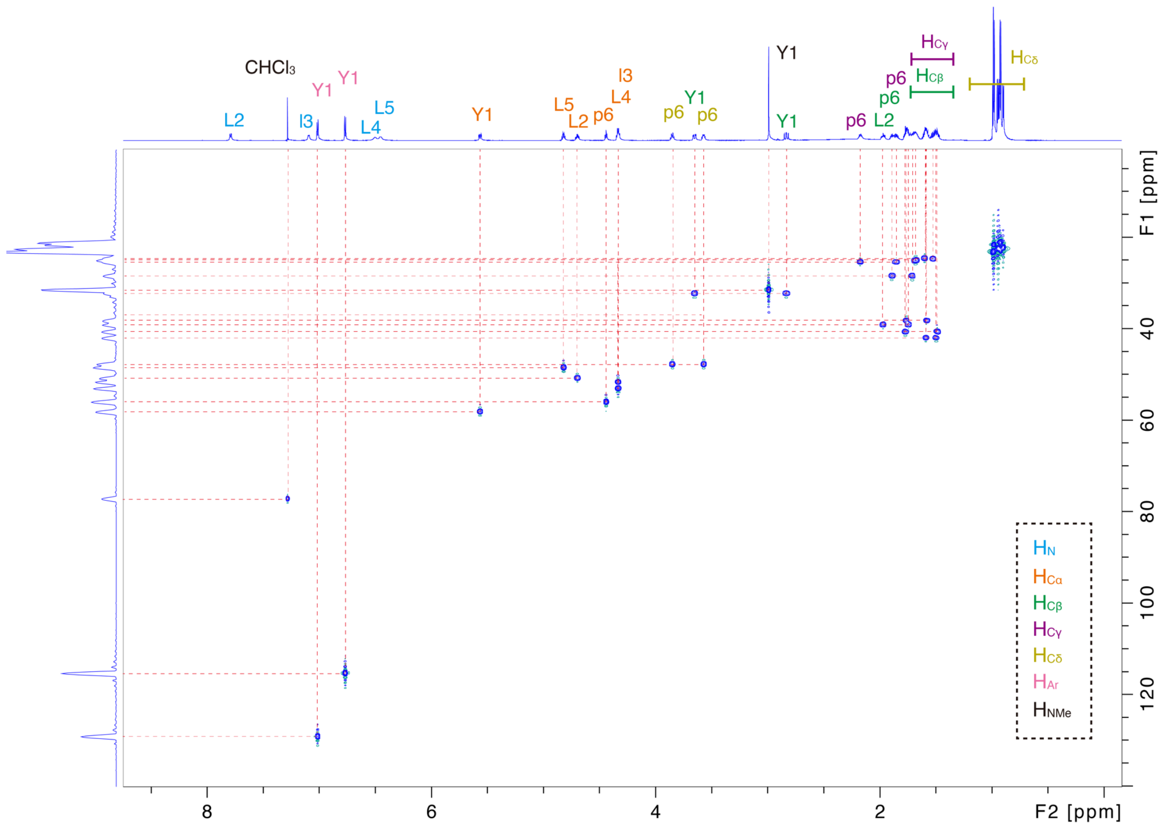

Supplementary Figure 26. HSQC spectrum of MP1 for conformational determination

The HSQC-NMR spectrum of **MP1** was recorded in $CDCl_3$. Correlation peaks that support the assignment of ^{1}H-NMR are shown with pink dashed lines.

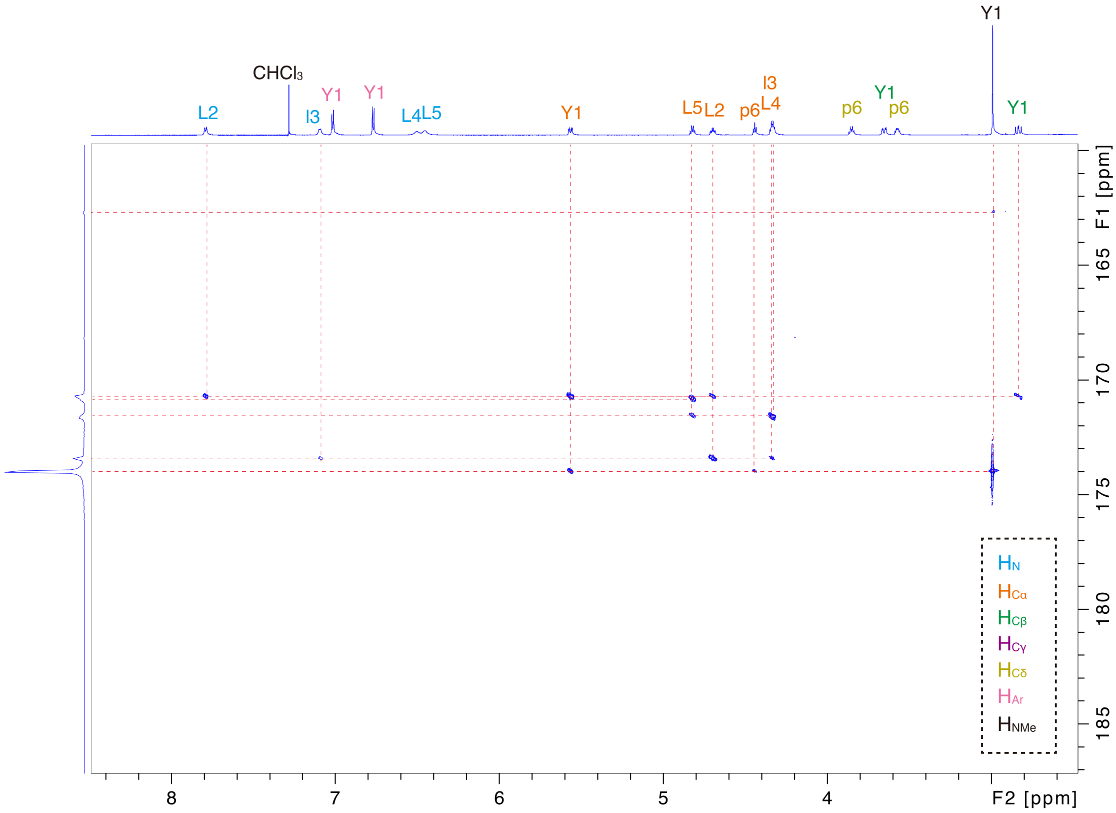

Supplementary Figure 27. HMBC spectrum of MP1 for conformational determination

The HMBC-NMR spectrum of **MP1** was recorded in $CDCl_3$. Correlation peaks that support the assignment of ^{1}H-NMR are shown with pink dashed lines.

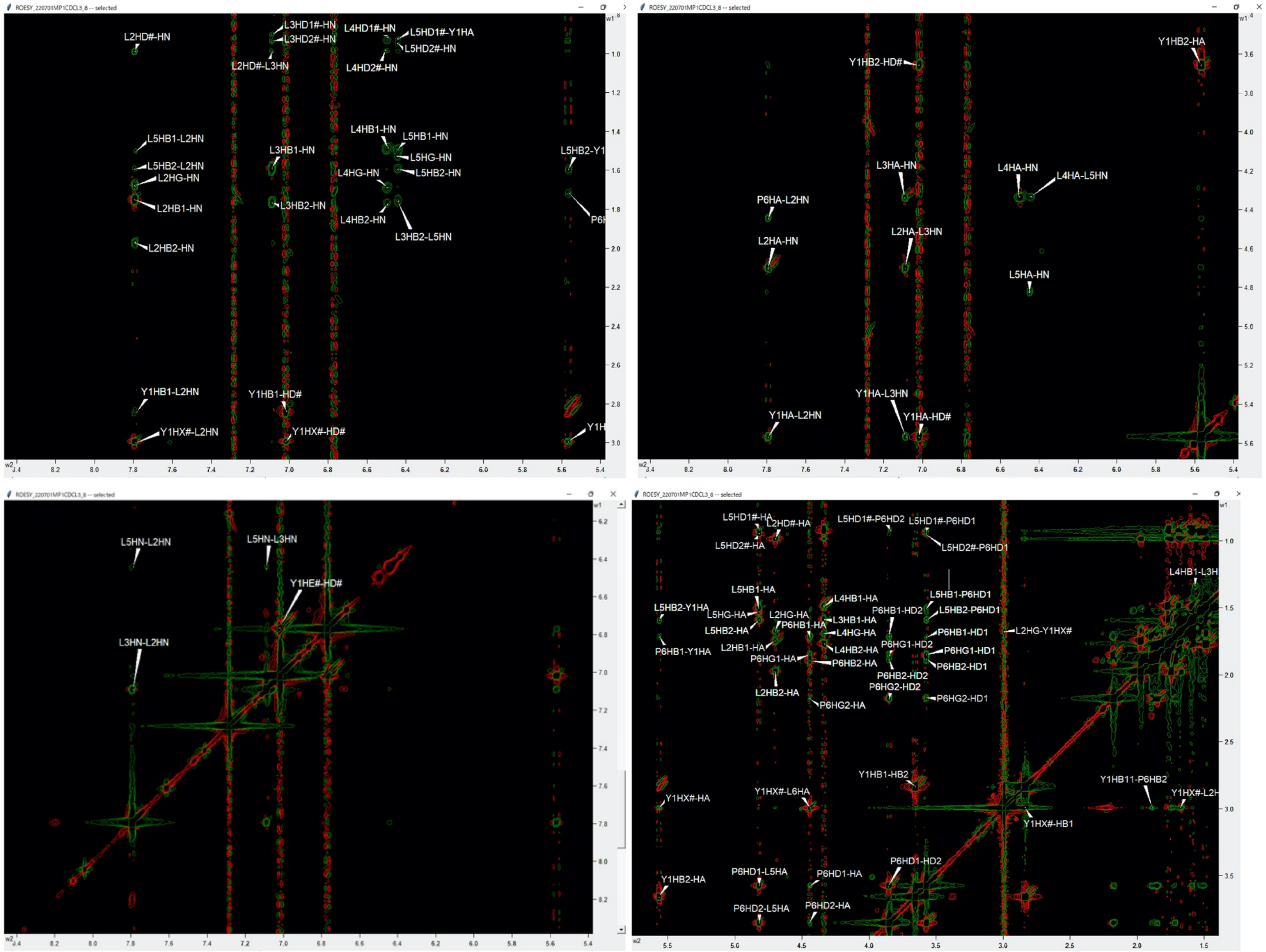

Supplementary Figure 28. ROESY spectrum of MP1 for conformational determination

The ROESY-NMR spectrum of **MP1** was recorded in $CDCl_3$. Correlation peaks indicating the proximities of protons are indicated with the names of the proximal two protons. The correlation peaks are summarized in a Supplementary Table.

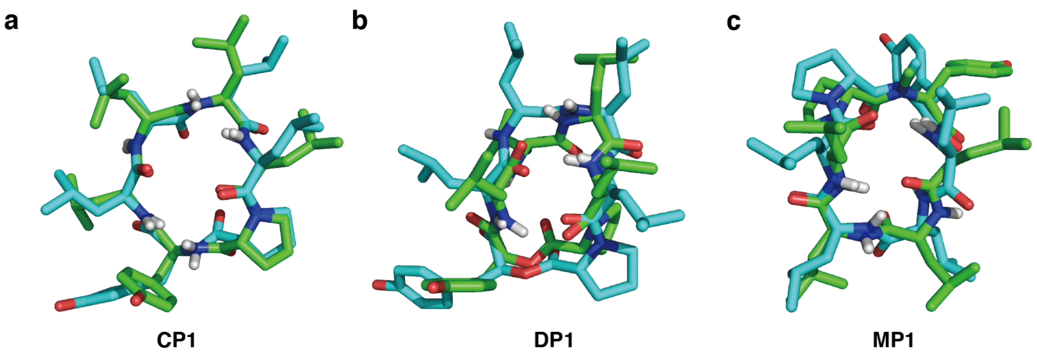

Supplementary Figure 29. Comparison of the representative conformations in high dielectric solvents from NMR and enhanced sampling MD simulations.

The overlay of the representative conformations of (a) **CP1**, (b) **DP1**, (c) **MP1** from NMR in 1:1 mixture of DMSO and water (green) and simulations in water (cyan). Two structures are overlayed using backbone atoms (N, C_α, C', and O).

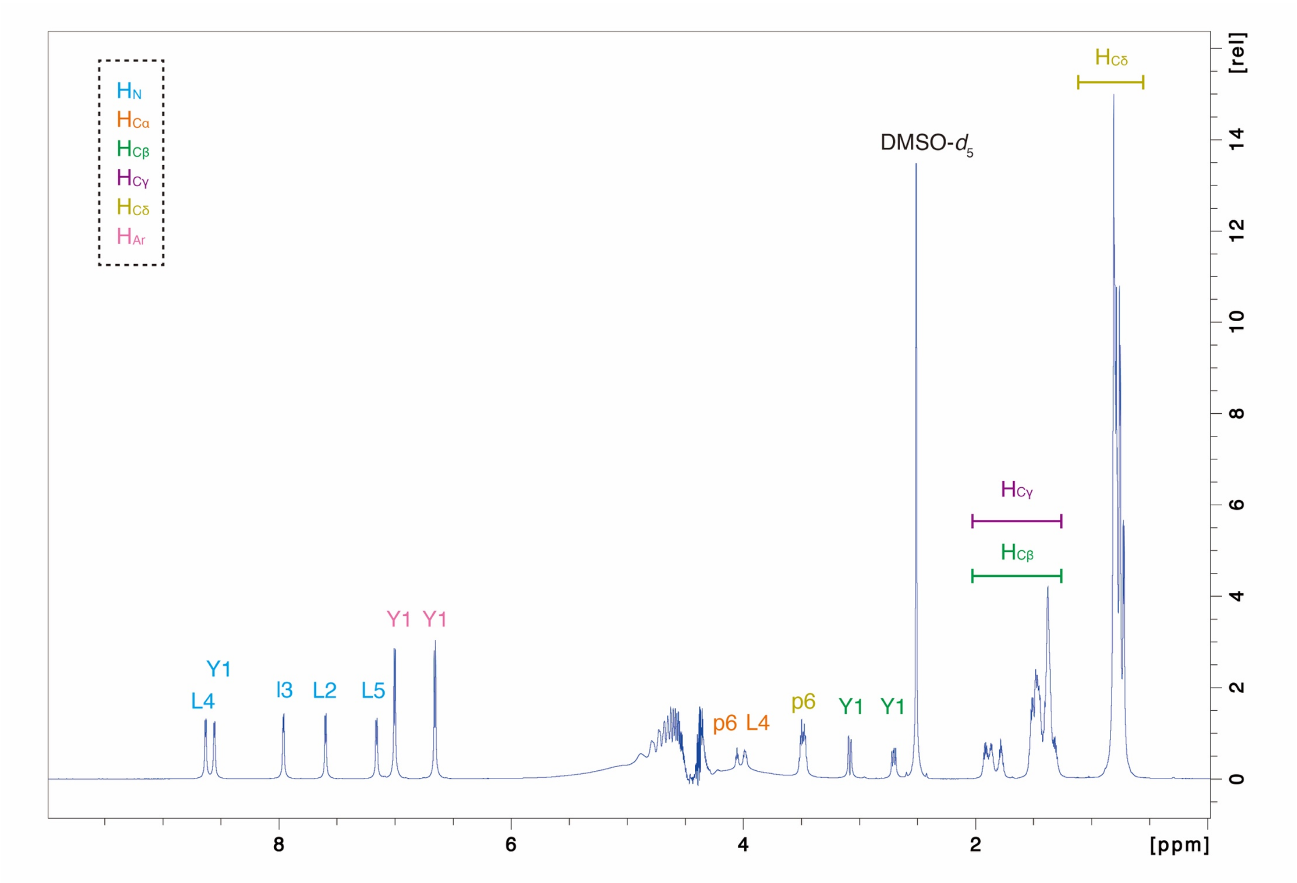

Supplementary Figure 30. ^{1}H-NMR of CP1 in 50% DMSO/H_2O

The ^{1}H-NMR spectrum of **CP1** was recorded in 50% DMSO/H_2O. For the assignment of each peak, COSY, TOCSY, HSQC, HMBC, and ROESY spectra were recorded. Light blue, orange, green, purple, dark yellow, and pink letters denote peaks derived from H_N, $H_{C\alpha}$, $H_{C\beta}$, $H_{C\delta}$, $H_{C\gamma}$, and H_{aryl}, respectively. The one-letter residue code and the number above a peak indicate the residue number to which the proton belongs.

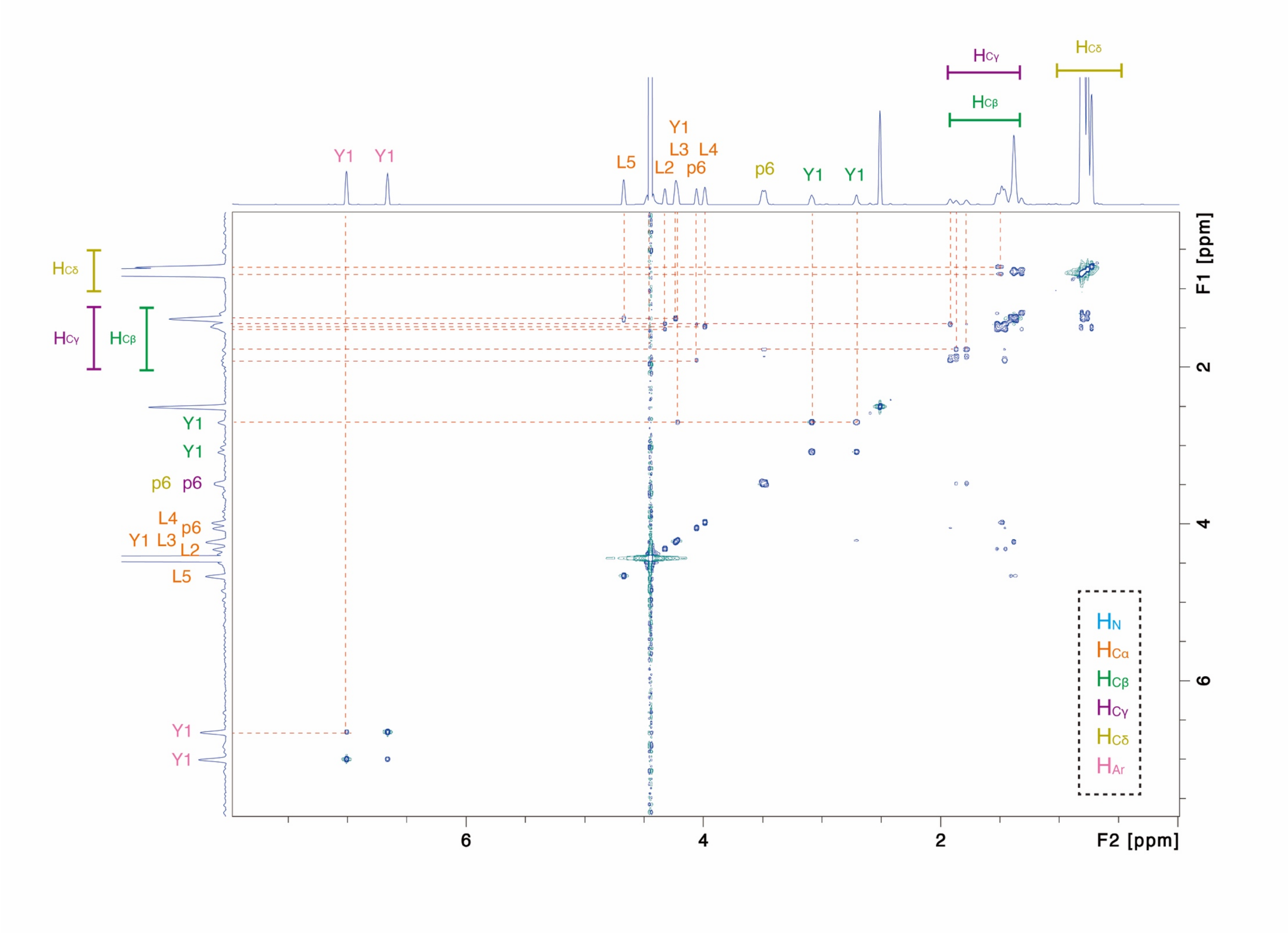

Supplementary Figure 31. COSY-NMR of CP1 in 50% DMSO/D_2O

The COSY-NMR spectrum of **CP1** was recorded in 50% DMSO/H_2O. Correlation peaks that support the assignment of ^{1}H-NMR are shown with pink dashed lines.

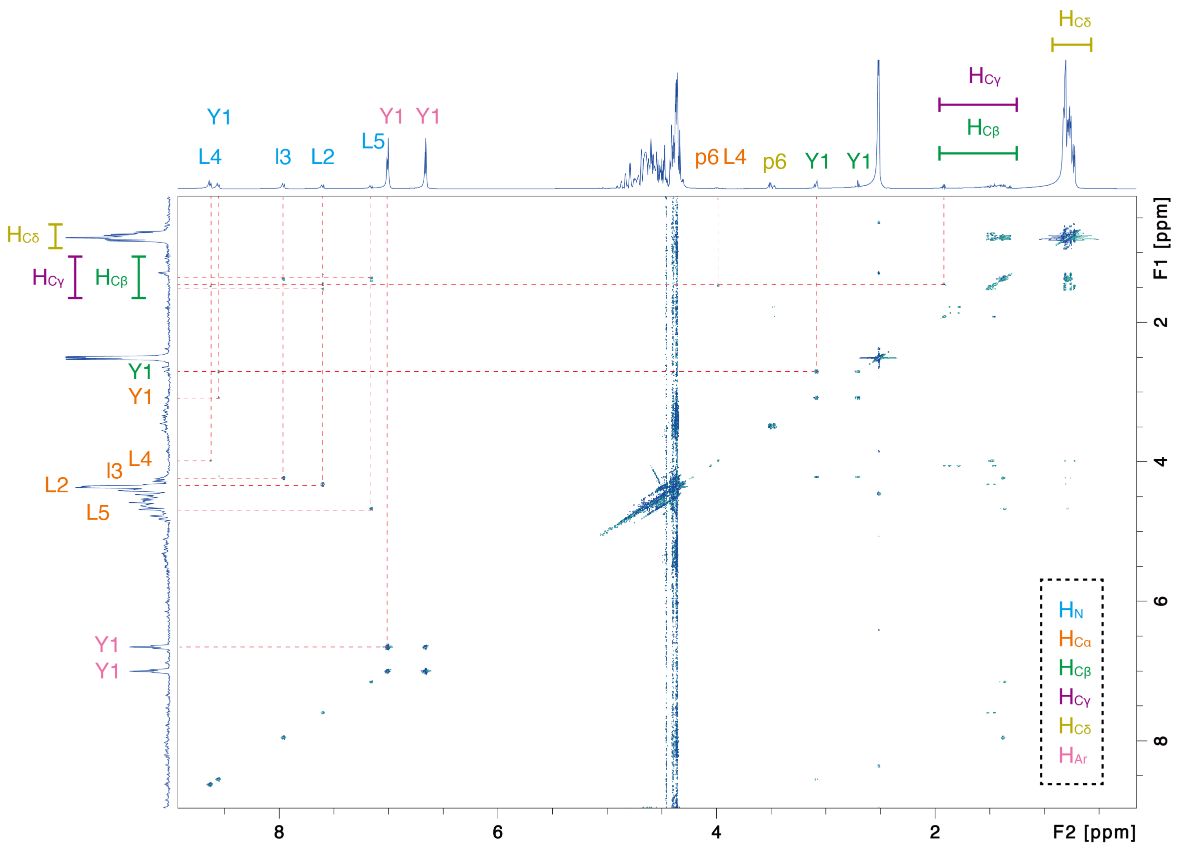

Supplementary Figure 32. TOCSY spectrum of CP1 in 50% DMSO/H_2O

The TOCSY-NMR spectrum of **CP1** was recorded in 50% DMSO/H_2O. Correlation peaks that support the assignment of ^{1}H-NMR are shown with pink dashed lines.

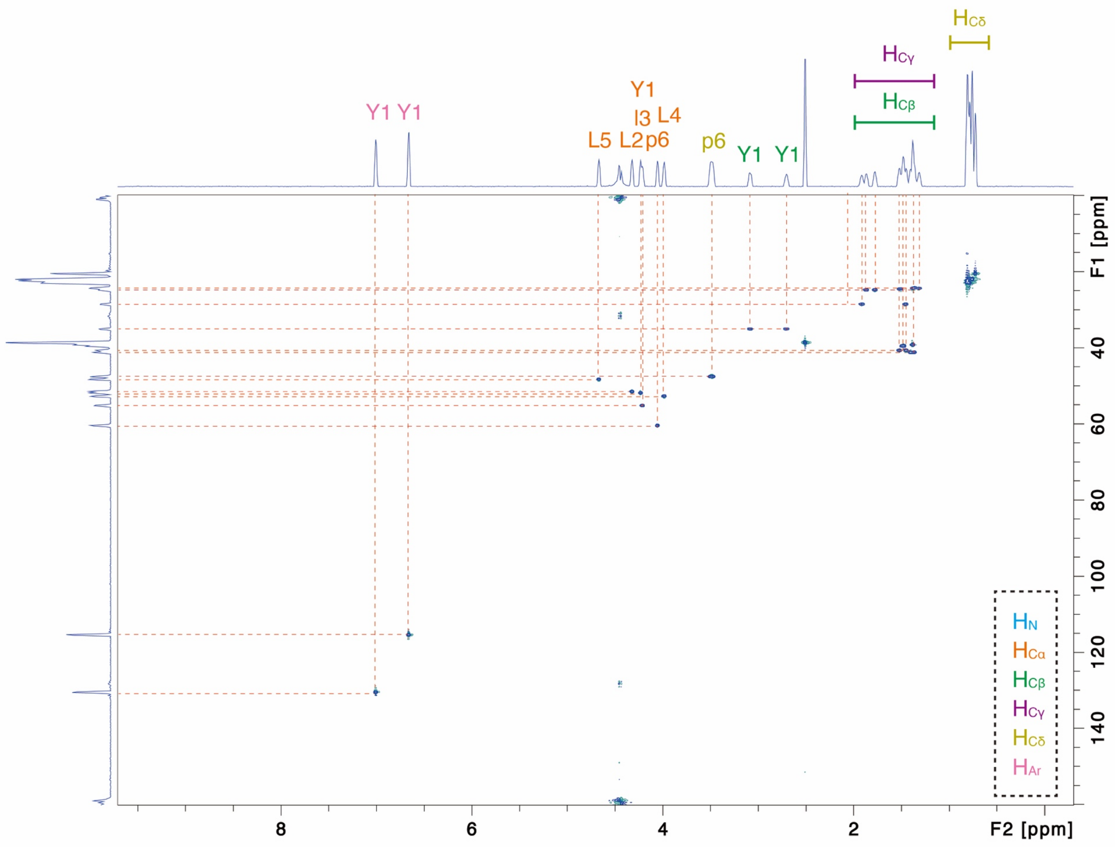

Supplementary Figure 33. HSQC spectrum of CP1 in 50% DMSO/D_2O

The HSQC-NMR spectrum of **CP1** was recorded in 50% DMSO/D_2O. Correlation peaks that support the assignment of ^{1}H-NMR are shown with pink dashed lines.

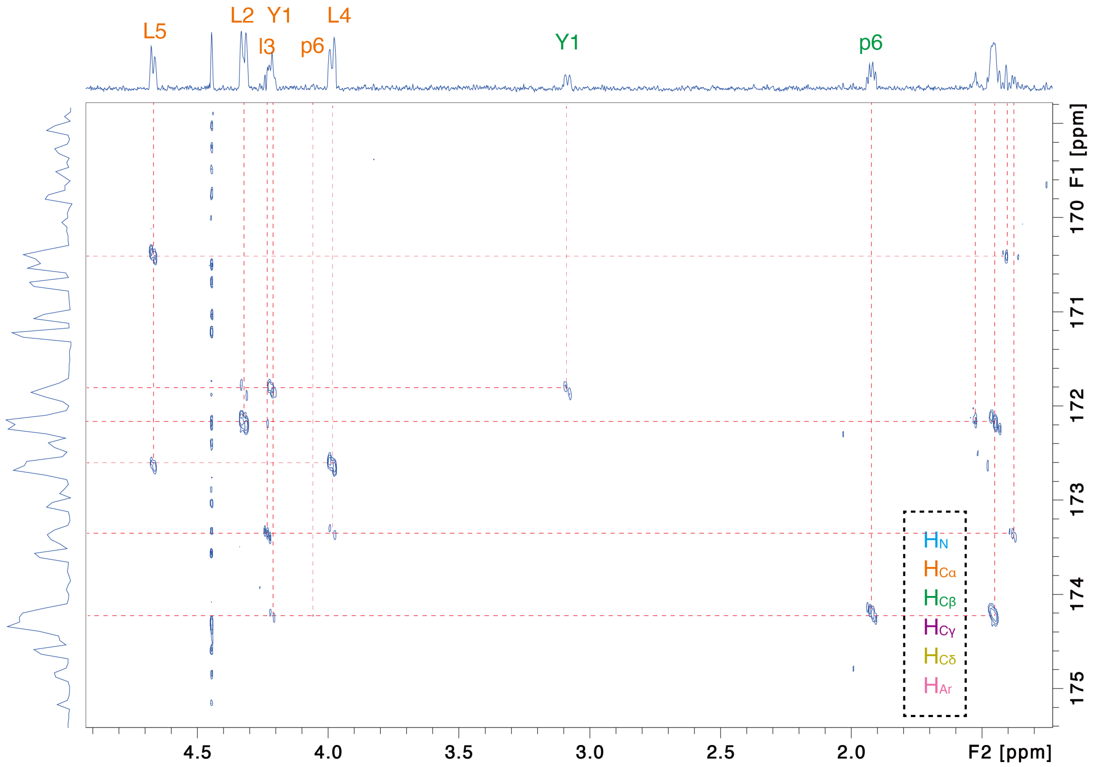

Supplementary Figure 34. HMBC spectrum of CP1 in 50% DMSO/D_2O

The HMBC-NMR spectrum of **CP1** was recorded in 50% DMSO/D_2O. The magnified spectrum with the correlation area of carbonyl carbon and $H_{C\alpha}$ was shown. Correlation peaks that support the assignment of ^{1}H-NMR are shown with pink dashed lines.

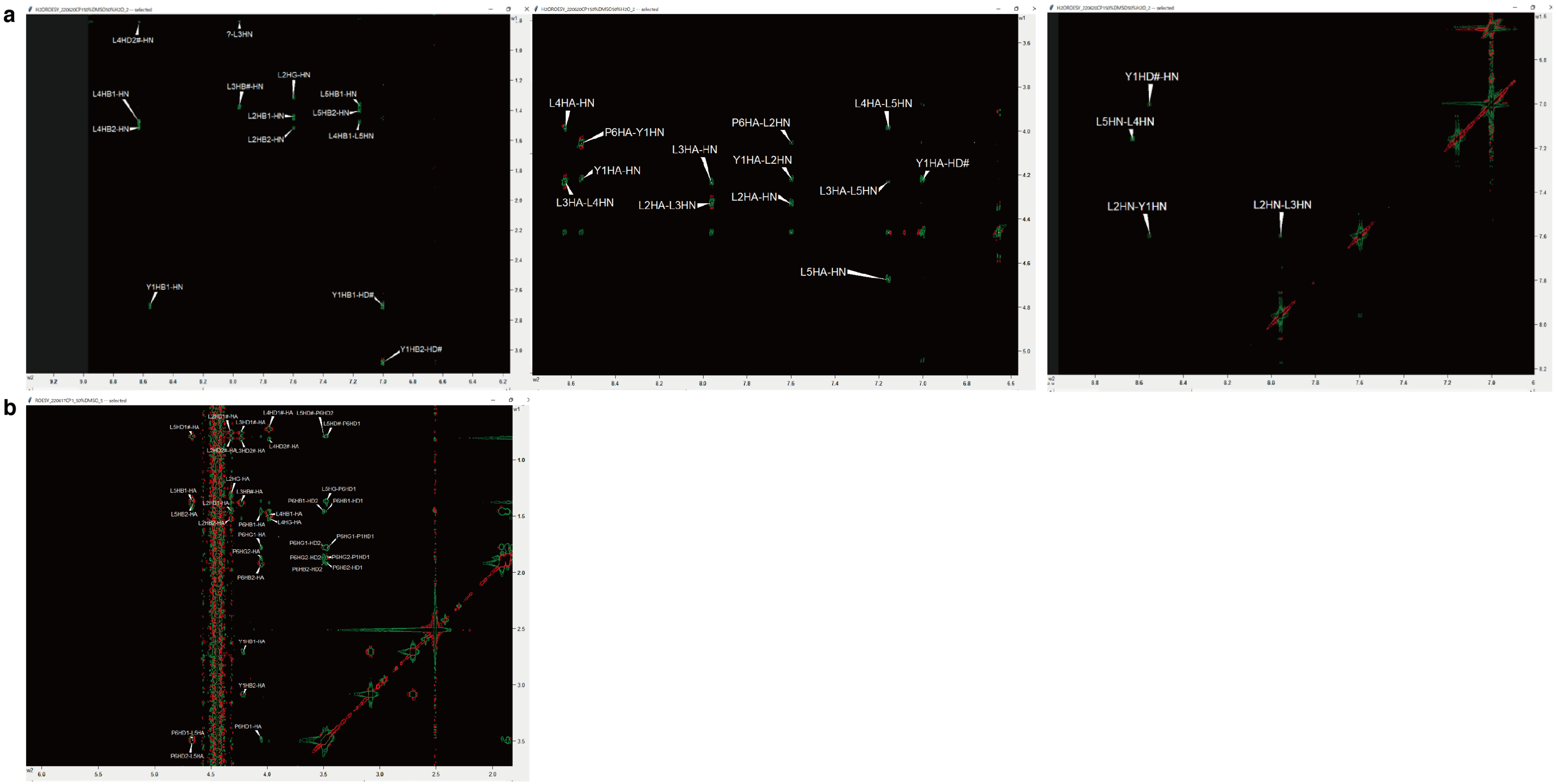

Supplementary Figure 35. ROESY spectrum of CP1 in 50% DMSO/H_2O and 50% DMSO/D_2O

The ROESY-NMR spectrum of **CP1** was recorded in (a) 50% DMSO/H_2O and (b) 50% DMSO/D_2O. Correlation peaks indicating the proximities of protons are indicated with the names of the proximal two protons. The correlation peaks are summarized in Supplementary Tables.

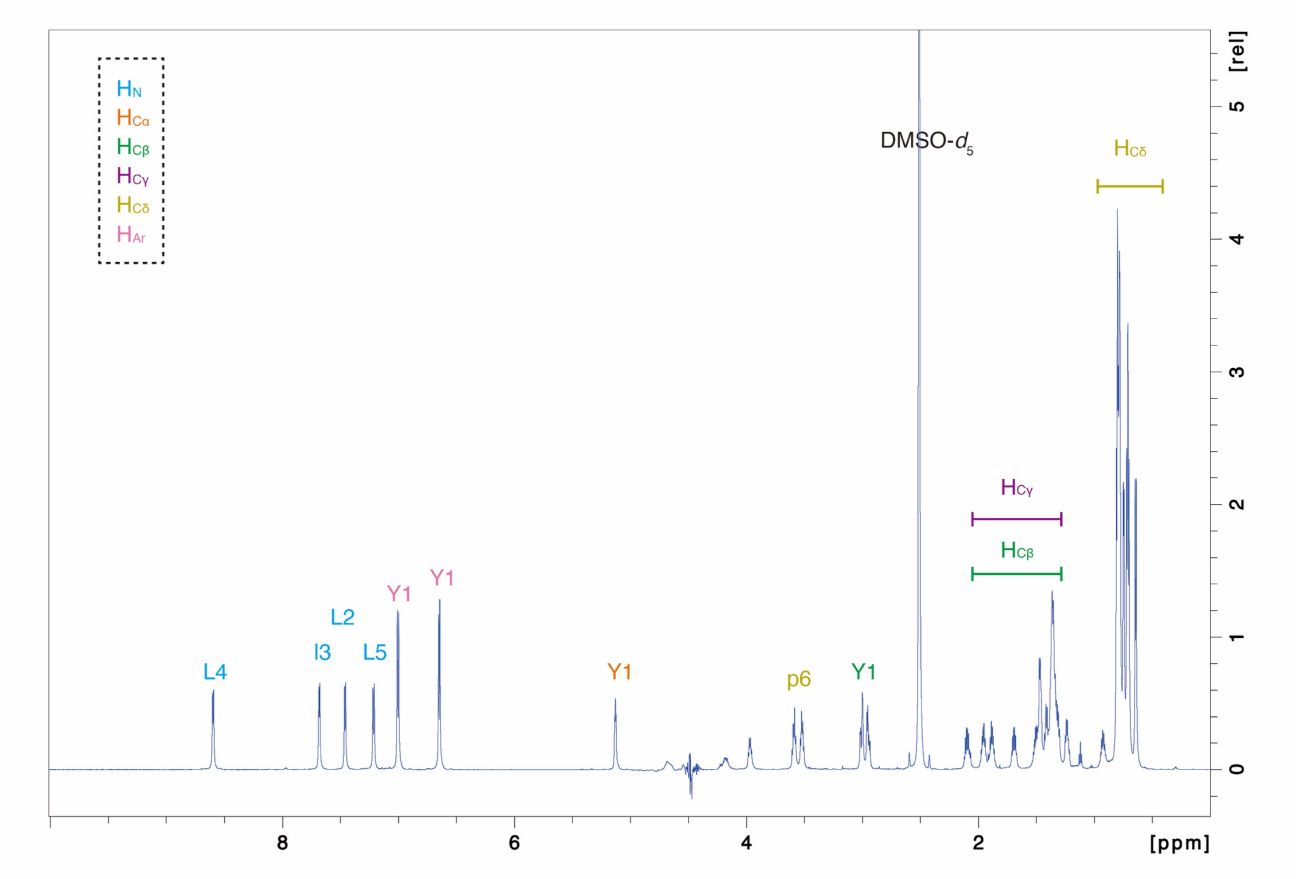

Supplementary Figure 36. ¹H-NMR of DP1 in 50% DMSO/H₂O

The ^{1}H-NMR spectrum of **DP1** was recorded in 50% DMSO/H_2O. For the assignment of each peak, COSY, TOCSY, HSQC, HMBC, and ROESY spectra were recorded. Light blue, orange, green, purple, dark yellow and pink letters denote peaks derived from H_N, $H_{C\alpha}$, $H_{C\beta}$, $H_{C\delta}$, $H_{C\gamma}$, and H_{aryl}, respectively. The one-letter residue code and the number above a peak indicate the residue number to which the proton belongs.

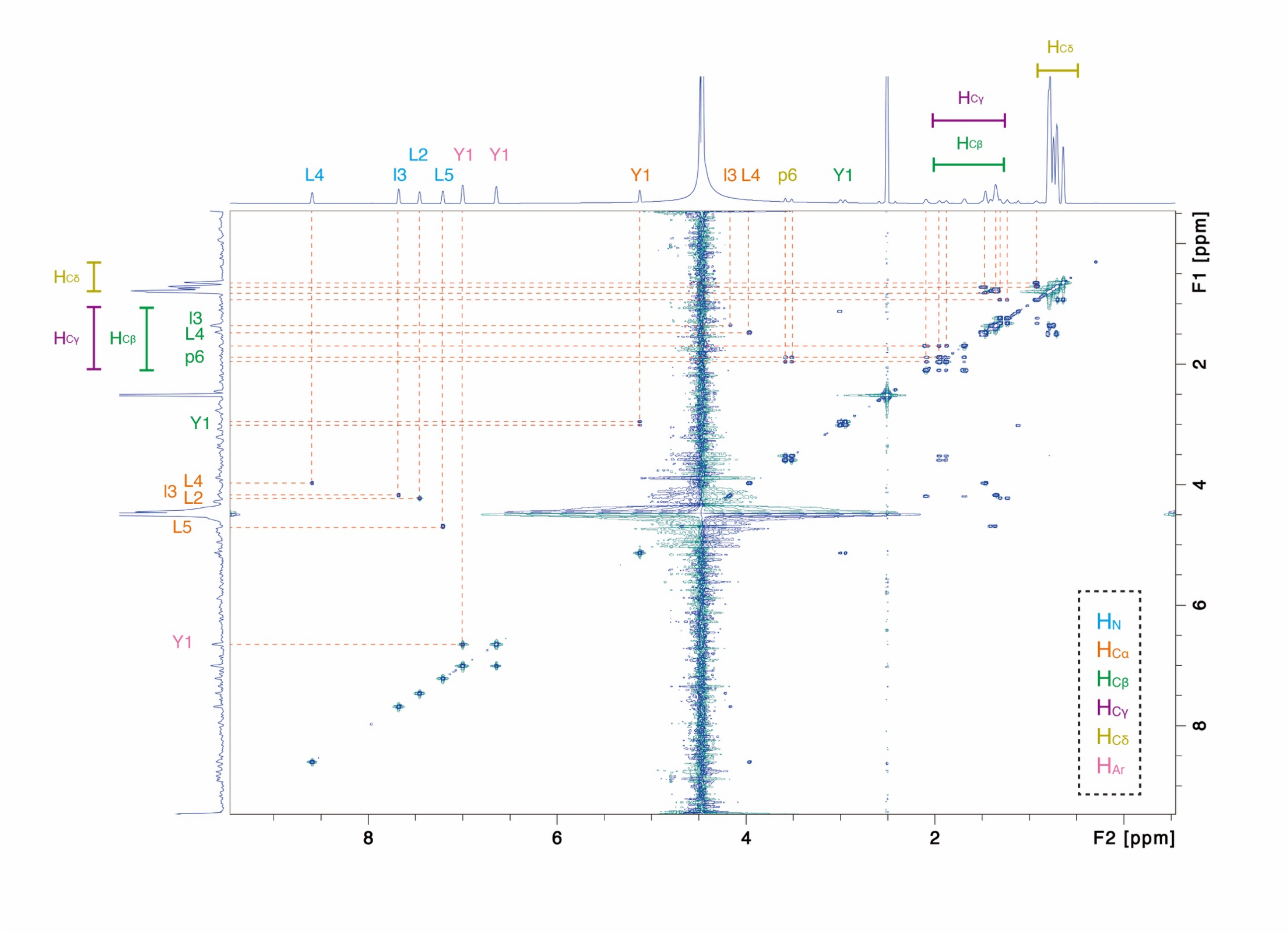

Supplementary Figure 37. COSY-NMR of DP1 in 50% DMSO/H_2O

The COSY-NMR spectrum of **DP1** was recorded in 50% DMSO/H_2O. Correlation peaks that support the assignment of ^{1}H-NMR are shown with pink dashed lines.

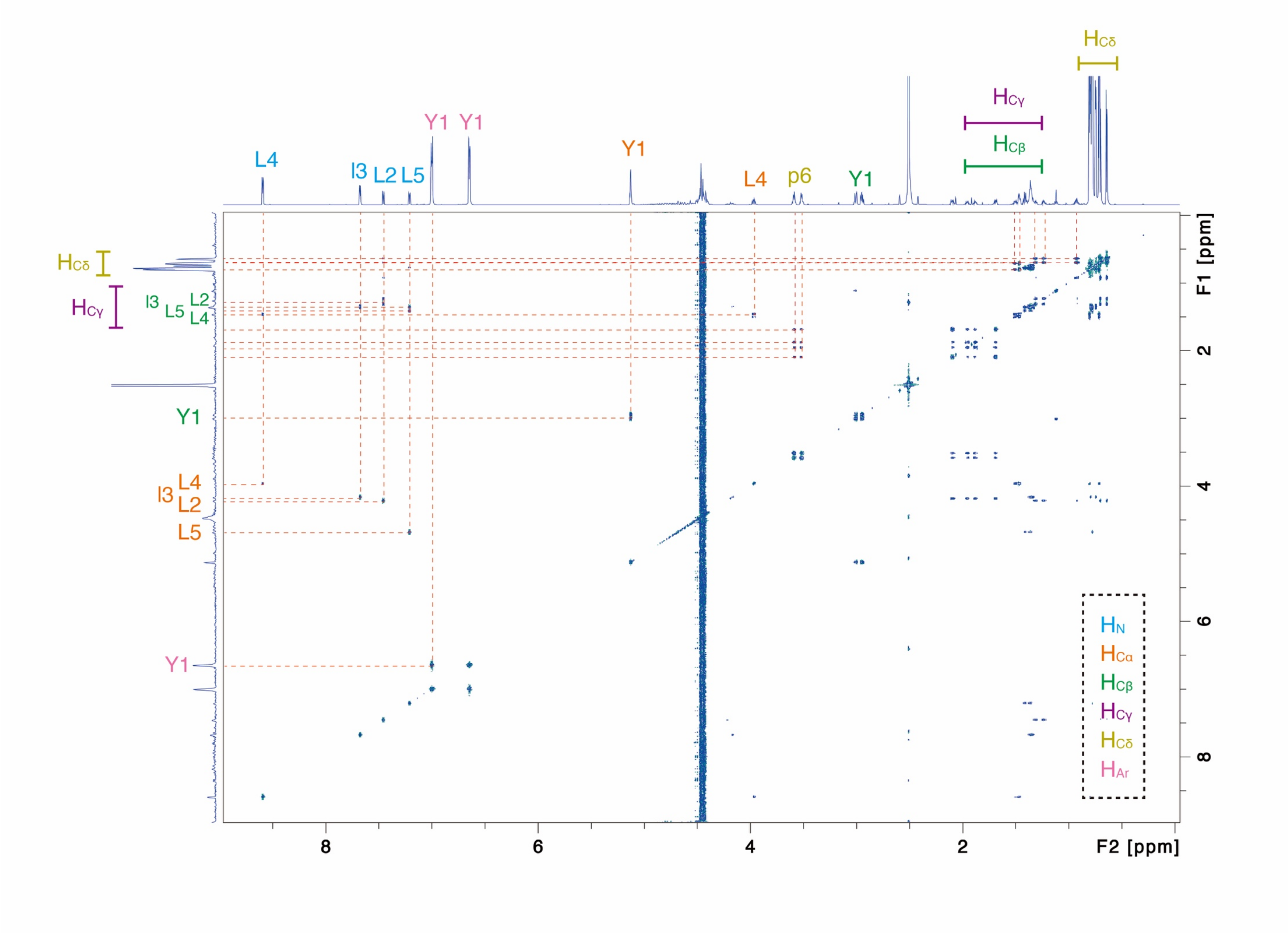

Supplementary Figure 38. TOCSY spectrum of DP1 in 50% DMSO/H2O

The TOCSY-NMR spectrum of **DP1** was recorded in 50% DMSO/H2O. Correlation peaks that support the assignment of ^{1}H-NMR are shown with pink dashed lines.

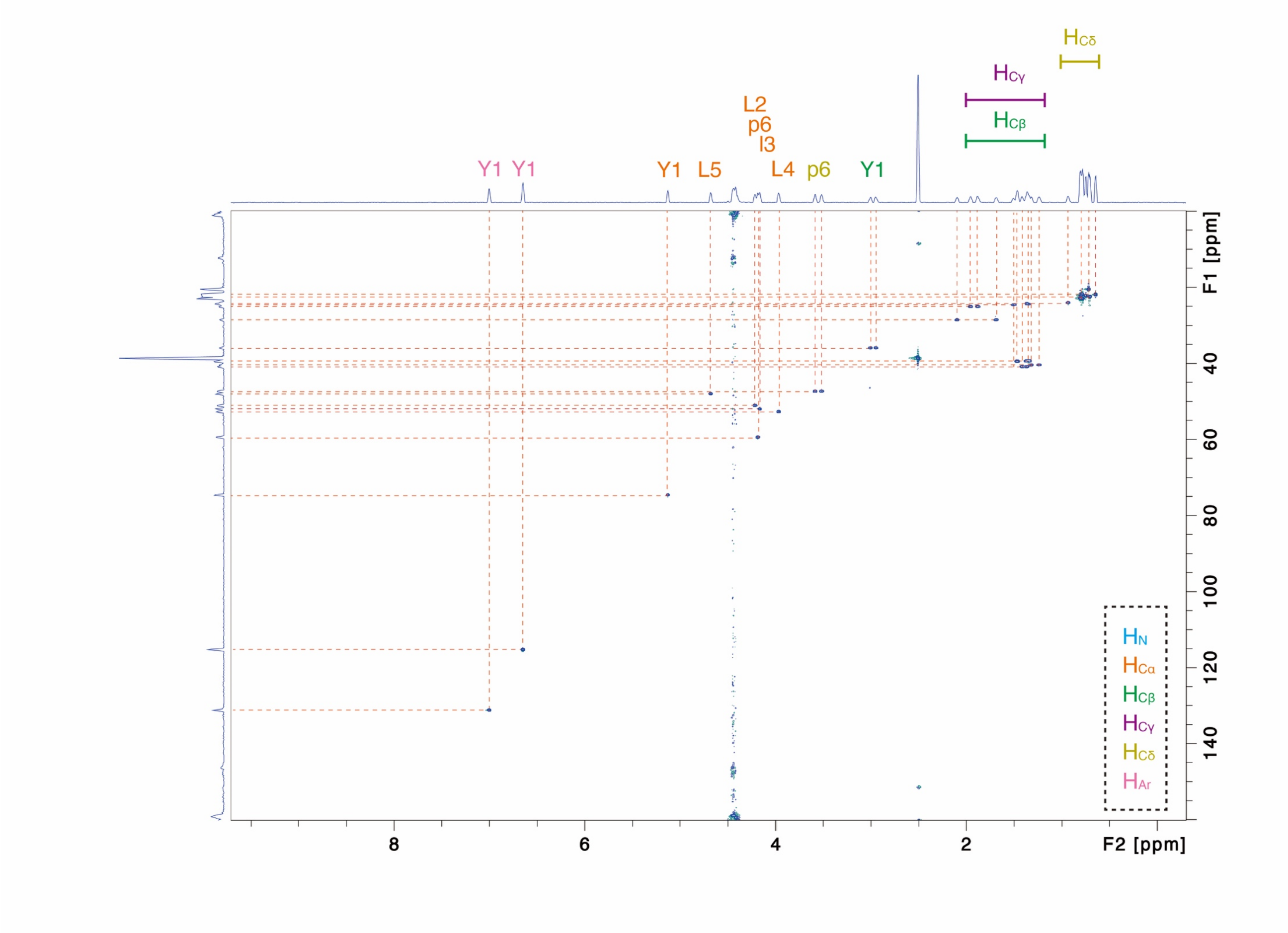

Supplementary Figure 39. HSQC spectrum of DP1 in 50% DMSO/D_2O

The HSQC-NMR spectrum of **DP1** was recorded in 50% DMSO/D_2O. Correlation peaks that support the assignment of ^{1}H-NMR are shown with pink dashed lines.

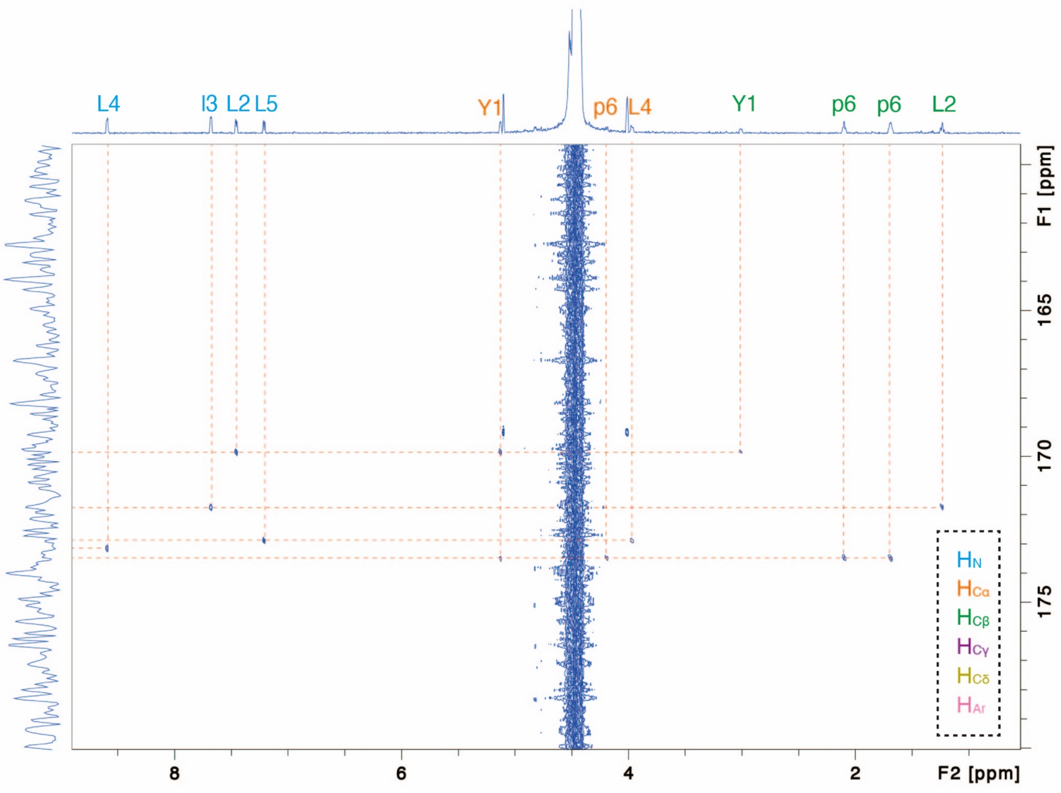

Supplementary Figure 40. HMBC spectrum of DP1 in 50% DMSO/H_2O

The HMBC-NMR spectrum of **DP1** was recorded in 50% DMSO/H_2O. Correlation peaks that support the assignment of ^{1}H-NMR are shown with pink dashed lines.

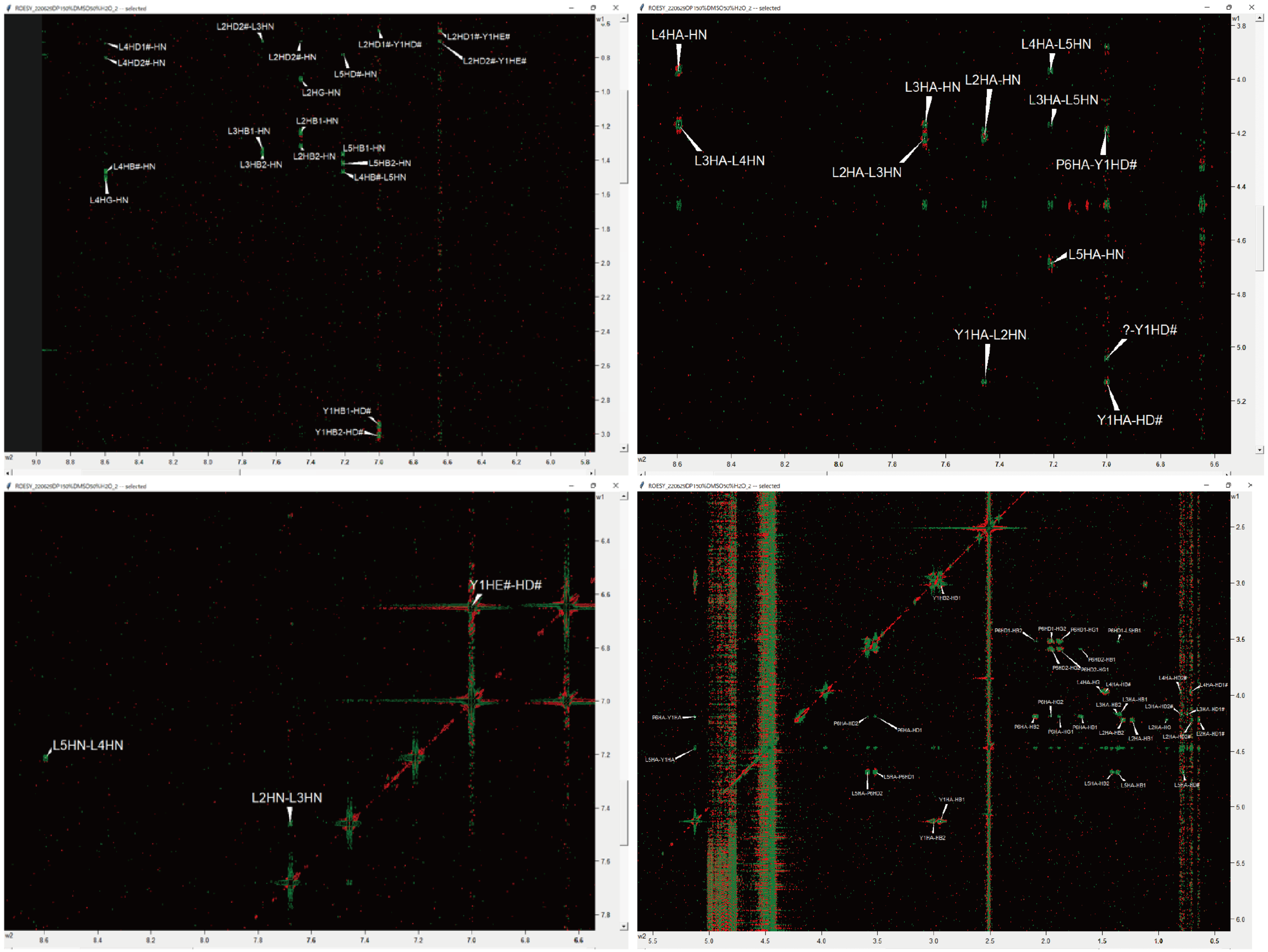

Supplementary Figure 41. ROESY spectrum of DP1 in 50% DMSO/H_2O

The ROESY-NMR spectrum of **DP1** was recorded in 50% DMSO/H_2O. The correlation peaks are summarized in Supplementary Tables.

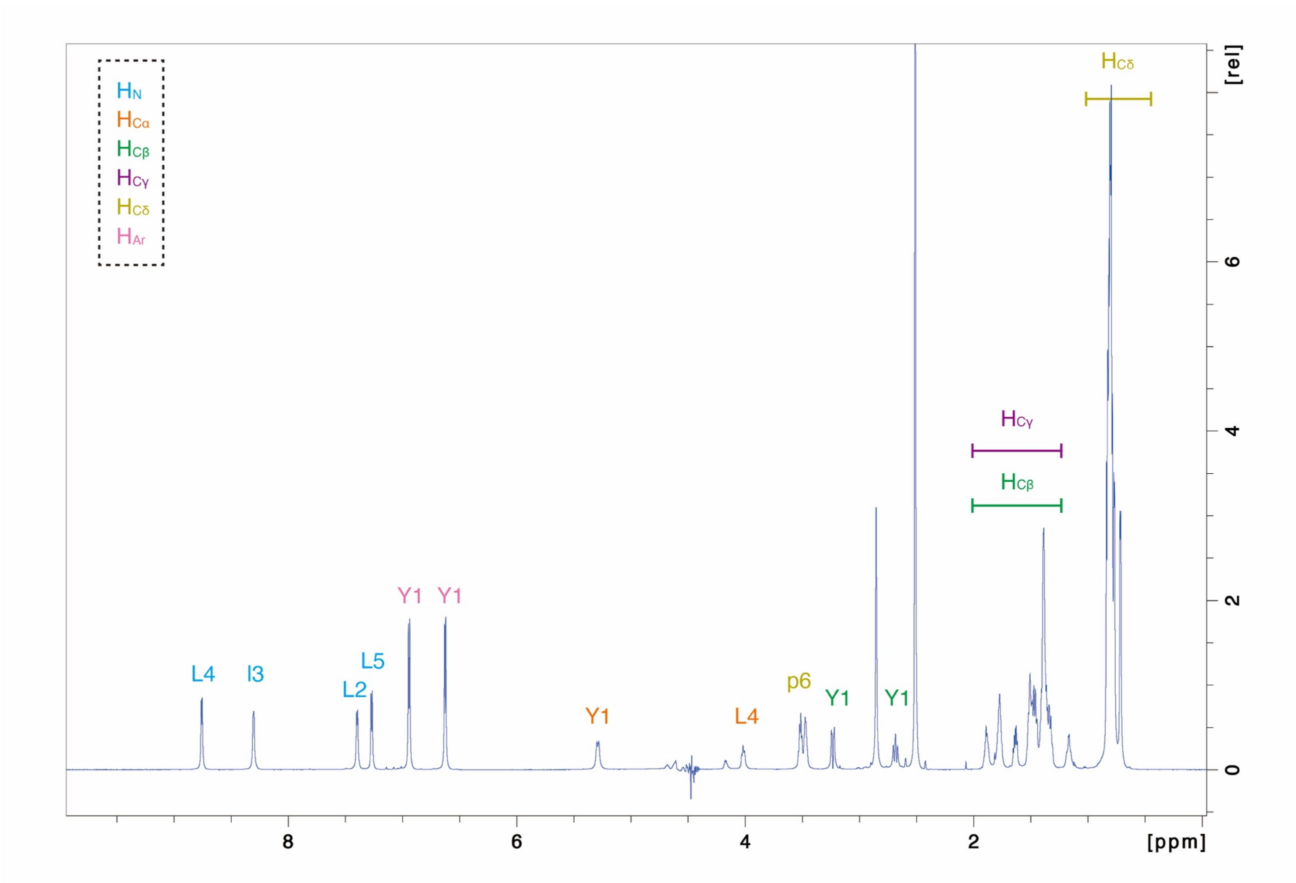

Supplementary Figure 42. ^{1}H-NMR of MP1 in 50% DMSO/H$_2$O

The ^{1}H-NMR spectrum of **MP1** was recorded in 50% DMSO/H_2O. For the assignment of each peak, COSY, TOCSY, HSQC, HMBC, and ROESY spectra were recorded. Light blue, orange, green, purple, dark yellow, and pink letters denote peaks derived from H_N, $H_{C\alpha}$, $H_{C\beta}$, $H_{C\delta}$, $H_{C\gamma}$, and H_{aryl}, respectively. The one-letter residue code and the number above a peak indicate the residue number to which the proton belongs.

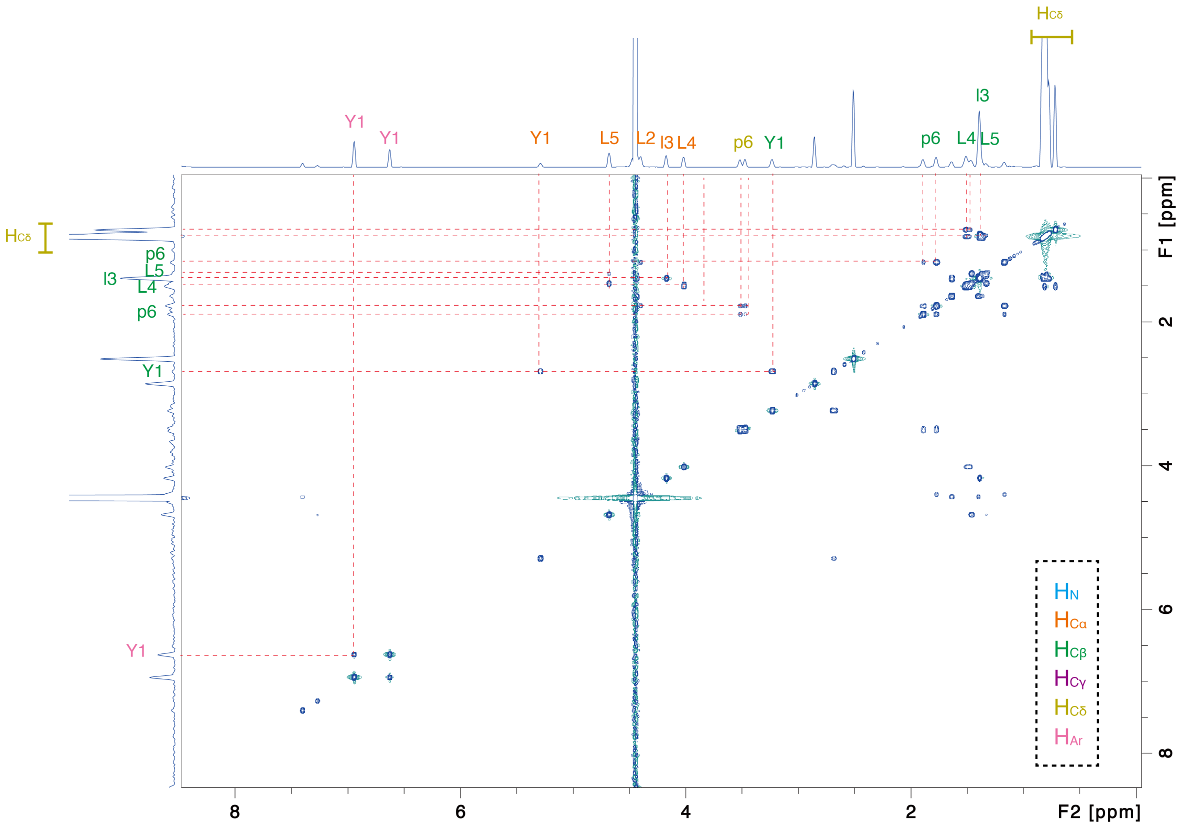

Supplementary Figure 43. COSY-NMR of MP1 in 50% DMSO/D$_2$O

The COSY-NMR spectrum of **MP1** was recorded in 50% DMSO/D$_2$O. Correlation peaks that support the assignment of ^{1}H-NMR are shown with pink dashed lines.

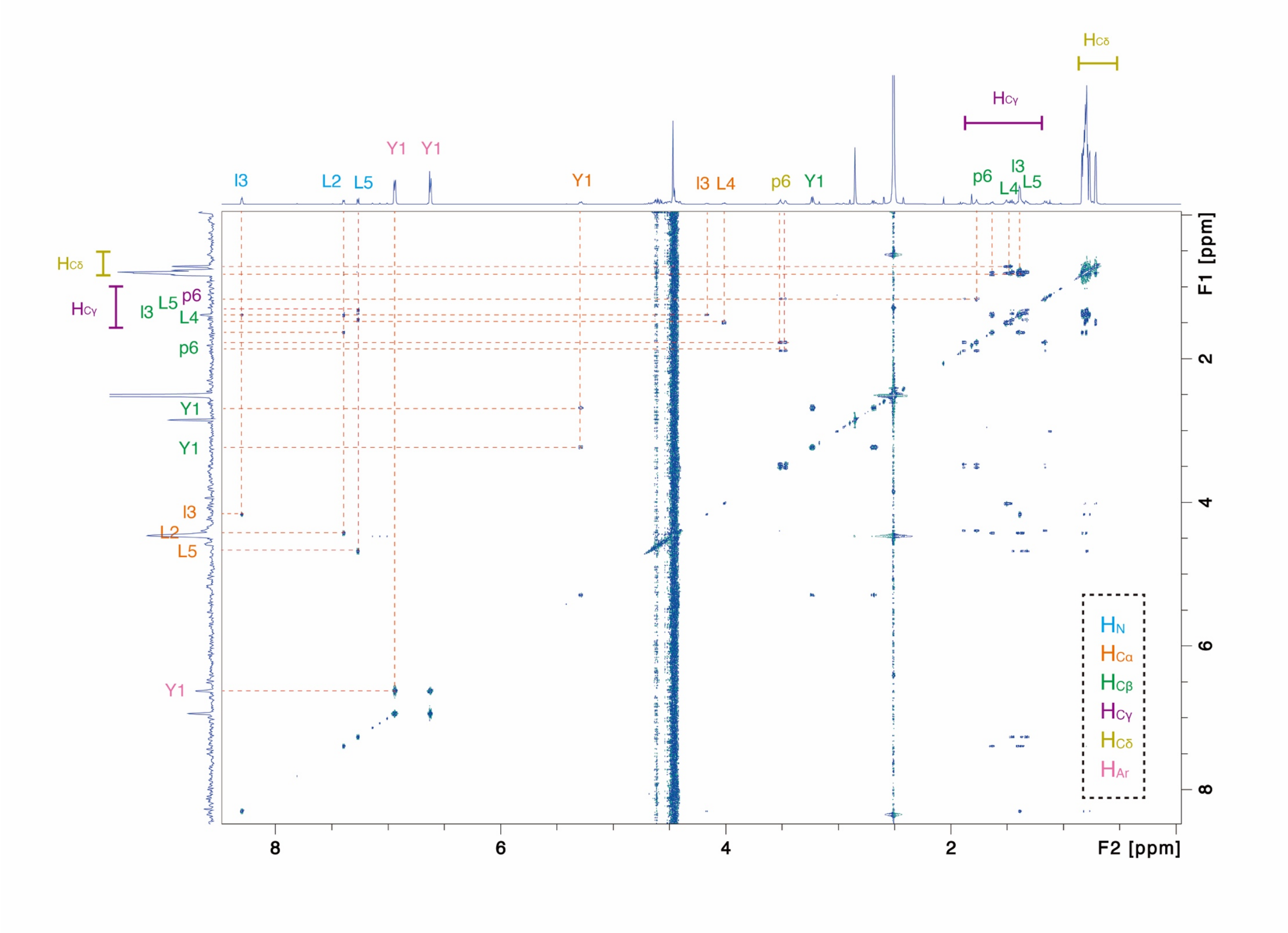

Supplementary Figure 44. TOCSY spectrum of MP1 in 50% DMSO/H2O

The TOCSY-NMR spectrum of **MP1** was recorded in 50% DMSO/H_2O. Correlation peaks that support the assignment of ^{1}H-NMR are shown with pink dashed lines.

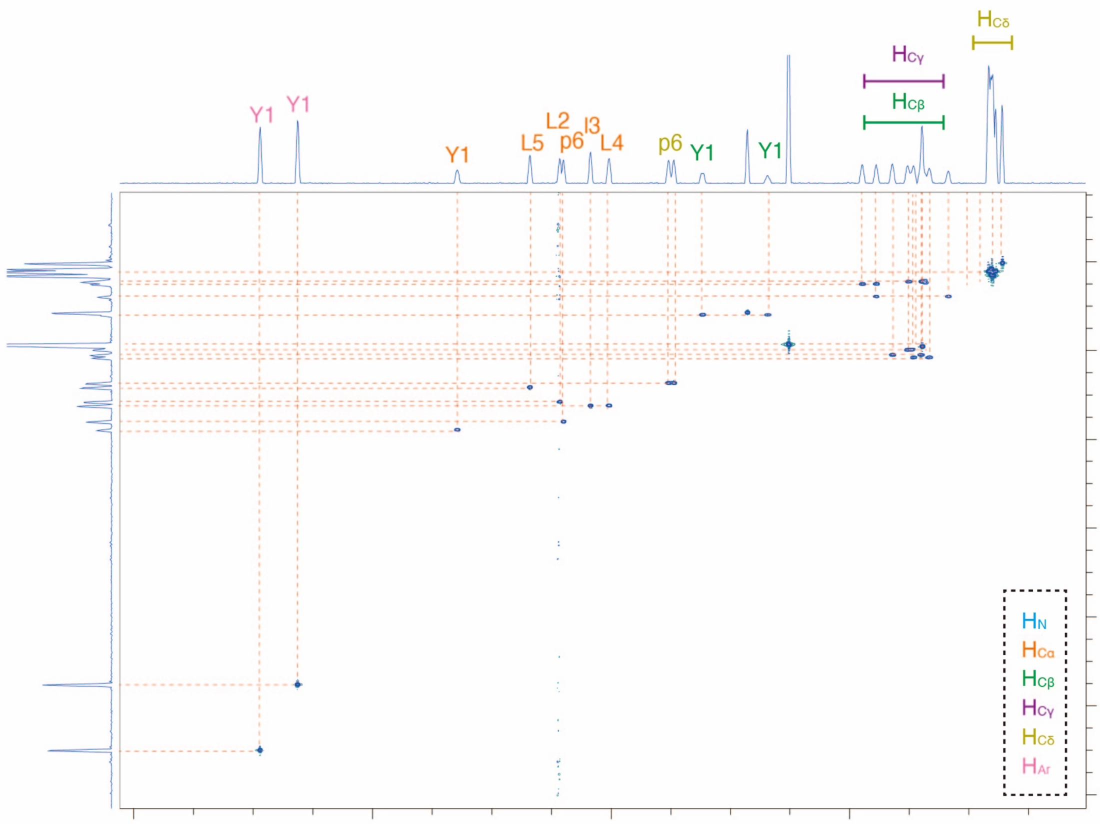

Supplementary Figure 45. HSQC spectrum of MP1 in 50% DMSO/D_2O

The HSQC-NMR spectrum of **MP1** was recorded in 50% DMSO/D_2O. Correlation peaks that support the assignment of ^{1}H-NMR are shown with pink dashed lines.

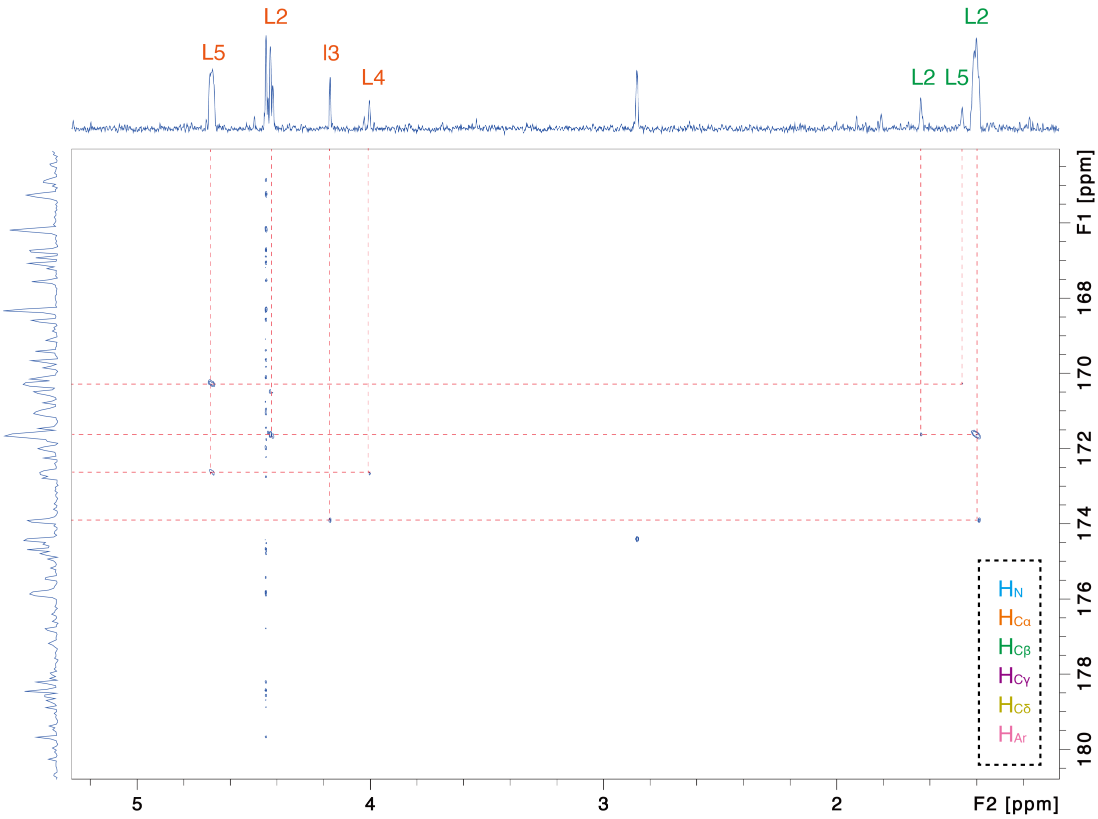

Supplementary Figure 46. HMBC spectrum of MP1 in 50% DMSO/D₂O

The HMBC-NMR spectrum of **MP1** was recorded in 50% DMSO/D_2O. Correlation peaks that support the assignment of ^{1}H-NMR are shown with pink dashed lines.

a

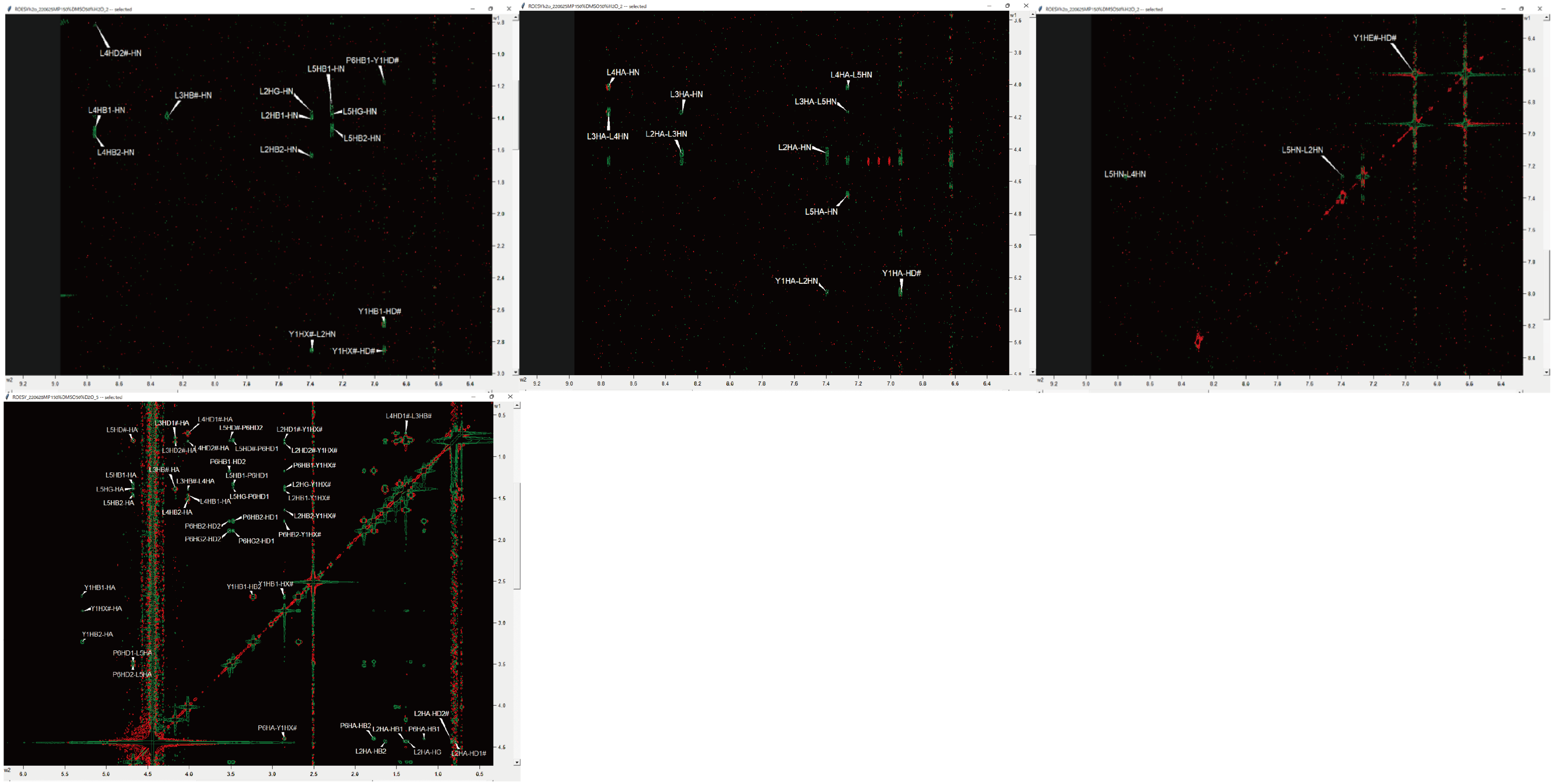

b

Supplementary Figure 47. ROESY spectrum of MP1 in 50% DMSO/H_2O and 50% DMSO/D_2O

The ROESY-NMR spectrum of **MP1** was recorded in (a) 50% DMSO/H_2O and (b) 50% DMSO/D_2O. The correlation peaks are summarized in Supplementary Tables.

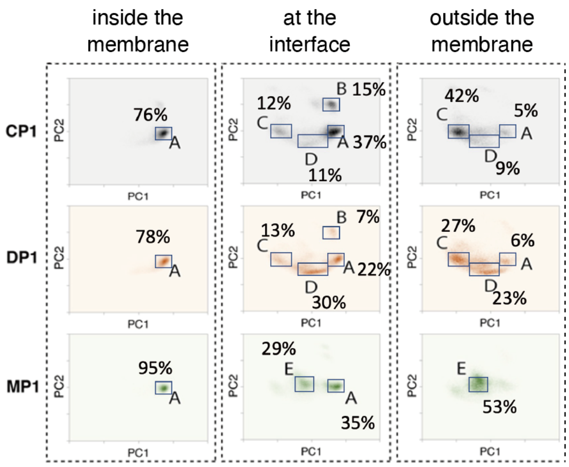

Supplementary Figure 48. The percentage of all conformations in each region in the two-dimensional PCA space from the MD simulations

Conformational ensembles of **CP1**, **DP1**, and **MP1** inside, at the interface, and outside the lipid membrane projected onto the first and second principal axes. The percentages of the major conformations are described.

a

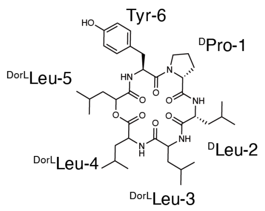

b

	backbone chirality					
Name	Pro-1	Leu-2	Leu-3	Leu-4	Leu-5	Tyr-6
CP2	D	D	D	D	L	L
CP3	D	D	L	D	L	L
CP4	D	D	L	L	L	L
CP5	D	D	D	L	D	L
CP6	D	D	L	L	D	L

c

	substitution and position					
Name	Pro-1	Leu-2	Leu-3	Leu-4	Leu-5	Tyr-6
**CP2**	NH	NH	NH	NH	NH	NH
**CP2-6YE**	NH	NH	NH	NH	NH	O
**CP2-6YM**	NH	NH	NH	NH	NH	NMe
**CP3**	NH	NH	NH	NH	NH	NH
**CP3-6YE**	NH	NH	NH	NH	NH	O
**CP3-6YM**	NH	NH	NH	NH	NH	NMe
**CP4**	NH	NH	NH	NH	NH	NH
**CP4-6YE**	NH	NH	NH	NH	NH	O
**CP4-6YM**	NH	NH	NH	NH	NH	NMe
**CP5**	NH	NH	NH	NH	NH	NH
**CP5-6YE**	NH	NH	NH	NH	NH	O
**CP5-6YM**	NH	NH	NH	NH	NH	NMe
**CP6**	NH	NH	NH	NH	NH	NH
**CP6-6YE**	NH	NH	NH	NH	NH	O
**CP6-6YM**	NH	NH	NH	NH	NH	NMe

d

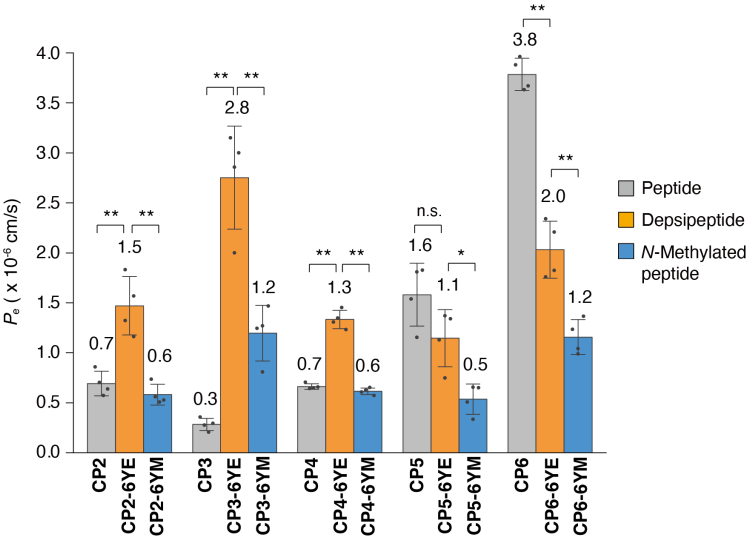

Supplementary Figure 49. PAMPA of cyclic hexapeptides CP2–CP6 and their derivatives

(a) General chemical structure of **CP2–6** and (b) backbone stereochemistries of **CP2–6**.[7] The backbone stereochemistries on Leu-3, Leu-4, and Leu-5 are different among the compounds. L- and D-amino acid residue is highlighted in blue and red, respectively. (c) The position of the introduction of an amide-to-ester substitution or an amide *N*-methylation. The position of an amide-to-ester substitution and amide *N*-methylation is shown by O highlighted in orange and NMe highlighted in blue, respectively. (d) The permeability of compounds measured by PAMPA. 3 μM compounds dissolved in 5% DMSO/PBS (pH 7.4) were incubated in donor wells docked with acceptor wells containing 5% DMSO/PBS (pH 7.4) for 16 h at 25 °C. After the incubation, the concentration of compounds in each well was measured by LC-MS to calculate their permeability. Each bar represents the mean value, and the error bars the standard deviation from experiments carried out in triplicate. P values were determined by a two-sided Welch's *t*-test. ** $p < 0.01$, * $p < 0.05$, n.s. denotes not significant. p (**CP2** vs. **CP2-6YE**) = 0.0078, p (**CP2-6YE** vs. **CP2-6YM**) = 0.0056, p (**CP3** vs. **CP3-6YE**) = 0.0022, p (**CP3-6YE** vs. **CP3-6YM**) = 0.0040, p (**CP4** vs. **CP4-6YE**) = 0.0003, p (**CP4-6YE** vs. **CP4-6YM**) = 0.0002, p (**CP5** vs. **CP5-6YE**) = 0.0873, p (**CP5-6YE** vs. **CP5-6YM**) = 0.0155, p (**CP6** vs. **CP6-6YE**) = 0.0002 and p (**CP6-6YE** vs. **CP6-6YM**) = 0.0035.

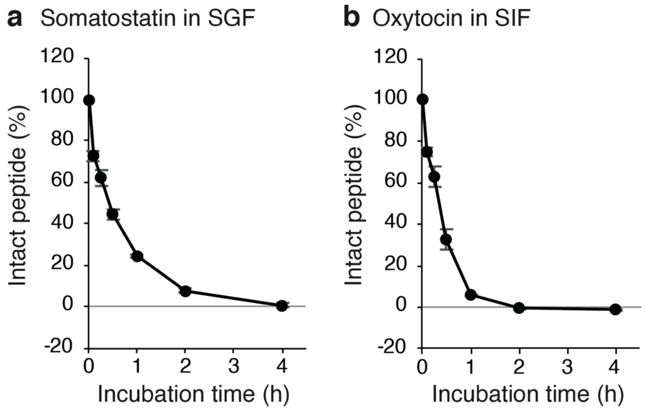

Supplementary Figure 50. Degradation profiles of control peptides in SGF and SIF

(a) Stability of somatostatin in simulated gut fluid (SGF). (b) Stability of oxytocin in simulated intestinal fluid (SIF).

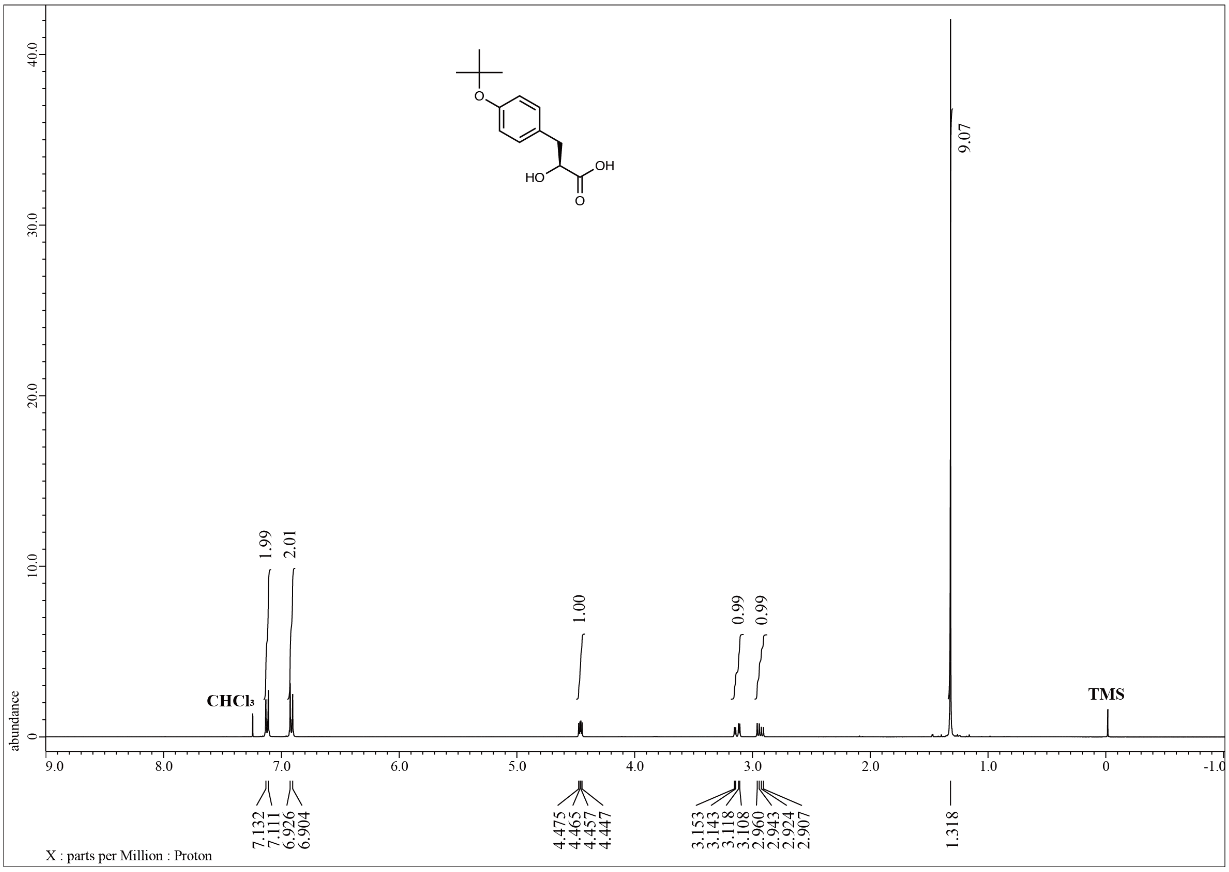

Supplementary Figure 51. ^{1}H-NMR spectrum of HO-Tyr(tBu)-OH

The ^{1}H-NMR spectrum of HO-Tyr(tBu)-OH was recorded in $CDCl_3$ at 298 K using an ECS-400 (JEOL) with a magnetic field of 400 MHz. Residual internal $CHCl_3$ (δ 7.26) was used as the standard.

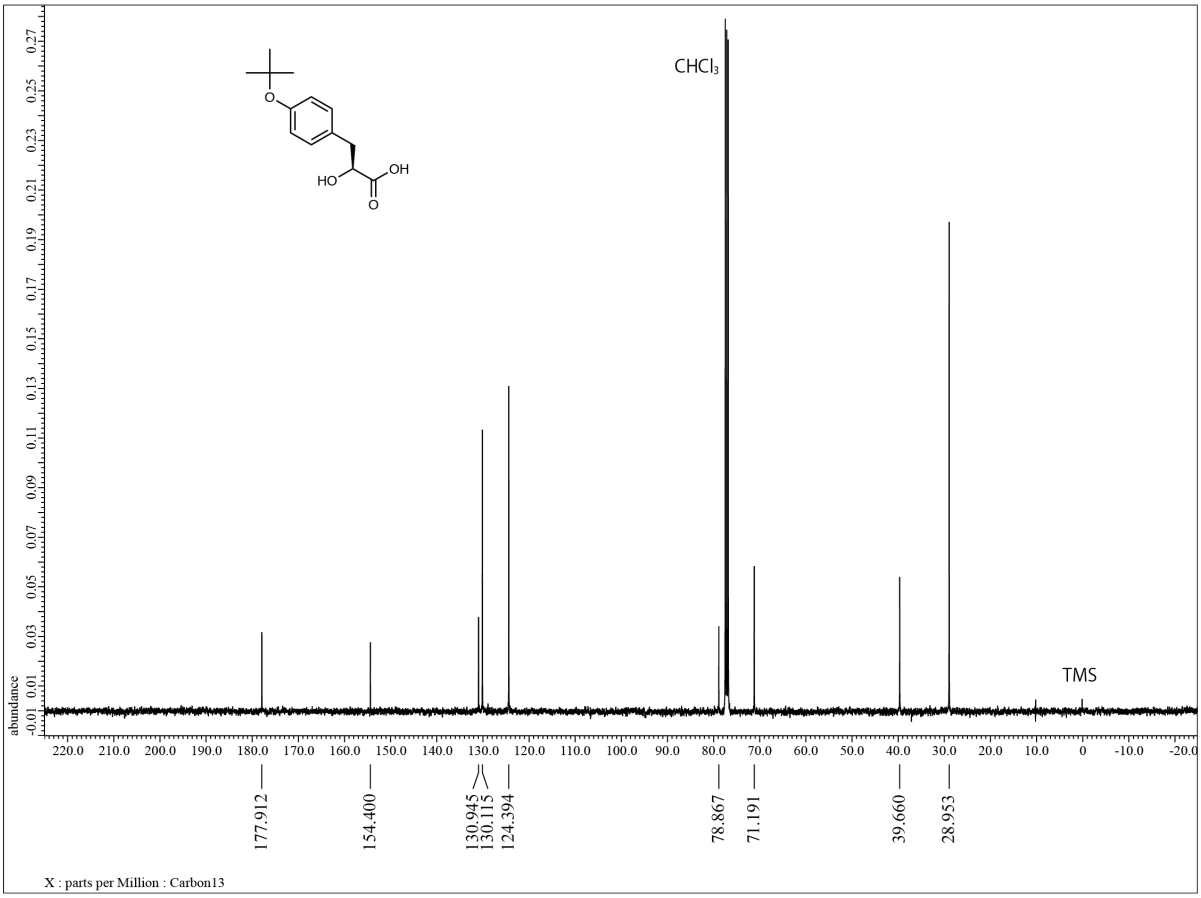

Supplementary Figure 52. ^{13}C-NMR spectrum of HO-Tyr(tBu)-OH

The ^{13}C-NMR spectrum of HO-Tyr(tBu)-OH was recorded in $CDCl_3$ at 298 K using an ECS-400 (JEOL) with a magnetic field of 400 MHz. Solvent $CDCl_3$ peak (δ 77.16) was used as the standard.

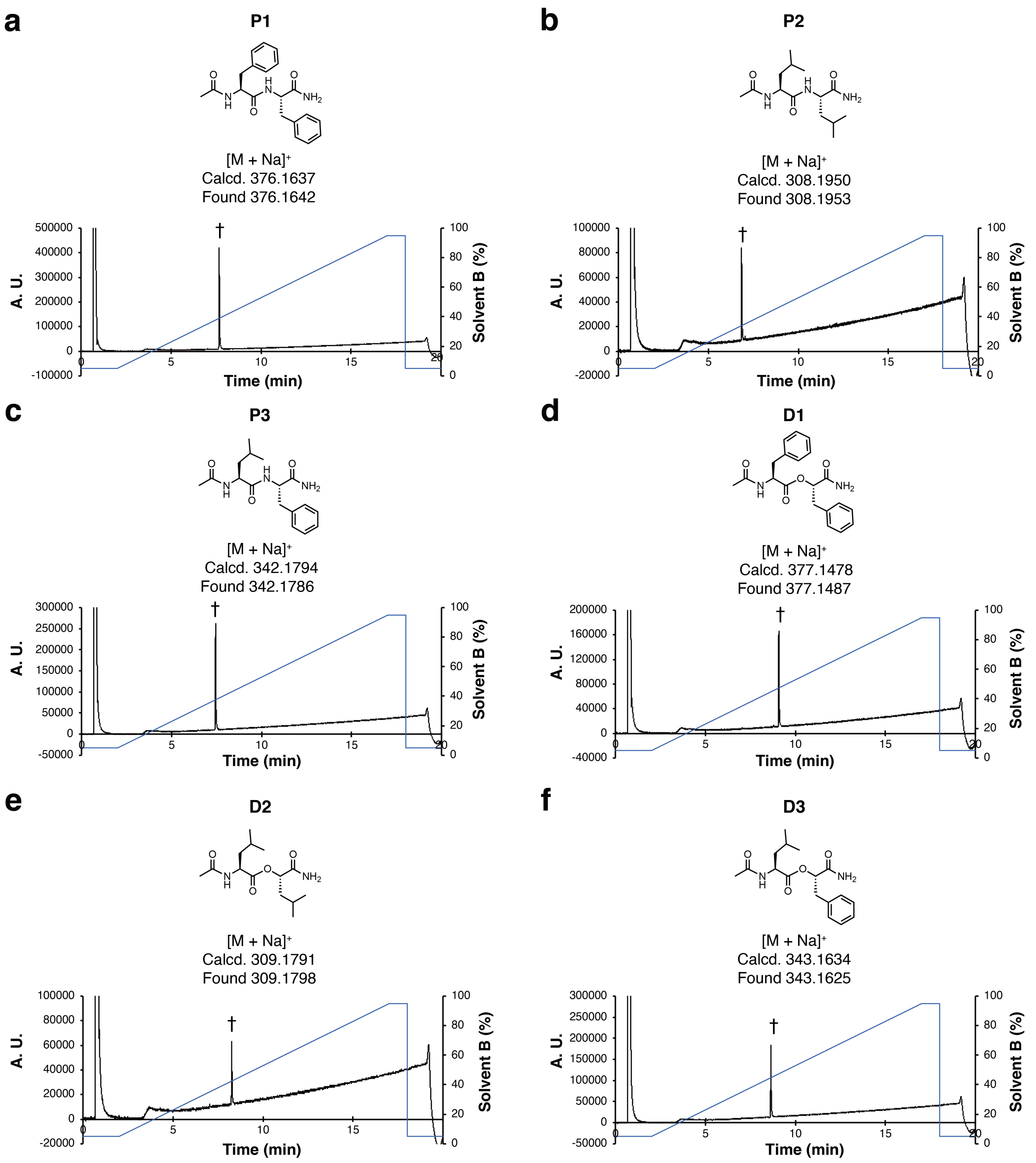

Supplementary Figure 53. UPLC chromatograms of P1–3, D1–3, and M1–3

† denotes the fraction containing desired product. The calculated and found mass values of the desired compound are labeled under the chemical structure of each compound. (a) **P1**, (b) **P2**, (c) **P3**, (d) **D1**, (e) **D2**, (f) **D3**.

[cont.]

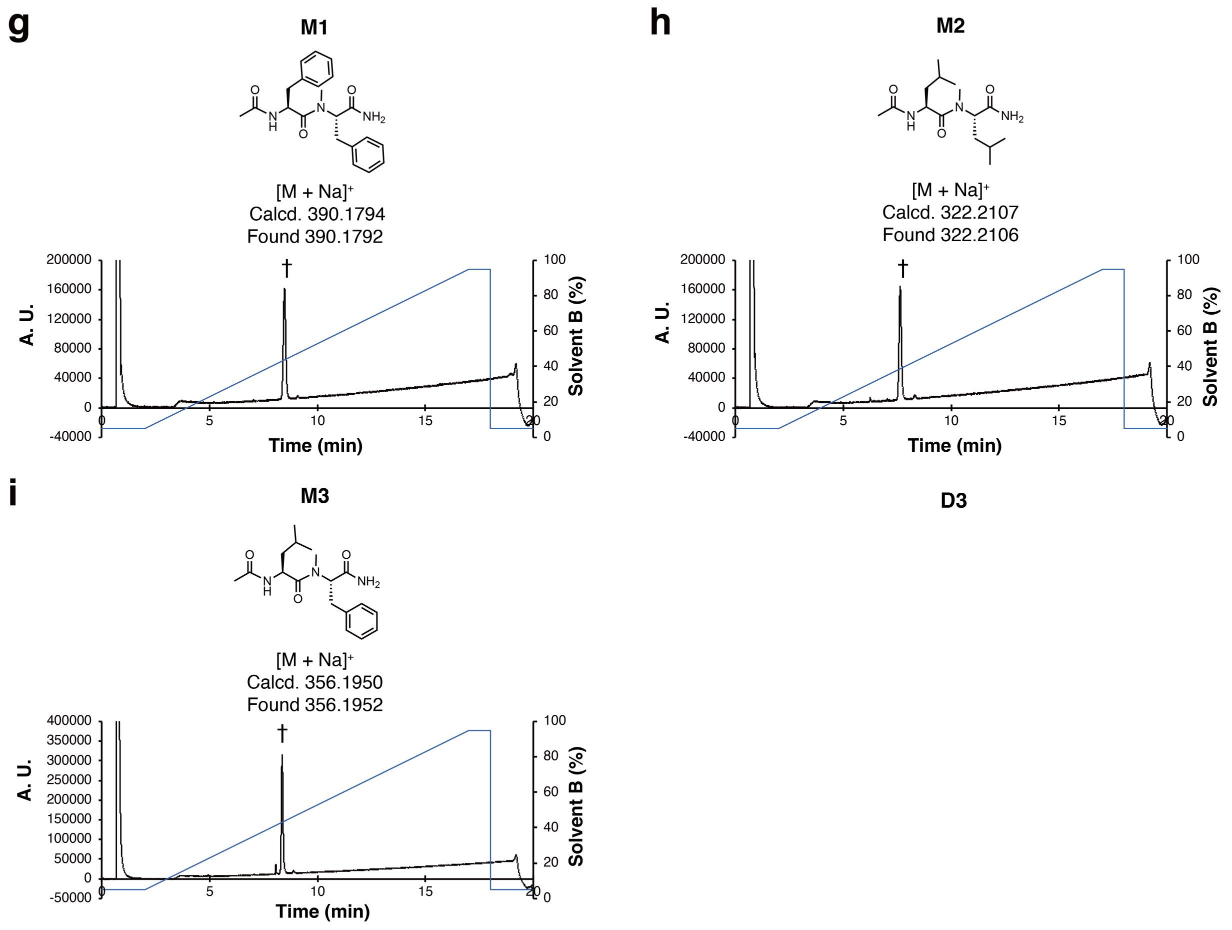

UPLC chromatograms of (g) **M1**, (h) **M2**, and (i) **M3**.

[cont. from p65]

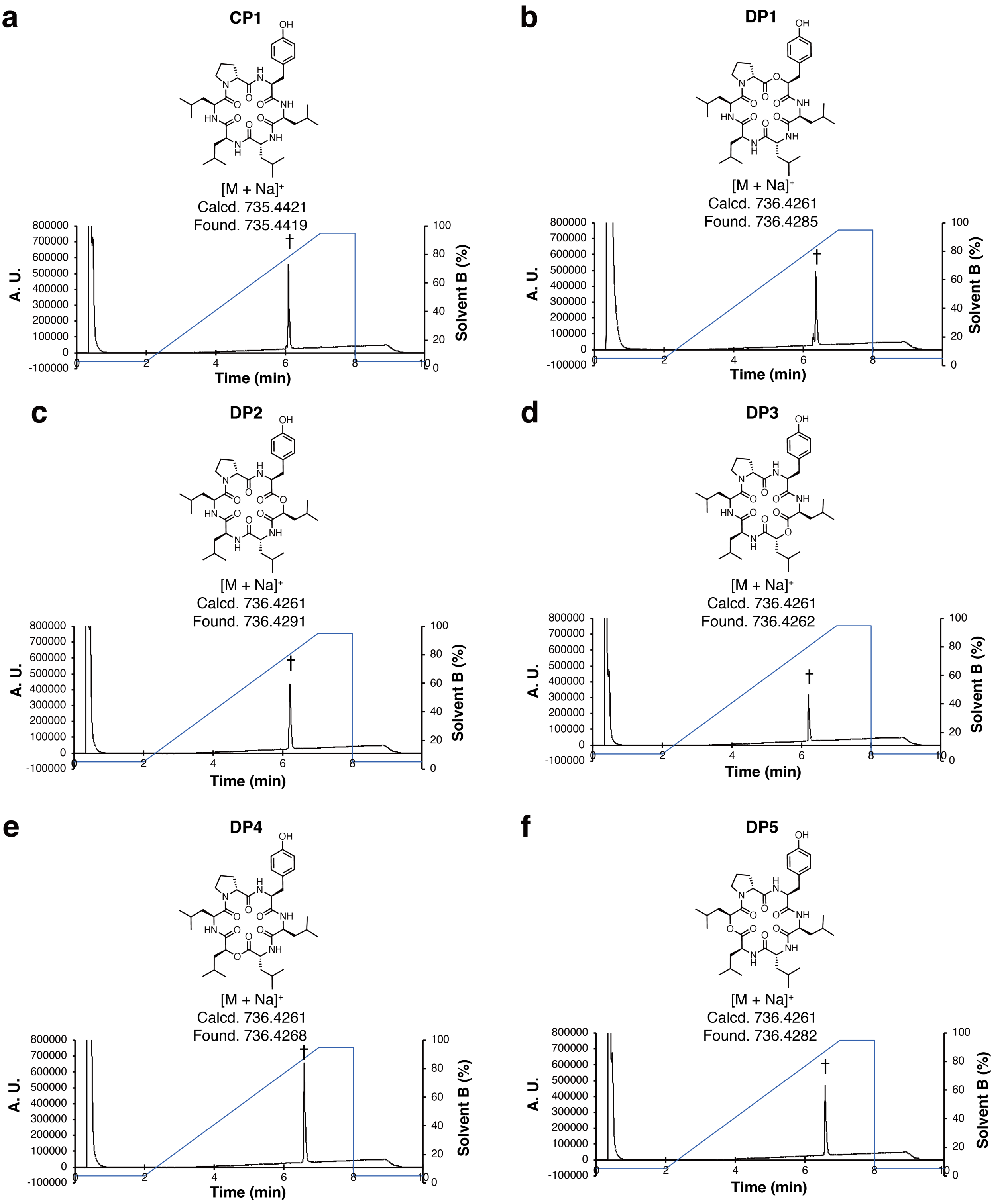

Supplementary Figure 54. UPLC chromatograms of CP1, DP1–5, and MP1–5

† denotes the fraction containing desired product. The calculated and found mass values of the desired compound are labeled under the chemical structure. (a) **CP1**, (b–f) **DP1**–**DP5**.

[cont.]

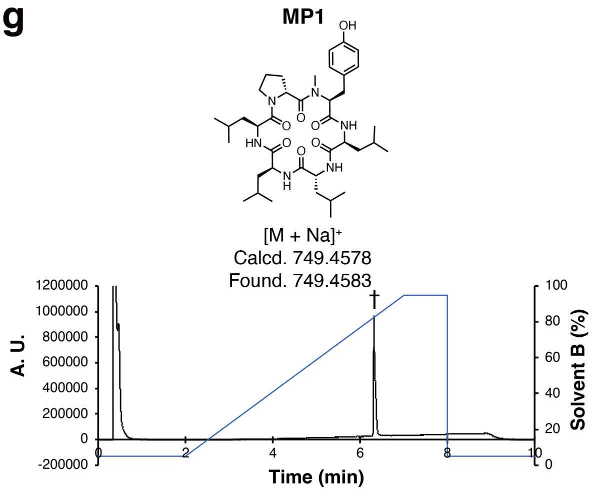

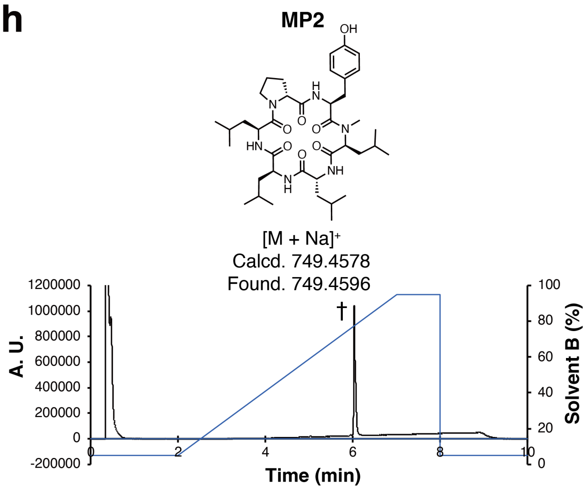

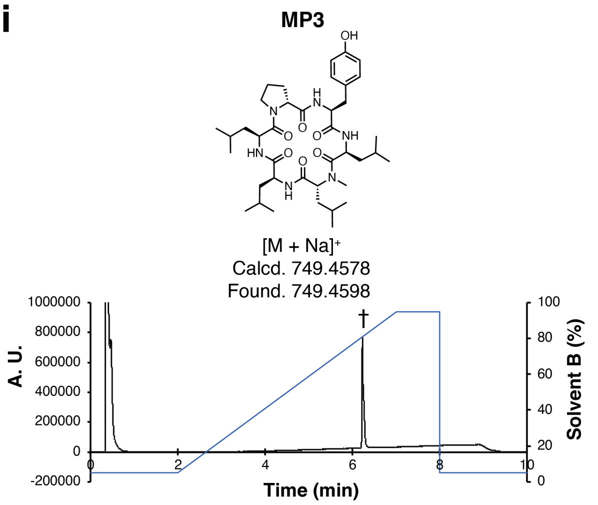

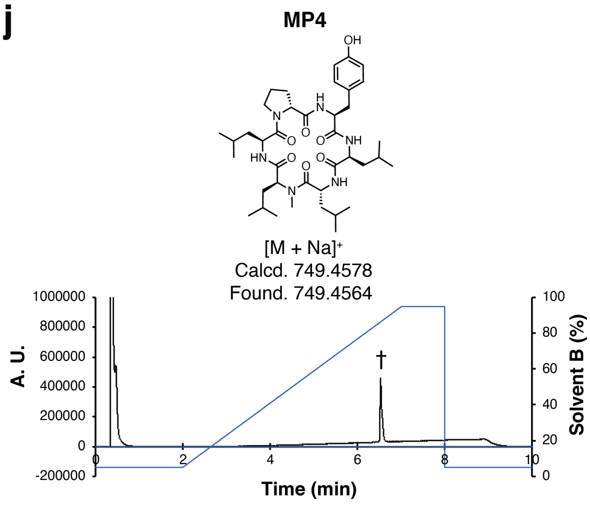

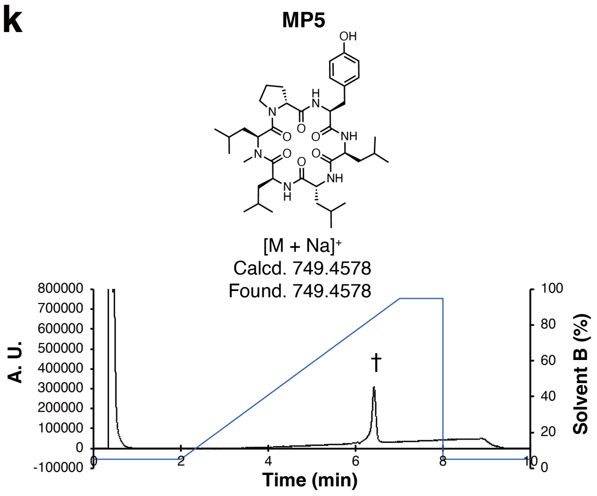

UPLC chromatograms of (g) **MP1**, (h) **MP2**, (i) **MP3**, (j) **MP4**, and (k) **MP5**.

[cont. from p67]

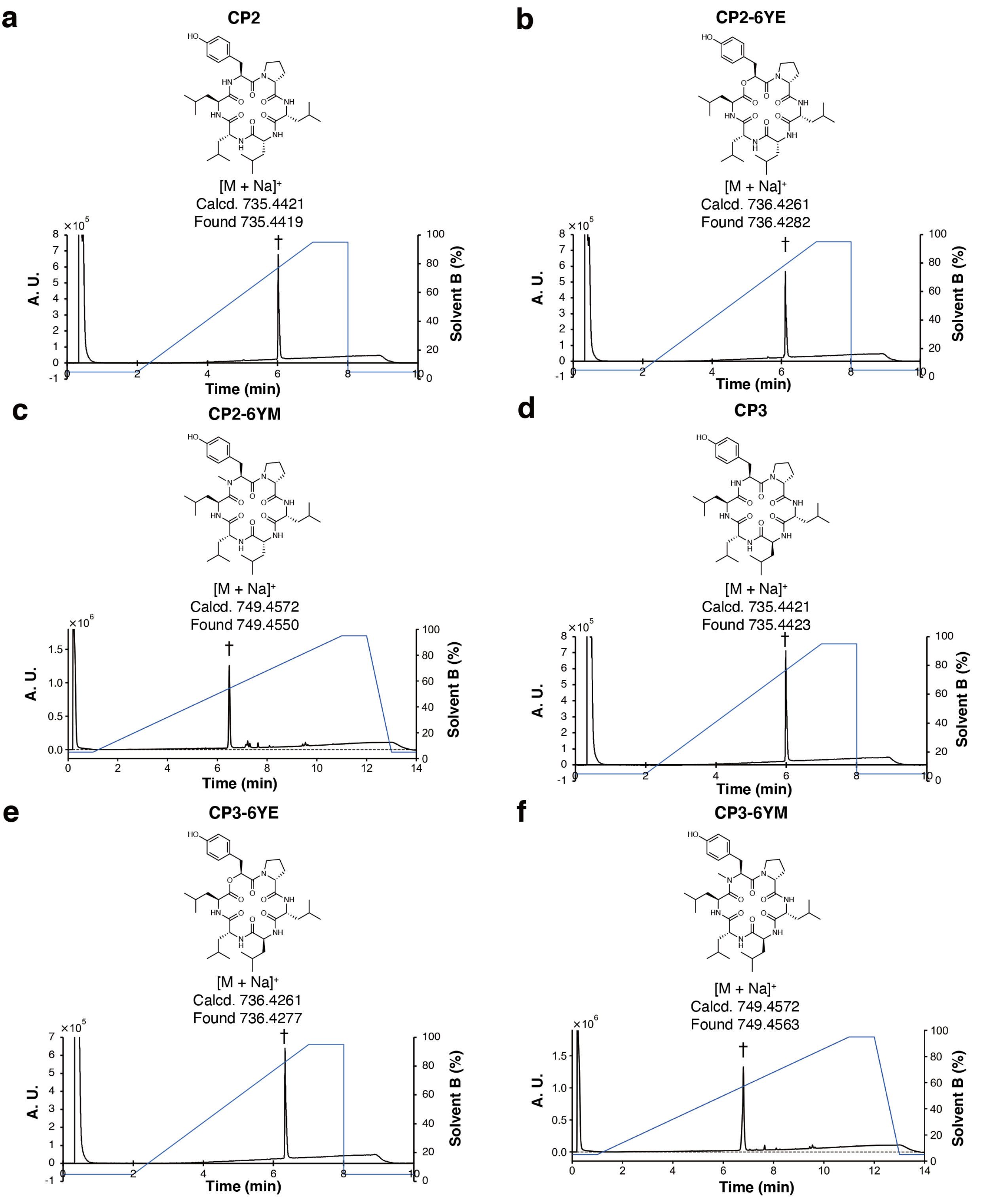

Supplementary Figure 55. UPLC chromatograms of CP2–6 and their N-methylated peptide and depsipeptide derivatives

† denotes the fraction containing desired product. The calculated and found mass values of the desired compound are labeled under the chemical structure. (a) **CP2**, (b) **CP2-6YE**, (c) **CP2-6YM**, (d) **CP3**, (e) **CP3-6YE**, (f) **CP3-6YM**. [cont.]

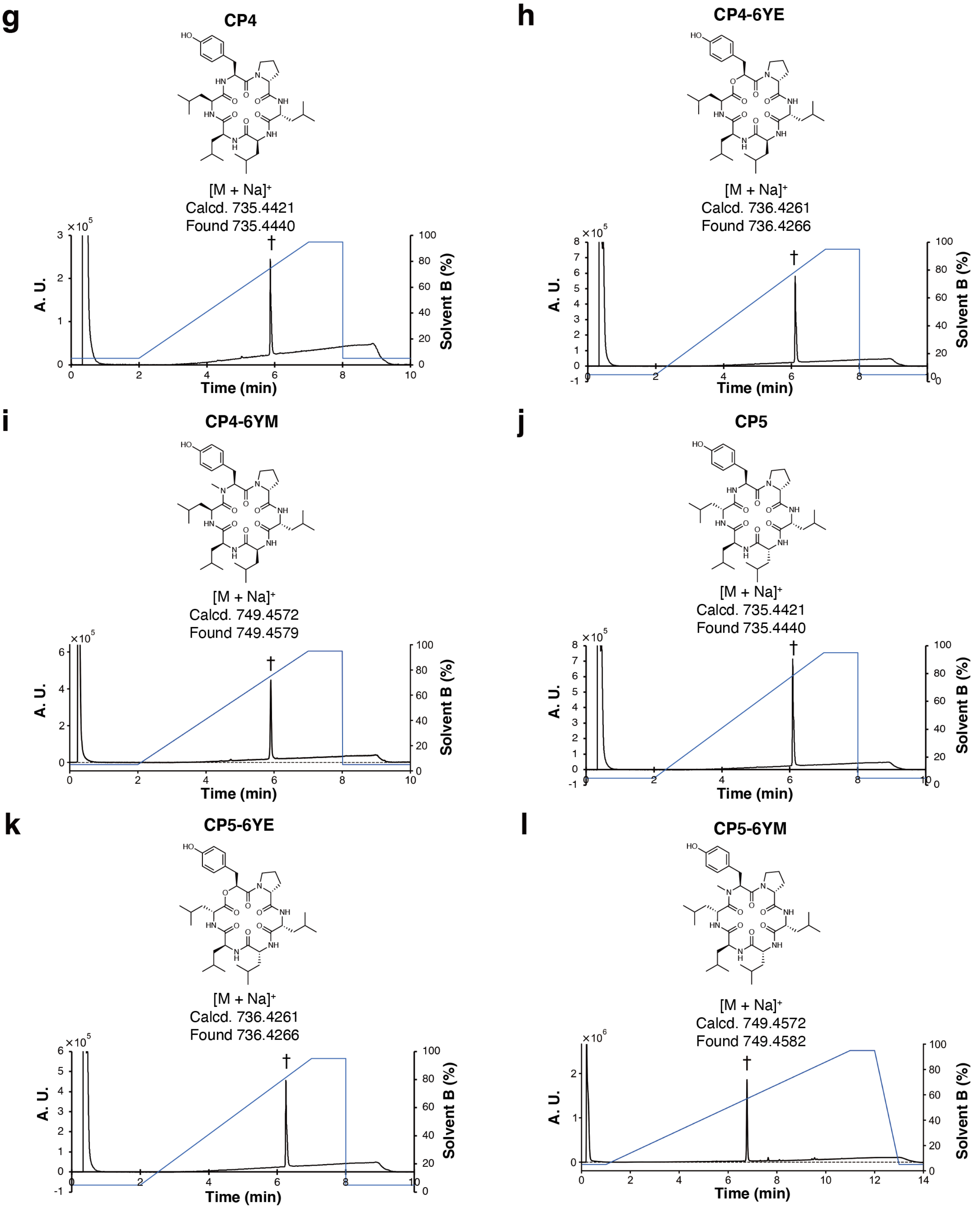

UPLC chromatograms of (g) **CP4**, (h) **CP4-6YE**, (i) **CP4-6YM**, (j) **CP5**, (k) **CP5-6YE**, and (l) **CP5-6YM**.

[cont.]

m

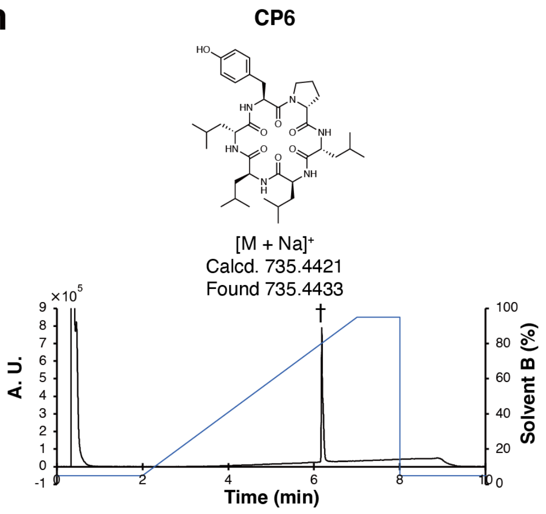

n

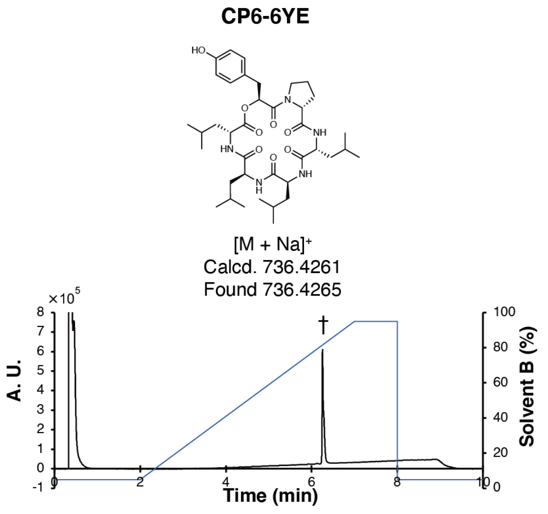

o

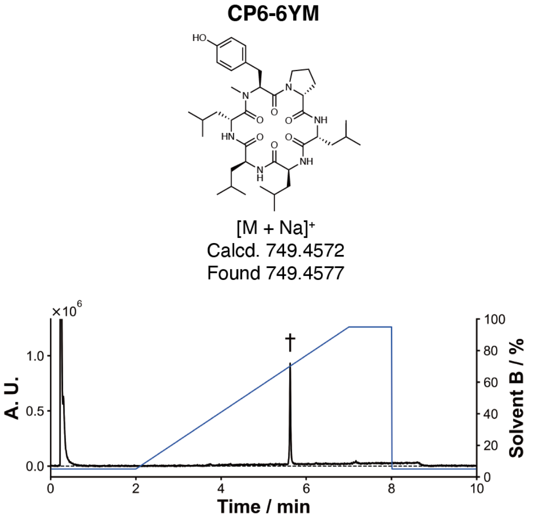

UPLC chromatograms of (m) **CP6**, (n) **CP6-6YE**, and (o) **CP6-6YM**.

[cont. from p69]

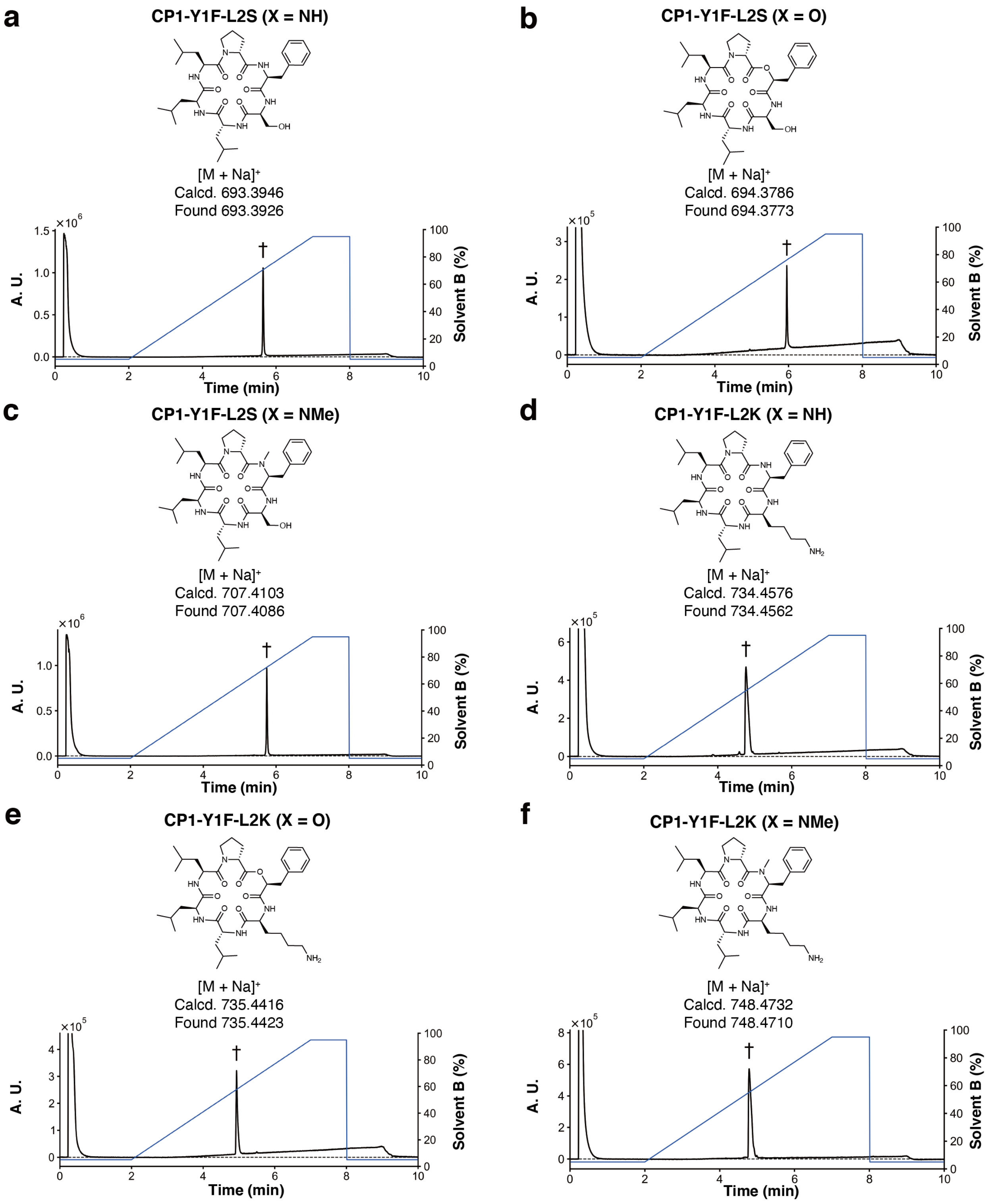

Supplementary Figure 56. UPLC chromatograms of CP1-Y1F-L2S/K and their derivatives

UPLC chromatograms of (a) **CP1-Y1F-L2S (X = NH)**, (b) **CP1-Y1F-L2S (X = O)**, (c) **CP1-Y1F-L2S (X = NMe)**, (d) **CP1-Y1F-L2K (X = NH)**, (e) **CP1-Y1F-L2K (X = O)**, and (f) **CP1-Y1F-L2S (X = NMe)**. † denotes the fraction containing desired product. The calculated and found mass values of the desired compound are labeled under the chemical structure.

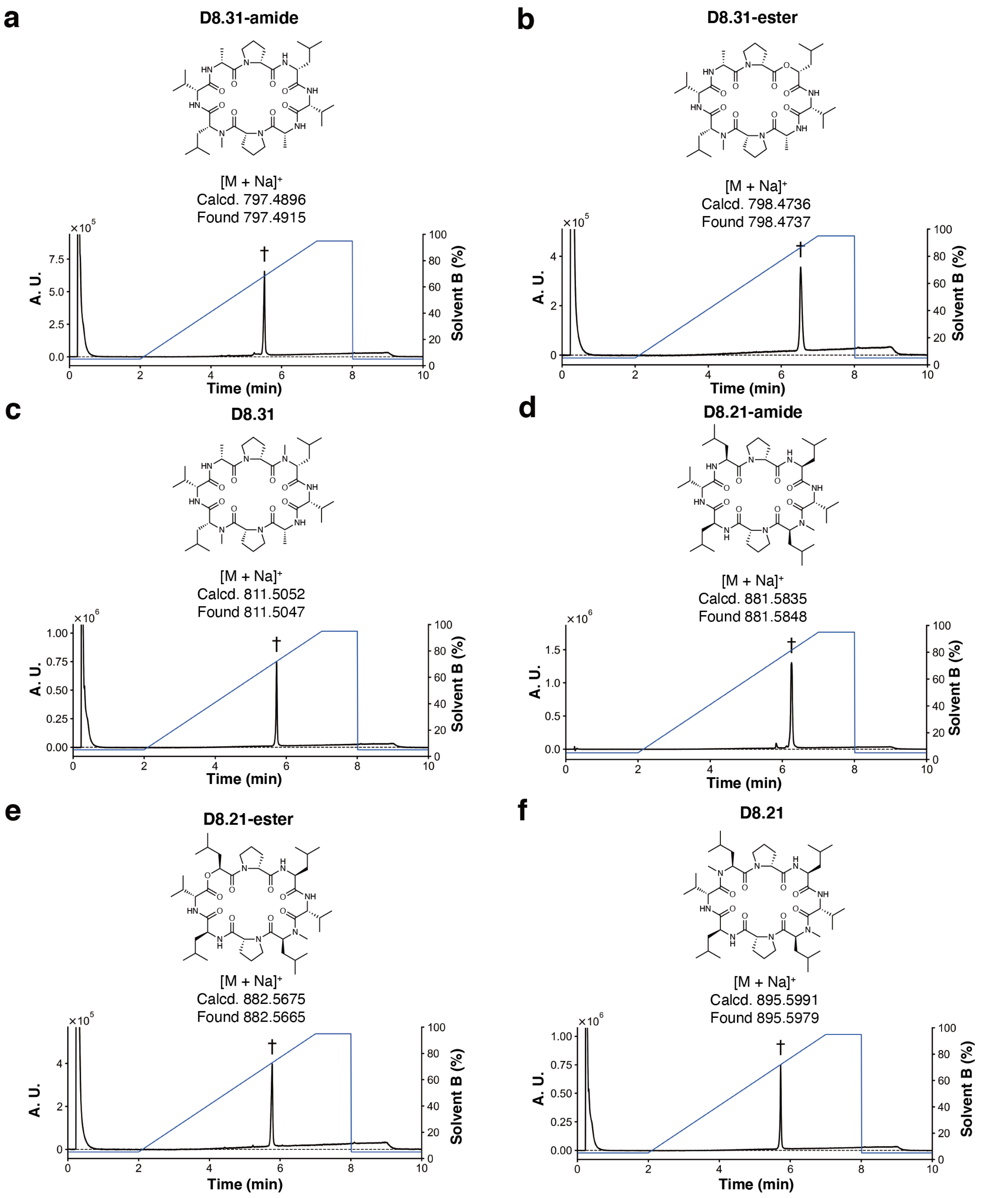

Supplementary Figure 57. UPLC chromatograms of D8.31/D8.21/D9.16 and their derivatives

† denotes the fraction containing desired product. The calculated and found mass values of the desired compound are labeled under the chemical structured. (a) **D8.31-amide**, (b) **D8.31-ester**, (c) **D8.31**, (d) **D8.21-amide**, (e) **D8.21-ester**, (f) **D8.21**.

[cont.]

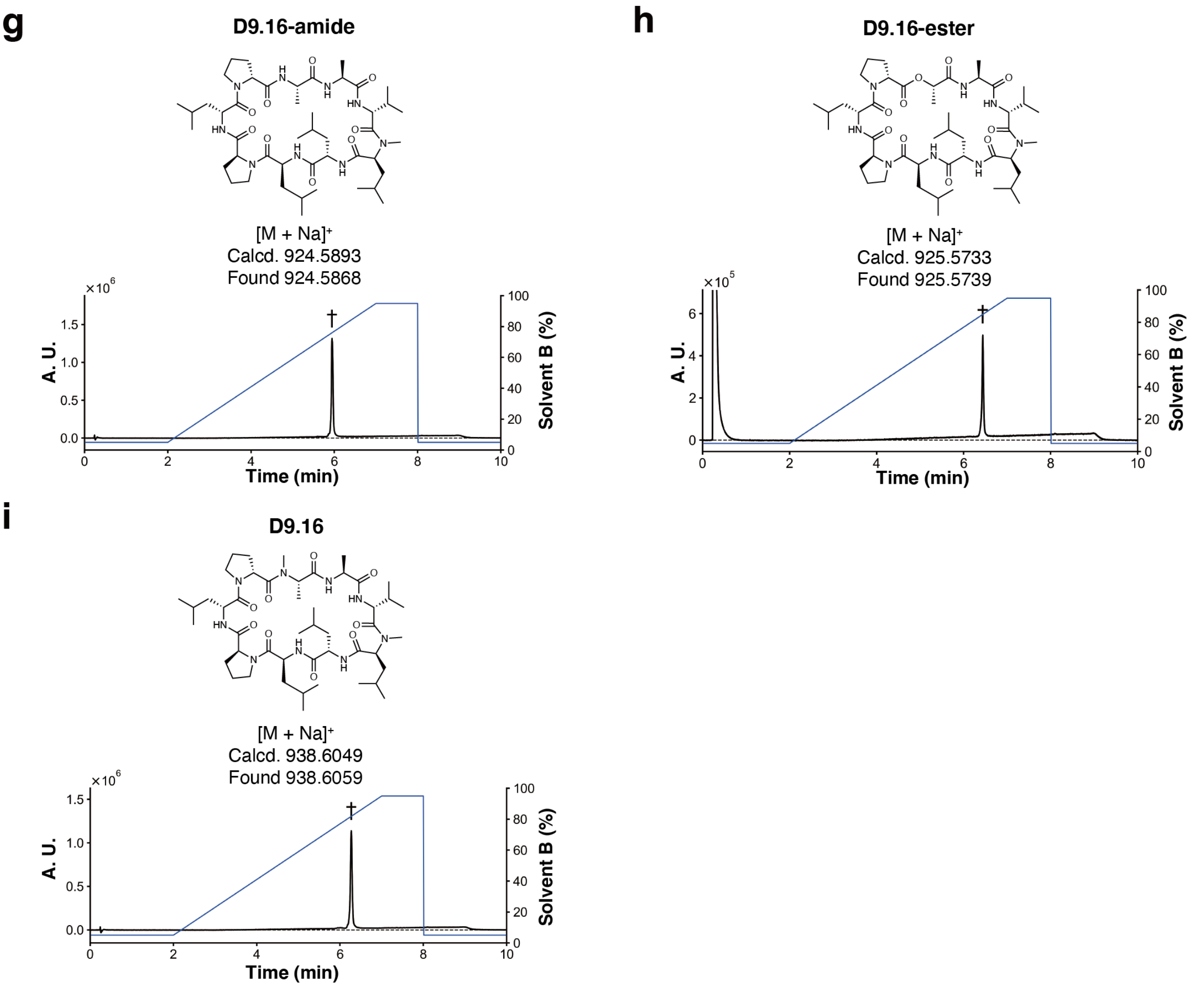

UPLC chromatograms of (g) **D9.16-amide**, (h) **D9.16-ester**, and (i) **D9.16**.

[cont. from p73]

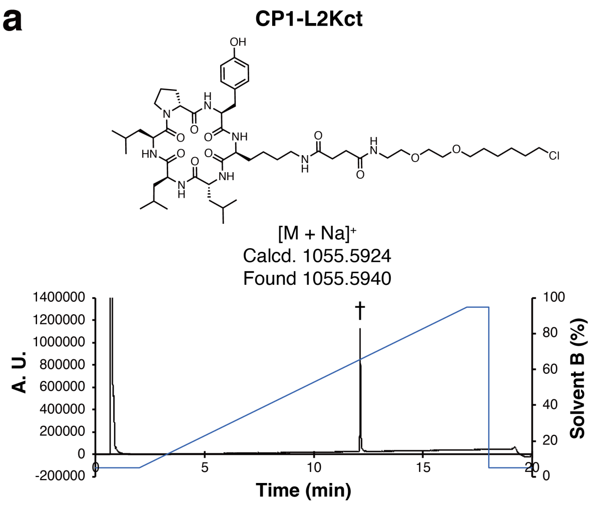

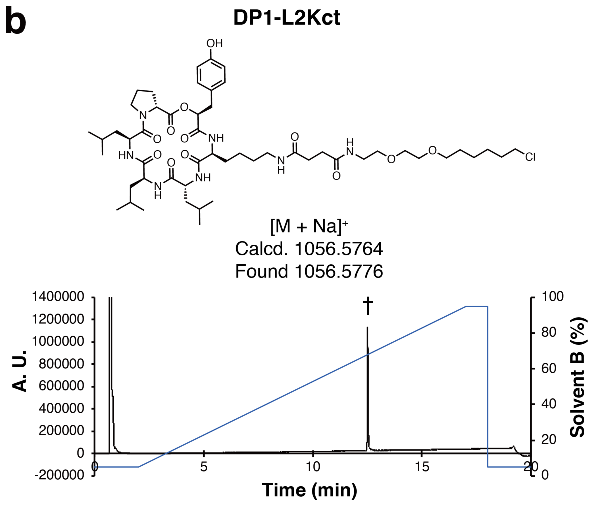

Supplementary Figure 58. UPLC chromatograms of CP1-L2Kct, DP1-L2Kct, and MP1-L2Kct

UPLC chromatograms of (a) **CP1-L2Kct** and (b) **DP1-L2Kct**. † denotes the fraction containing desired product. The calculated and found mass values of the desired compound are labeled under the chemical structure.

DP1L-NH₂

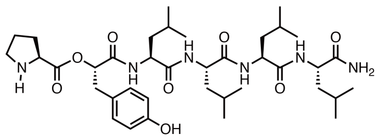

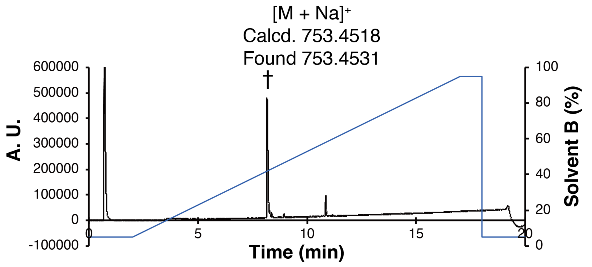

Supplementary Figure 59. UPLC chromatograms of DP1L-NH$_2$

UPLC chromatogram of **DP1L-NH$_2$**. † denotes the fraction containing desired product. The calculated and found mass values of the desired compound are labeled under the chemical structure.

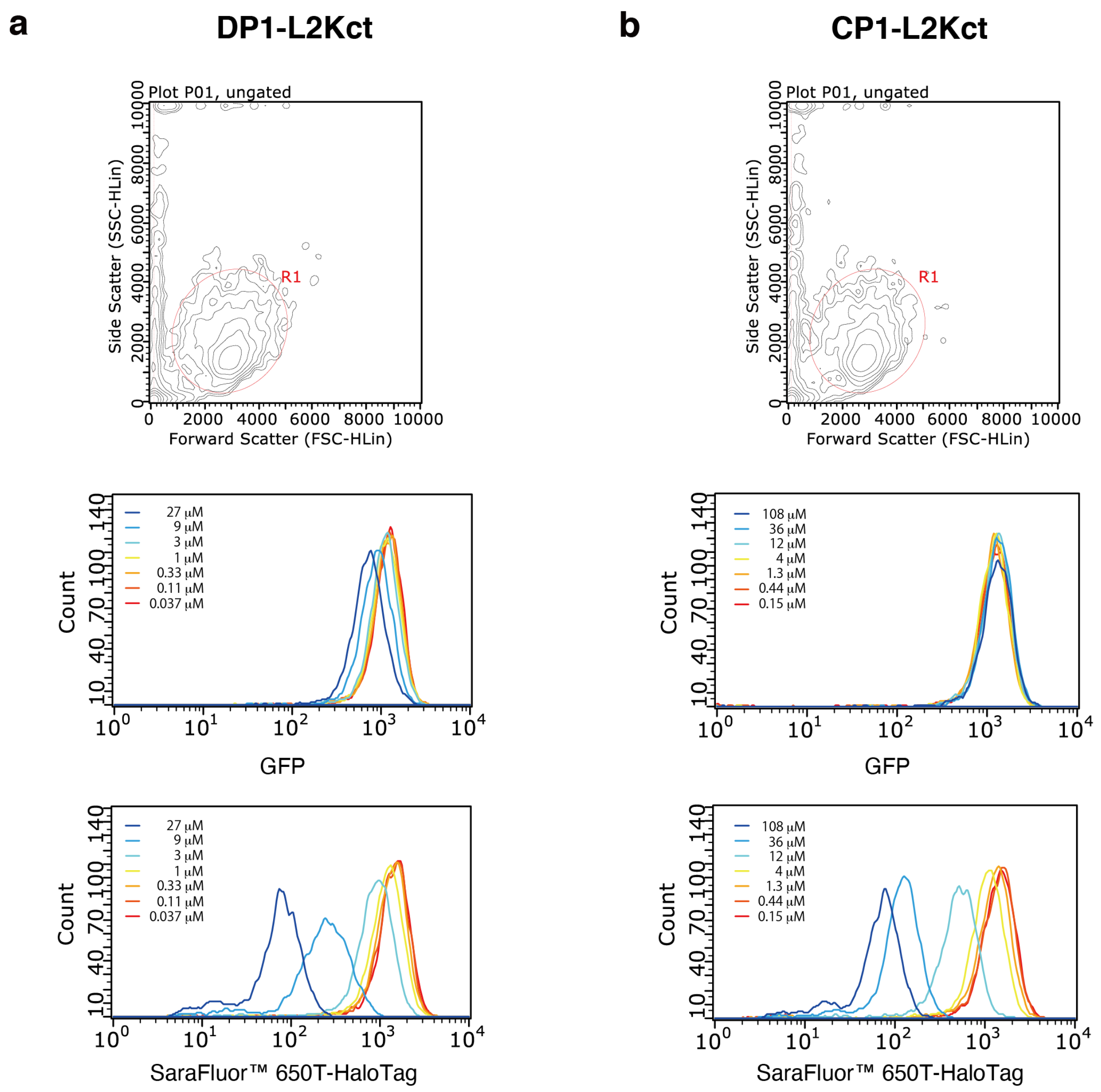

Supplementary Figure 60. Histograms of flow cytometric analysis of CAPA

Representative flow cytometry results of CAPA. The result of one experiment out of triplicate is shown. (a) **DP1-L2Kct**. (b) **CP1-L2Kct**. (Top) A representative FSC/SSC plot of ungated cells (27 μM and 108 μM peptide for **DP1-L2Kct** and **CP1-L2Kct**, respectively). The cells in the R1 region were gated and analyzed for their fluorescences. (Middle) Histograms based on green fluorescence from the gated cells. (Bottom) Histograms based on SaraFluor 650T from the gated cells.

Supplementary Table 1. Permeability values (P_e), ALogP, UPLC retention time, and log $D_{dec/w}$ of P1–3, D1–3, and M1–3.

Amide

Name	(Xaa_1, Xaa_2)	P_e (× 10^{-6} cm/s)	ALogP	UPLC retention time (min)	log $D_{dec/w}$
**P1**	(Phe, Phe)	0.07	1.34	7.67	–2.75 ± 0.01
**P2**	(Leu, Phe)	0.04	1.01	7.44	–3.24 ± 0.01
**P3**	(Leu, Leu)	0.01	0.69	6.84	–3.57 ± 0.03

Amide-to-ester substitution

Name	(Xaa_1, Xaa_2)	P_e (× 10^{-6} cm/s)	ALogP	UPLC retention time (min)	log $D_{dec/w}$
**D1**	(Phe, Phe)	1.2	1.98	9.07	–1.17 ± 0.01
**D2**	(Leu, Phe)	0.4	1.66	8.63	–2.0 ± 0.1
**D3**	(Leu, Leu)	0.2	1.33	8.25	–2.142 ± 0.001

Backbone *N*-methylation

Name	(Xaa_1, Xaa_2)	P_e (× 10^{-6} cm/s)	ALogP	UPLC retention time (min)	log $D_{dec/w}$
**M1**	(Phe, Phe)	0.3	1.54	8.74	–1.24 ± 0.01
**M2**	(Leu, Phe)	0.2	1.22	8.36	–1.62 ± 0.02
**M3**	(Leu, Leu)	0.1	0.89	7.63	–2.13 ± 0.02

Supplementary Table 2. Evaluation of the effect of efflux transporters on permeability of DP2 on Caco-2 assay.

	P_e (apical-to-basolateral) ($\times 10^{-6}$ cm/s)	P_e (basolateral-to-apical) ($\times 10^{-6}$ cm/s)	Efflux ratio
Inhibitor (–)	1.5 ± 0.4	40.8 ± 11.0	27.2
Inhibitor (+)*	14.6 ± 3.5	15.3 ± 1.1	1.05

*Caco-2 cells were preincubated with 50 μM quinidine, 20 μM sulfasalazine, and 30 μM benzbromarone before the assay. The Caco-2 assay was conducted under the same conditions as described in the Methods section other than that the incubation time was changed from 3 h to 2 h.

Supplementary Table 3. Amide temperature coefficients of CP1, DP1, and MP1.

(ppb/K)

Residue	**CP1**	**DP1**	**MP1**
Tyr-1	–3.3	(ester)	(*N*-methylamide)
Leu-2	-1.8	-0.6	-1.7
^{D}Leu-3	-1.7	-1.3	-5.3
Leu-4	–2.9	-3.7	-4.1
Leu-5	-2.2	-1.4	-3.3

Supplementary Table 4. Water solubility and mouse plasma stability

compounds	water solubility (μM)	mouse plasma stability (%)
CP1	110 ± 9	82 ± 10
DP1	25 ± 1	81 ± 8
DP2	56 ± 4	85 ± 6
DP3	56 ± 6	84 ± 12
DP4	5.5 ± 0.6	89 ± 22
DP5	5.2 ± 0.5	82 ± 10
MP1	67 ± 8	108 ± 10
MP2	91 ± 6	106 ± 7
MP3	82 ± 9	113 ± 17
MP4	42 ± 4	105 ± 2
MP5	4.0 ± 0.3	120 ± 13
Cyclosporin A	6 ± 1	-
DP1L-NH_2	-	21 ± 3

Supplementary Table 5. NOE summary of CP1 in $CDCl_3$

F1		F2		
Resdiue No.	Atom name	Resdiue No.	Atom name	Distance (Å)
1	HA	1	HD#	1.8 – 3.5
1	HA	1	HN	1.8 – 3.5
1	HA	2	HN	1.8 – 5
1	HA	3	HN	1.8 – 3.5
1	HB1	1	HA	1.8 – 2.7
1	HB1	1	HD#	1.8 – 2.7
1	HB1	1	HN	1.8 – 5
1	HB1	2	HN	1.8 – 5
1	HB2	1	HA	1.8 – 5
1	HB2	1	HD#	1.8 – 2.7
1	HB2	1	HN	1.8 – 3.5
1	HB2	2	HN	1.8 – 5
1	HD#	2	HN	1.8 – 5
1	HE#	1	HD#	1.8 – 2.7
1	HN	1	HD#	1.8 – 3.5
1	HN	2	HN	1.8 – 3.5
2	HA	2	HN	1.8 – 3.5
2	HA	3	HN	1.8 – 3.5
2	HB1	2	HA	1.8 – 5
2	HB1	2	HN	1.8 – 3.5
2	HB1	5	HN	1.8 – 5
2	HB2	2	HA	1.8 – 3.5
2	HB2	2	HN	1.8 – 5
2	HB2	5	HN	1.8 – 5
2	HD2#	1	HD#	1.8 – 3.5
2	HD2#	1	HE#	1.8 – 3.5
2	HD2#	2	HA	1.8 – 2.7
2	HD2#	2	HN	1.8 – 5
2	HG	2	HA	1.8 – 5
2	HG	2	HN	1.8 – 3.5
3	HA	3	HN	1.8 – 3.5
3	HA	4	HN	1.8 – 2.7
3	HA	5	HN	1.8 – 5
3	HB#	3	HA	1.8 – 3.5
3	HB#	3	HN	1.8 – 3.5
3	HD1#	3	HA	1.8 – 2.7
3	HD1#	3	HN	1.8 – 5
3	HD2#	3	HA	1.8 – 3.5
3	HD2#	3	HN	1.8 – 5
3	HG	3	HN	1.8 – 3.5
3	HN	2	HN	1.8 – 2.7
4	HA	4	HN	1.8 – 3.5
4	HA	5	HN	1.8 – 5

F1		F2		
Resdiue No.	Atom name	Resdiue No.	Atom name	Distance (Å)
4	HB1	4	HA	1.8 – 5
4	HB1	4	HN	1.8 – 3.5
4	HB2	4	HA	1.8 – 2.7
4	HB2	4	HN	1.8 – 5
4	HB2	5	HN	1.8 – 5
4	HD1#	4	HA	1.8 – 2.7
4	HD1#	4	HN	1.8 – 5
4	HD2#	4	HA	1.8 – 3.5
4	HD2#	4	HN	1.8 – 5
4	HG	4	HA	1.8 – 5
4	HG	4	HN	1.8 – 3.5
4	HN	3	HN	1.8 – 5
5	HA	5	HN	1.8 – 5
5	HB1	5	HA	1.8 – 3.5
5	HB1	5	HN	1.8 – 5
5	HB2	5	HA	1.8 – 3.5
5	HB2	5	HN	1.8 – 5
5	HB2	0	HD1	1.8 – 5
5	HD2#	5	HA	1.8 – 2.7
5	HD2#	5	HN	1.8 – 5
5	HD2#	0	HD1	1.8 – 3.5
5	HG	5	HA	1.8 – 3.5
5	HG	5	HN	1.8 – 5
5	HN	2	HN	1.8 – 3.5
5	HN	3	HN	1.8 – 5
5	HN	4	HN	1.8 – 3.5
6	HA	1	HN	1.8 – 2.7
6	HA	2	HN	1.8 – 5
6	HB1	6	HA	1.8 – 5
6	HB1	6	HD2	1.8 – 5
6	HB2	6	HA	1.8 – 2.7
6	HB2	6	HD1	1.8 – 5
6	HD1	5	HA	1.8 – 2.7
6	HD2	6	HA	1.8 – 5
6	HD3	6	HD2	1.8 – 2.7
6	HD2	5	HA	1.8 – 2.7
6	HD3	5	HN	1.8 – 5
6	HD4	6	HA	1.8 – 5
6	HG1	6	HA	1.8 – 5
6	HG1	6	HD1	1.8 – 3.5
6	HG1	6	HD2	1.8 – 5
6	HG2	6	HD1	1.8 – 3.5
6	HG2	6	HD2	1.8 – 3.5

- The table is described according to the distance restraints format in XPLOR-NIH (Schwieters, C. D., *et al. J. Magn. Reson.* **160**, 65–73 (2003).).
- HA, HB, HG, HD, and HE in Atom name indicate α-proton, β-protons, γ-proton, δ-protons, and ε-protons, respectively.
- When the Atom name is, for example, HB1, it indicates the two β-protons are treated distinctively during the structure calculations. The proton with a smaller chemical shift is given a smaller number.
- When the Atom name is, for example, HB#, it indicates the two β-protons are treated without distinction during the structure calculations.
- When the Atom name is, for example, HD1#, it indicates the two δ-carbons are treated distinctively but the three protons on the carbon are treated without distinction during the structure calculations.

Supplementary Table 6. $^3J_{HNCH}$ coupling values of CP1 in $CDCl_3$

Residue No.	Residue name	*J* (Hz)	ϕ (°)	Reason for N. D. (Not Determined)
1	Tyr	5.3	–69	
2	Leu	8.5	–93	
3	D-Leu	7.7	87	
4	Leu	5.2	–68	
5	Leu	8.4	–93	
6	D-Pro	N. D.	N. D.	pro (no amide NH)

- Structure calculations were conducted with the $\phi \pm 30°$ as restrictions.

Supplementary Table 7. NOE summary of CP1 in DMSO/water = 1/1

F1		F2				
Residue No.	Atom name	Residue No.	Atom name	Distance (Å)		
1	HE#	1	HD#	1.8	–	2.7
2	HB2	2	HA	1.8	–	2.7
2	HD1#	2	HA	1.8	–	2.7
3	HB#	3	HA	1.8	–	2.7
3	HD1#	3	HA	1.8	–	2.7
4	HD1#	4	HA	1.8	–	2.7
5	HB1	5	HA	1.8	–	2.7
5	HD1#	5	HA	1.8	–	2.7
6	HB2	6	HA	1.8	–	2.7
1	HA	1	HD#	1.8	–	3.5
1	HB1	1	HB2	1.8	–	3.5
1	HB1	1	HD#	1.8	–	3.5
1	HB2	1	HA	1.8	–	3.5
1	HB2	1	HD#	1.8	–	3.5
2	HD2#	2	HA	1.8	–	3.5
3	HD2#	3	HA	1.8	–	3.5
4	HB1	4	HA	1.8	–	3.5
4	HG	4	HA	1.8	–	3.5
6	HD1	5	HA	1.8	–	3.5
6	HD2	5	HA	1.8	–	3.5
1	HA	1	HE#	1.8	–	5
1	HB1	1	HA	1.8	–	5
2	HA	1	HD#	1.8	–	5
2	HD2#	1	HD#	1.8	–	5
2	HD2#	1	HE#	1.8	–	5
2	HG	1	HD#	1.8	–	5
2	HG	1	HE#	1.8	–	5
3	HD1#	1	HE#	1.8	–	5
4	HD1#	3	HB1	1.8	–	5
4	HD2#	4	HA	1.8	–	5
5	HD#	6	HD1	1.8	–	5
5	HD#	6	HD2	1.8	–	5
5	HG	6	HD1	1.8	–	5
6	HA	1	HD#	1.8	–	5
6	HB1	6	HD1	1.8	–	5
6	HB1	6	HD2	1.8	–	5
6	HB1	6	HA	1.8	–	5
6	HB1	1	HD#	1.8	–	5
6	HB1	1	HE#	1.8	–	5

F1		F2				
Residue No.	Atom name	Residue No.	Atom name	Distance (Å)		
6	HB2	6	HD1	1.8	–	5
6	HB2	6	HD2	1.8	–	5
6	HB2	1	HD#	1.8	–	5
6	HB2	1	HE#	1.8	–	5
6	HD1	6	HA	1.8	–	5
6	HG1	6	HA	1.8	–	5
6	HG1	6	HD1	1.8	–	5
6	HG1	6	HD2	1.8	–	5
6	HG2	6	HA	1.8	–	5
6	HG2	6	HD2	1.8	–	5
6	HG2	6	HD1	1.8	–	5
2	HA	3	HN	1.8	–	2.7
3	HA	4	HN	1.8	–	2.7
3	HB#	3	HN	1.8	–	2.7
4	HA	4	HN	1.8	–	2.7
6	HA	1	HN	1.8	–	2.7
1	HB1	1	HN	1.8	–	3.5
3	HA	3	HN	1.8	–	3.5
1	HA	2	HN	1.8	–	5
1	HA	1	HN	1.8	–	5
1	HD#	1	HN	1.8	–	5
2	HA	2	HN	1.8	–	5
2	HB1	2	HN	1.8	–	5
2	HB2	2	HN	1.8	–	5
2	HG	2	HN	1.8	–	5
2	HN	1	HN	1.8	–	5
2	HN	3	HN	1.8	–	5
3	HA	5	HN	1.8	–	5
4	HA	5	HN	1.8	–	5
4	HB1	4	HN	1.8	–	5
4	HB1	5	HN	1.8	–	5
4	HB2	4	HN	1.8	–	5
4	HD2#	4	HN	1.8	–	5
5	HA	5	HN	1.8	–	5
5	HB1	5	HN	1.8	–	5
5	HB2	5	HN	1.8	–	5
5	HN	4	HN	1.8	–	5
6	HA	2	HN	1.8	–	5

- The table format is the same with that of Supplementary Table 5.

Supplementary Table 8. $^{3}J_{HNCH}$ coupling values of CP1 in DMSO/water = 1/1

Residue No.	Residue name	*J* (Hz)	ϕ (°)	Reason for N. D. (Not Determined)
1	Tyr	7.7	–87	
2	Leu	8.4	–93	
3	D-Leu	6.8	80	
4	Leu	7.3	–84	
5	Leu	9.2	–101	
6	D-Pro	N. D.	N. D.	pro (no amide NH)

- Structure calculations were conducted with the $\phi \pm 30°$ as restrictions.

Supplementary Table 9. NOE summary of DP1 in $CDCl_3$

F1 Residue No.	F1 Atom name	F2 Residue No.	F2 Atom name	Distance (Å)	F1 Residue No.	F1 Atom name	F2 Residue No.	F2 Atom name	Distance (Å)
1	HA	3	HN	1.8 – 5	6	HB2	1	HD1	1.8 – 5
1	HB1	1	HD2	1.8 – 5	6	HD1#	6	HN	1.8 – 5
1	HB1	1	HD1	1.8 – 5	6	HD1#	1	HD1	1.8 – 5
1	HB1	1	HA	1.8 – 5	6	HD2#	6	HN	1.8 – 5
1	HB2	1	HD1	1.8 – 5	6	HG	6	HN	1.8 – 5
1	HB2	1	HD2	1.8 – 5	6	HG	6	HA	1.8 – 5
1	HD2	6	HN	1.8 – 5	6	HG	1	HD1	1.8 – 5
1	HG1	1	HD2	1.8 – 5	6	HN	4	HN	1.8 – 5
1	HG2	1	HD1	1.8 – 5	6	HN	3	HN	1.8 – 5
2	HA	3	HN	1.8 – 5	1	HG1	1	HD1	1.8 – 3.5
2	HA	4	HN	1.8 – 5	1	HG2	1	HD2	1.8 – 3.5
2	HB1	3	HN	1.8 – 5	2	HA	2	HD#	1.8 – 3.5
2	HB2	3	HN	1.8 – 5	2	HB2	2	HD#	1.8 – 3.5
2	HB2	2	HA	1.8 – 5	3	HA	4	HN	1.8 – 3.5
2	HD#	3	HN	1.8 – 5	3	HB1	3	HN	1.8 – 3.5
2	HE#	2	HD#	1.8 – 5	4	HA	4	HN	1.8 – 3.5
3	HA	3	HN	1.8 – 5	4	HB1	4	HN	1.8 – 3.5
3	HB1	3	HA	1.8 – 5	4	HB1	4	HA	1.8 – 3.5
3	HB2	3	HN	1.8 – 5	4	HB2	4	HN	1.8 – 3.5
3	HB2	6	HN	1.8 – 5	4	HB2	4	HA	1.8 – 3.5
3	HD1#	3	HN	1.8 – 5	4	HD1#	4	HA	1.8 – 3.5
3	HD1#	2	HE#	1.8 – 5	4	HD2#	4	HA	1.8 – 3.5
3	HD1#	2	HD#	1.8 – 5	4	HN)	3	HN	1.8 – 3.5
3	HD2#	3	HN	1.8 – 5	5	HB1	5	HN	1.8 – 3.5
3	HD2#	2	HE#	1.8 – 5	5	HB2	5	HN	1.8 – 3.5
3	HD2#	3	HA	1.8 – 5	6	HB1	6	HN	1.8 – 3.5
3	HG	3	HN	1.8 – 5	6	HB1	6	HA	1.8 – 3.5
3	HG	3	HA	1.8 – 5	6	HB2	6	HA	1.8 – 3.5
4	HD1#	4	HN	1.8 – 5	6	HD2#	6	HA	1.8 – 3.5
4	HD2#	4	HN	1.8 – 5	6	HN	5	HN	1.8 – 3.5
4	HG	4	HN	1.8 – 5	1	HB2	1	HA	1.8 – 2.7
5	HA	6	HN	1.8 – 5	1	HD1	6	HA	1.8 – 2.7
5	HB2	6	HN	1.8 – 5	1	HD1	1	HD2	1.8 – 2.7
5	HD1#	5	HN	1.8 – 5	1	HD2	6	HA	1.8 – 2.7
5	HD2#	5	HN	1.8 – 5	2	HB1	2	HD#	1.8 – 2.7
5	HD2#	5	HA	1.8 – 5	2	HB1	2	HA	1.8 – 2.7
5	HN	4	HN	1.8 – 5	3	HB2	3	HA	1.8 – 2.7
6	HA	6	HN	1.8 – 5	3	HD1#	3	HA	1.8 – 2.7
6	HB1	1	HD1	1.8 – 5	4	HA	5	HN	1.8 – 2.7
6	HB2	6	HN	1.8 – 5	5	HD1#	5	HA	1.8 – 2.7

- The table format is the same with that of Supplementary Table 5.

Supplementary Table 10. $^3J_{HNCH}$ coupling values of DP1 in $CDCl_3$

Residue No.	Residue name	*J* (Hz)	ϕ (°)	Reason for N. D. (Not Determined)
1	Tyr	N. D.	N. D.	ester (no amide NH)
2	Leu	8.5	–93	
3	D-Leu	7.4	84	
4	Leu	6.6	–78	
5	Leu	8.6	–95	
6	D-Pro	N. D.	N. D.	pro (no amide NH)

- Structure calculations were conducted with the $\phi \pm 30°$ as restrictions.

Supplementary Table 11. NOE summary of DP1 in DMSO/water = 1/1

F1		F2					F1		F2				
Residue No.	Atom name	Residue No.	Atom name	Distance (Å)			Residue No.	Atom name	Residue No.	Atom name	Distance (Å)		
1	HA	1	HB2	1.8	–	2.7	6	HA	6	HG2	1.8	–	5
1	HB1	1	HD#	1.8	–	2.7	6	HA	1	HD#	1.8	–	5
1	HB2	1	HB1	1.8	–	2.7	6	HA	1	HA	1.8	–	5
1	HE#	1	HD#	1.8	–	2.7	6	HD1	6	HB2	1.8	–	5
2	HA	2	HD1#	1.8	–	2.7	6	HD1	6	HG2	1.8	–	5
4	HA	4	HD1#	1.8	–	2.7	6	HD1	5	HB1	1.8	–	5
5	HA	5	HD#	1.8	–	2.7	6	HD2	6	HG1	1.8	–	5
5	HA	5	HB1	1.8	–	2.7	6	HD2	6	HB1	1.8	–	5
1	HA	1	HB1	1.8	–	3.5	1	HA	2	HN	1.8	–	5
1	HB2	1	HD#	1.8	–	3.5	2	HA	2	HN	1.8	–	5
2	HA	2	HD2#	1.8	–	3.5	2	HA	3	HN	1.8	–	2.7
2	HA	2	HB2	1.8	–	3.5	2	HB1	2	HN	1.8	–	5
2	HD1#	1	HE#	1.8	–	3.5	2	HB2	2	HN	1.8	–	5
3	HA	3	HD2#	1.8	–	3.5	2	HD2#	2	HN	1.8	–	5
3	HA	3	HD1#	1.8	–	3.5	2	HD2#	3	HN	1.8	–	5
3	HA	3	HB2	1.8	–	3.5	2	HG	2	HN	1.8	–	5
3	HA	3	HB1	1.8	–	3.5	2	HN	3	HN	1.8	–	5
4	HA	4	HB#	1.8	–	3.5	3	HA	3	HN	1.8	–	5
5	HA	6	HD2	1.8	–	3.5	3	HA	4	HN	1.8	–	2.7
5	HA	6	HD1	1.8	–	3.5	3	HA	5	HN	1.8	–	5
6	HA	6	HB2	1.8	–	3.5	3	HB1	3	HN	1.8	–	3.5
6	HA	6	HB1	1.8	–	3.5	3	HB2	3	HN	1.8	–	3.5
6	HD1	6	HG1	1.8	–	3.5	4	HA	4	HN	1.8	–	5
6	HD2	6	HG2	1.8	–	3.5	4	HA	5	HN	1.8	–	5
1	HA	1	HD#	1.8	–	5	4	HB#	5	HN	1.8	–	5
2	HA	2	HG	1.8	–	5	4	HB#	4	HN	1.8	–	3.5
2	HA	2	HB1	1.8	–	5	4	HD1#	4	HN	1.8	–	5
2	HD1#	1	HD#	1.8	–	5	4	HD2#	4	HN	1.8	–	5
2	HD2#	1	HE#	1.8	–	5	4	HG	4	HN	1.8	–	5
4	HA	4	HG	1.8	–	5	5	HA	5	HN	1.8	–	5
4	HA	4	HD2#	1.8	–	5	5	HB1	5	HN	1.8	–	3.5
5	HA	5	HB2	1.8	–	5	5	HB2	5	HN	1.8	–	3.5
6	HA	6	HD1	1.8	–	5	5	HD#	5	HN	1.8	–	5
6	HA	6	HG1	1.8	–	5	5	HN	4	HN	1.8	–	3.5

- The table format is the same with that of Supplementary Table 5.

Supplementary Table 12. $^3J_{HNCH}$ coupling values of DP1 in DMSO/water = 1/1

Residue No.	Residue name	*J* (Hz)	ϕ (°)	Reason for N. D. (Not Determined)
1	Tyr	N. D.	N. D.	ester (no amide NH)
2	Leu	8.3	–92	
3	D-Leu	7.0	81	
4	Leu	7.2	–83	
5	Leu	9.3	–102	
6	D-Pro	N. D.	N. D.	pro (no amide NH)

- Structure calculations were conducted with the $\phi \pm 30°$ as restrictions.

Supplementary Table 13. NOE summary of MP1 in $CDCl_3$

F1		F2		Distance (Å)
Residue No.	Atom name	Residue No.	Atom name	
1	HA	2	HN	1.8 – 5
1	HA	3	HN	1.8 – 5
1	HA	1	HD#	1.8 – 3
1	HB1	1	HD#	1.8 – 3
1	HB1	1	HB2	1.8 – 3
1	HB1	2	HN	1.8 – 5
1	HB1	6	HB2	1.8 – 5
1	HB2	1	HD#	1.8 – 4
1	HB2	1	HA	1.8 – 4
1	HE#	1	HD#	1.8 – 3
1	HX#	1	HD#	1.8 – 4
1	HX#	2	HN	1.8 – 4
1	HX#	6	HA	1.8 – 4
1	HX#	1	HA	1.8 – 5
1	HX#	2	HG	1.8 – 4
1	HX#	1	HB1	1.8 – 3
2	HA	3	HN	1.8 – 4
2	HA	2	HN	1.8 – 5
2	HB1	2	HN	1.8 – 4
2	HB1	2	HA	1.8 – 4
2	HB2	2	HN	1.8 – 5
2	HB2	2	HA	1.8 – 5
2	HD#	2	HN	1.8 – 5
2	HD#	2	HA	1.8 – 3
2	HD#	3	HN	1.8 – 5
2	HG	2	HN	1.8 – 5
2	HG	2	HA	1.8 – 5
2	HG	1	HX#	1.8 – 4
3	HA	3	HN	1.8 – 5
3	HB1	3	HN	1.8 – 4
3	HB1	3	HA	1.8 – 4
3	HB2	5	HN	1.8 – 5
3	HB2	3	HN	1.8 – 5
3	HD1#	3	HN	1.8 – 5
3	HD2#	3	HN	1.8 – 5
3	HN	2	HN	1.8 – 4
4	HA	4	HN	1.8 – 4
4	HA	5	HN	1.8 – 5
4	HB1	4	HN	1.8 – 5
4	HB1	4	HA	1.8 – 5
4	HB1	3	HB#	1.8 – 5
4	HB2	4	HN	1.8 – 5
4	HB2	4	HA	1.8 – 3
4	HD1#	4	HN	1.8 – 5
4	HD2#	4	HN	1.8 – 5
4	HG	4	HN	1.8 – 5
4	HG	4	HA	1.8 – 5
5	HA	5	HN	1.8 – 5
5	HB1	2	HN	1.8 – 5
5	HB1	5	HN	1.8 – 5
5	HB1	5	HA	1.8 – 3.5
5	HB1	6	HD1	1.8 – 5
5	HB2	2	HN	1.8 – 5
5	HB2	5	HN	1.8 – 5
5	HB2	5	HA	1.8 – 3.5
5	HB2	6	HD1	1.8 – 5
3	HB1	1	HA	1.8 – 5
5	HD1#	5	HA	1.8 – 3.5
5	HD1#	5	HN	1.8 – 5
5	HD1#	6	HD1	1.8 – 5
5	HD1#	6	HD2	1.8 – 5
5	HD2#	5	HA	1.8 – 3.5
5	HD2#	5	HN	1.8 – 5
5	HD2#	6	HD1	1.8 – 5
5	HG	5	HN	1.8 – 5
5	HG	5	HA	1.8 – 3.5
5	HN	2	HN	1.8 – 5
5	HN	3	HN	1.8 – 5
6	HA	2	HN	1.8 – 5
6	HB1	6	HA	1.8 – 5
6	HB1	6	HD2	1.8 – 5
6	HB1	6	HD1	1.8 – 5
6	HB1	1	HA	1.8 – 5
6	HB2	6	HA	1.8 – 2.7
6	HB2	6	HD1	1.8 – 5
6	HB2	6	HD2	1.8 – 5
6	HD1	5	HA	1.8 – 2.7
6	HD1	6	HD2	1.8 – 2.7
6	HD1	6	HA	1.8 – 5
6	HD2	5	HA	1.8 – 2.7
6	HD2	6	HA	1.8 – 5
6	HG1	6	HA	1.8 – 3.5
6	HG1	6	HD1	1.8 – 3.5
6	HG1	6	HD2	1.8 – 5
6	HG2	6	HA	1.8 – 5
6	HG2	6	HD2	1.8 – 3.5
6	HG2	6	HD1	1.8 – 5

- The table format is the same with that of Supplementary Table 5.
- HX in Atom name indicates *N*-methyl protons.

Supplementary Table 14. $^3J_{HNCH}$ coupling values of MP1 in $CDCl_3$

Residue No.	Residue name	*J* (Hz)	ϕ (°)	Reason for N. D. (Not Determined)
1	Tyr	N. D.	N. D.	*N*-methylamide (no amide NH)
2	Leu	9.3	–102	
3	D-Leu	7.5	85	
4	Leu	4	–77	
5	Leu	5	–90	
6	D-Pro	N. D.	N. D.	pro (no amide NH)

- Structure calculations were conducted with the $\phi \pm 30°$ as restrictions.

Supplementary Table 15. NOE summary of MP1 in DMSO/water = 1/1

F1		F2			F1		F2		
Residue No.	Atom type	Residue No.	Atom type	Distance (Å)	Residue No.	Atom type	Residue No.	Atom type	Distance (Å)
1	HB1	1	HB2	1.8 – 2.7	3	HB#	4	HA	1.8 – 5
1	HE#	1	HD#	1.8 – 2.7	4	HD2#	4	HA	1.8 – 5
3	HB#	3	HA	1.8 – 2.7	5	HB1	6	HD1	1.8 – 5
3	HD1#	3	HA	1.8 – 2.7	5	HD#	6	HD2	1.8 – 5
3	HD2#	3	HA	1.8 – 2.7	5	HD#	6	HD1	1.8 – 5
4	HB2	4	HA	1.8 – 2.7	5	HG	6	HD1	1.8 – 5
4	HD1#	4	HA	1.8 – 2.7	5	HG	5	HA	1.8 – 5
5	HD#	5	HA	1.8 – 2.7	6	HA	6	HB1	1.8 – 5
6	HA	1	HX#	1.8 – 2.7	6	HB1	6	HD2	1.8 – 5
1	HA	1	HD#	1.8 – 3.5	6	HB1	1	HX#	1.8 – 5
1	HB1	1	HX#	1.8 – 3.5	6	HB1	1	HD#	1.8 – 5
1	HB1	1	HD#	1.8 – 3.5	6	HB2	6	HD2	1.8 – 5
1	HB2	1	HA	1.8 – 3.5	6	HB2	1	HX#	1.8 – 5
1	HB2	1	HD#	1.8 – 3.5	6	HG2	6	HD1	1.8 – 5
1	HX#	1	HD#	1.8 – 3.5	1	HA	2	HN	1.8 – 3.5
2	HA	2	HB1	1.8 – 3.5	1	HX#	2	HN	1.8 – 5
2	HA	2	HD2#	1.8 – 3.5	2	HA	3	HN	1.8 – 3.5
2	HA	2	HD1#	1.8 – 3.5	2	HA	2	HN	1.8 – 5
4	HB1	4	HA	1.8 – 3.5	2	HB1	2	HN	1.8 – 5
4	HD1#	3	HB#	1.8 – 3.5	2	HB2	2	HN	1.8 – 5
5	HB1	5	HA	1.8 – 3.5	2	HG	2	HN	1.8 – 5
5	HB2	5	HA	1.8 – 3.5	3	HA	4	HN	1.8 – 2.7
6	HA	6	HB2	1.8 – 3.5	3	HA	3	HN	1.8 – 5
6	HB2	6	HD1	1.8 – 3.5	3	HA	5	HN	1.8 – 5
6	HD1	5	HA	1.8 – 3.5	3	HB#	3	HN	1.8 – 5
6	HD2	5	HA	1.8 – 3.5	4	HA	5	HN	1.8 – 5
6	HG2	6	HD2	1.8 – 3.5	4	HA	4	HN	1.8 – 5
1	HB1	1	HA	1.8 – 5	4	HB1	4	HN	1.8 – 3.5
1	HX#	1	HA	1.8 – 5	4	HB2	4	HN	1.8 – 3.5
2	HA	2	HB2	1.8 – 5	4	HD2#	4	HN	1.8 – 5
2	HA	2	HG	1.8 – 5	5	HA	5	HN	1.8 – 5
2	HB1	1	HX#	1.8 – 5	5	HB1	5	HN	1.8 – 3.5
2	HB2	1	HX#	1.8 – 5	5	HB2	5	HN	1.8 – 5
2	HD1#	1	HX#	1.8 – 5	5	HG	5	HN	1.8 – 5
2	HD2#	1	HX#	1.8 – 5	5	HN	2	HN	1.8 – 5
2	HG	1	HX#	1.8 – 5	5	HN	4	HN	1.8 – 5

- The table format is the same with that of Supplementary Table 13.

Supplementary Table 16. $^3J_{HNCH}$ coupling values of MP1 in DMSO/water = 1/1

Residue No.	Residue name	*J* (Hz)	ϕ (°)	Reason for N. D. (Not Determined)
1	Tyr	N. D.	N. D.	*N*-methylated amide (no amide NH)
2	Leu	8.2	–90	
3	D-Leu	4.3	62	
4	Leu	7.4	–84	
5	Leu	9.1	–99	
6	D-Pro	N. D.	N. D.	pro (no amide NH)

- Structure calculations were conducted with the ϕ ± 30° as restrictions.

Supplementary Table 17. NOE summary of DP2 in CDCl3

F1 Residue No.	F1 Atom name	F2 Residue No.	F2 Atom name	Distance (Å)	F1 Residue No.	F1 Atom name	F2 Residue No.	F2 Atom name	Distance (Å)
1	HA	3	HN	1.8 – 5	4	HB2	4	HN	1.8 – 5
1	HA	1	HN	1.8 – 3.5	4	HB2	4	HA	1.8 – 3.5
1	HA	1	HD#	1.8 – 3.5	4	HB2	5	HN	1.8 – 5
1	HB1	1	HD#	1.8 – 2.7	4	HD1#	4	HN	1.8 – 5
1	HB1	1	HA	1.8 – 3.5	4	HD1#	4	HA	1.8 – 2.7
1	HB1	1	HN	1.8 – 3.5	4	HD2#	4	HN	1.8 – 5
1	HB2	1	HA	1.8 – 3.5	4	HD2#	4	HA	1.8 – 5
1	HB2	1	HD#	1.8 – 2.7	4	HG	4	HN	1.8 – 3.5
1	HB2	1	HN	1.8 – 5	4	HG	4	HA	1.8 – 5
2	HA	3	HN	1.8 – 3.5	4	HG	5	HN	1.8 – 5
2	HB#	2	HA	1.8 – 5	4	HN	3	HN	1.8 – 5
2	HB#	4	HN	1.8 – 5	5	HA	5	HN	1.8 – 3.5
2	HB#	5	HN	1.8 – 3.5	5	HB1	5	HN	1.8 – 5
2	HD1#	2	HA	1.8 – 2.7	5	HB1	5	HA	1.8 – 3.5
2	HD1#	1	HD#	1.8 – 5	5	HB1	6	HD1	1.8 – 5
2	HD1#	1	HN	1.8 – 5	5	HB1	2	HB2	1.8 – 5
2	HD1#	1	HB2	1.8 – 5	5	HB2	5	HN	1.8 – 5
2	HD2#	5	HB1	1.8 – 5	5	HB2	5	HA	1.8 – 3.5
2	HD2#	2	HA	1.8 – 5	5	HB2	6	HD1	1.8 – 5
2	HD2#	3	HN	1.8 – 5	5	HD#	5	HN	1.8 – 5
2	HD2#	1	HD#	1.8 – 5	5	HD#	5	HA	1.8 – 2.7
2	HD2#	1	HN	1.8 – 5	5	HD#	6	HD1	1.8 – 5
2	HD2#	5	HN	1.8 – 5	5	HD#	6	HD2	1.8 – 5
2	HD2#	1	HB1	1.8 – 5	5	HG	5	HN	1.8 – 5
2	HD2#	1	HB2	1.8 – 5	5	HG	5	HA	1.8 – 5
2	HG	2	HA	1.8 – 5	5	HG	6	HD1	1.8 – 5
2	HG	1	HB1	1.8 – 5	5	HN	4	HN	1.8 – 3.5
2	HG	1	HB2	1.8 – 5	5	HN	3	HN	1.8 – 5
3	HA	3	HN	1.8 – 3.5	6	HA	3	HN	1.8 – 5
3	HA	4	HN	1.8 – 2.7	6	HA	1	HN	1.8 – 2.7
3	HB1	3	HN	1.8 – 5	6	HB1	6	HA	1.8 – 2.7
3	HB1	3	HA	1.8 – 5	6	HB1	6	HD1	1.8 – 5
3	HB2	3	HN	1.8 – 3.5	6	HB2	6	HA	1.8 – 3.5
3	HB2	3	HA	1.8 – 3.5	6	HB2	6	HD2	1.8 – 5
3	HD1#	3	HN	1.8 – 5	6	HD1	6	HA	1.8 – 5
3	HD1#	3	HA	1.8 – 2.7	6	HD1	5	HA	1.8 – 2.7
3	HD2#	3	HN	1.8 – 5	6	HD2	3	HN	1.8 – 5
3	HD2#	3	HA	1.8 – 3.5	6	HD2	6	HA	1.8 – 5
3	HD2#	1	HB1	1.8 – 5	6	HD2	5	HA	1.8 – 3.5
3	HG	3	HN	1.8 – 5	6	HD2	5	HN	1.8 – 5
3	HG	3	HA	1.8 – 5	6	HG1	6	HA	1.8 – 5
4	HA	5	HN	1.8 – 5	6	HG1	6	HD2	1.8 – 5
4	HA	4	HN	1.8 – 3.5	6	HG1	6	HD1	1.8 – 3.5
4	HB1	4	HN	1.8 – 3.5	6	HG2	6	HA	1.8 – 5
4	HB1	4	HA	1.8 – 3.5	6	HG2	6	HD2	1.8 – 3.5
4	HB1	5	HN	1.8 – 5	6	HG2	6	HD1	1.8 – 5

- The table format is the same with that of Supplementary Table 5.

Supplementary Table 18. $^{3}J_{HNCH}$ coupling values of DP2 in $CDCl_3$

Residue No.	Residue name	*J* (Hz)	ϕ (°)	Reason for N. D. (Not Determined)
1	Tyr	N. D.	N. D.	Signal overlap
2	Leu	N. D.	N. D.	ester (no amide NH)
3	D-Leu	8.3	92	
4	Leu	5.7	–72	
5	Leu	8.4	–93	
6	D-Pro	N. D.	N. D.	pro (no amide NH)

- Structure calculations were conducted with the ϕ ± 30° as restrictions.

Supplementary Reference

1. Deechongkit, S., You, S. & Kelly, J. W. Synthesis of All Nineteen Appropriately Protected Chiral α-Hydroxy Acid Equivalents of the α-Amino Acids for Synthesis. *Org. Lett.* **6**, 2209–2211 (2004).
2. Kuisle, O., Quiñoá, E. & Riguera, R. A general methodology for automated solid-phase synthesis of depsides and depsipeptides. Preparation of a valinomycin analogue. *J. Org. Chem.* **64**, 8063–8075 (1999).
3. Gude, M., Ryf, J. & White, P. D. An accurate method for the quantitation of Fmoc-derivatized solid phase supports. *Lett. Pept. Sci.* **9**, 203–206 (2002).
4. Biron, E., Chatterjee, J. & Kessler, H. Optimized selective N-methylation of peptides on solid support. *J. Pept. Sci.* **12**, 213–219 (2006).
5. Peraro, L. *et al.* Cell Penetration Profiling Using the Chloroalkane Penetration Assay. *J. Am. Chem. Soc.* **140**, 11360–11369 (2018).
6. Rezai, T., Yu, B., Millhauser, G. L., Jacobson, M. P. & Lokey, R. S. Testing the conformational hypothesis of passive membrane permeability using synthetic cyclic peptide diastereomers. *J. Am. Chem. Soc.* **128**, 2510–2511 (2006).
7. Ono, S. *et al.* Conformation and Permeability: Cyclic Hexapeptide Diastereomers. *J. Chem. Inf. Model.* **59**, 2952–2963 (2019).
