# Supplementary material for: Amide-to-ester substitution as a stable alternative to N-methylation for increasing membrane permeability in cyclic peptides
Source: Nat Commun. 2023 Mar 17;14:1416. doi: 10.1038/s41467-023-36978-z (PMC10023679; doi:10.1038/s41467-023-36978-z)
Supplement: Supplementary file 3 — Reporting Summary [file 41467_2023_36978_MOESM3_ESM.pdf]

nature portfolio

Corresponding author(s): Shinsuke Sando

Last updated by author(s): Jan 27, 2023

Reporting Summary

Nature Portfolio wishes to improve the reproducibility of the work that we publish. This form provides structure for consistency and transparency in reporting. For further information on Nature Portfolio policies, see our Editorial Policies and the Editorial Policy Checklist.

Statistics

For all statistical analyses, confirm that the following items are present in the figure legend, table legend, main text, or Methods section.

n/a	Confirmed	
[ ]	[x]	The exact sample size (*n*) for each experimental group/condition, given as a discrete number and unit of measurement
[ ]	[x]	A statement on whether measurements were taken from distinct samples or whether the same sample was measured repeatedly
[ ]	[x]	The statistical test(s) used AND whether they are one- or two-sided<br>*Only common tests should be described solely by name; describe more complex techniques in the Methods section.*
[x]	[ ]	A description of all covariates tested
[x]	[ ]	A description of any assumptions or corrections, such as tests of normality and adjustment for multiple comparisons
[ ]	[x]	A full description of the statistical parameters including central tendency (e.g. means) or other basic estimates (e.g. regression coefficient) AND variation (e.g. standard deviation) or associated estimates of uncertainty (e.g. confidence intervals)
[ ]	[x]	For null hypothesis testing, the test statistic (e.g. *F*, *t*, *r*) with confidence intervals, effect sizes, degrees of freedom and *P* value noted<br>*Give P values as exact values whenever suitable.*
[x]	[ ]	For Bayesian analysis, information on the choice of priors and Markov chain Monte Carlo settings
[x]	[ ]	For hierarchical and complex designs, identification of the appropriate level for tests and full reporting of outcomes
[x]	[ ]	Estimates of effect sizes (e.g. Cohen's *d*, Pearson's *r*), indicating how they were calculated

Our web collection on statistics for biologists contains articles on many of the points above.

Software and code

Policy information about availability of computer code

Data collection	Topspin 3.5 or 4.1 software (Bruker). Guava easyCyte 8 (Merck Millipore). Delta 5.0.4 (JEOL). MassLynx v4.2 (Waters). Leica Application Suite X (Leica). AMBER 20. Canvas (Schrodinger). Molecular Operating Environment 2019.01 (CCG).
Data analysis	Topspin 3.5 or 4.1 software (Bruker). GuavaSoft 2.7 (Merck Millipore). Delta 6.0.0 (JEOL). Poky. MassLynx v4.2 (Waters). ImageJ 1.53a (NIH). Visual Molecular Dynamics 1.9.3.

For manuscripts utilizing custom algorithms or software that are central to the research but not yet described in published literature, software must be made available to editors and reviewers. We strongly encourage code deposition in a community repository (e.g. GitHub). See the Nature Portfolio guidelines for submitting code & software for further information.

Data

Policy information about availability of data

All manuscripts must include a data availability statement. This statement should provide the following information, where applicable:

- Accession codes, unique identifiers, or web links for publicly available datasets
- A description of any restrictions on data availability
- For clinical datasets or third party data, please ensure that the statement adheres to our policy

The authors declare that the data supporting the findings of this study are available within the article and its supplementary information files. The raw data for assays, measurements, and simulations are available from the corresponding authors upon request.

Human research participants

Policy information about studies involving human research participants and Sex and Gender in Research.

Reporting on sex and gender	N/A
Population characteristics	N/A
Recruitment	N/A
Ethics oversight	N/A

Note that full information on the approval of the study protocol must also be provided in the manuscript.

Field-specific reporting

Please select the one below that is the best fit for your research. If you are not sure, read the appropriate sections before making your selection.

☒ Life sciences ☐ Behavioural & social sciences ☐ Ecological, evolutionary & environmental sciences

For a reference copy of the document with all sections, see nature.com/documents/nr-reporting-summary-flat.pdf

Life sciences study design

All studies must disclose on these points even when the disclosure is negative.

Sample size	No sample size calculation was performed prior to experiments. All the experiment was conducted in triplicate or quadruplicate. Some of the data ended up with smaller n numbers due to the detection limit.
Data exclusions	For PAMPA, the values that were under a quantification limit were excluded. For Caco-2 assay, the wells where the permeability of Lucifer yellow is above 2x10^-6 cm/s were excluded.
Replication	The CLSM experiment in Figure 2g was repeated once with some modifications and a similar result was obtained (Supplementary Figure 9). For most of the other experiments such as permeability assays, experiments were conducted in n=2-4.
Randomization	Randomization is not relevant to this study.
Blinding	This work does not contain experiments that require blinding.

Reporting for specific materials, systems and methods

We require information from authors about some types of materials, experimental systems and methods used in many studies. Here, indicate whether each material, system or method listed is relevant to your study. If you are not sure if a list item applies to your research, read the appropriate section before selecting a response.

Materials & experimental systems

n/a	Involved in the study	
☒	☐	Antibodies
☐	☒	Eukaryotic cell lines
☒	☐	Palaeontology and archaeology
☒	☐	Animals and other organisms
☒	☐	Clinical data
☒	☐	Dual use research of concern

Methods

n/a	Involved in the study	
☒	☐	ChIP-seq
☐	☒	Flow cytometry
☒	☐	MRI-based neuroimaging

Eukaryotic cell lines

Policy information about cell lines and Sex and Gender in Research

Cell line source(s)	HeLa cells from RIKEN cell bank / Caco-2 cells (HTB-37) from ATCC
Authentication	None of the cells were authenticated after obtained from the distributors.
Mycoplasma contamination	The cell lines were not tested for micoplasma contamination after obtained from the distributors.
Commonly misidentified lines (See ICLAC register)	There are no commonly misidentified lines used for this study.

Flow Cytometry

Plots

Confirm that:

- [x] The axis labels state the marker and fluorochrome used (e.g. CD4-FITC).
- [x] The axis scales are clearly visible. Include numbers along axes only for bottom left plot of group (a 'group' is an analysis of identical markers).
- [x] All plots are contour plots with outliers or pseudocolor plots.
- [x] A numerical value for number of cells or percentage (with statistics) is provided.

Methodology

Sample preparation	The treated HeLa cells were incubated with TrypLE express at 37 °C for 10 min. After addition of RPMI containing 10% FBS and 1% Antibiotic-Antimycotic (Nakalai), the cells were collected to a test tube and washed with PBS.
Instrument	Guava easyCyte 8 (Merck Millipore)
Software	GuavaSoft 2.7
Cell population abundance	The cells were gated using the criteria described below and all the gated cells were used for the analysis.
Gating strategy	Determine the live cell region based on the FSC/SSC ratio with reference to the control cells that were not treated with any peptides. The live cells were gated and the ratio of SiR-ct/Haloenzyme-GFP (mean fluorescence intensity of each) was plotted.

- [x] Tick this box to confirm that a figure exemplifying the gating strategy is provided in the Supplementary Information.
